# Phylogenetic analysis of the *Tc1/mariner* superfamily reveals the unexplored diversity of *pogo*-like elements

**DOI:** 10.1186/s13100-020-00212-0

**Published:** 2020-06-29

**Authors:** Mathilde Dupeyron, Tobias Baril, Chris Bass, Alexander Hayward

**Affiliations:** Centre for Ecology and Conservation, University of Exeter, Penryn Campus, Penryn, Cornwall, TR10 9FE UK

**Keywords:** Transposase, DNA transposon, Transposable element, Evolution, Phylogeny, *Tigger*

## Abstract

**Background:**

*Tc1/mariner* transposons are widespread DNA transposable elements (TEs) that have made important contributions to the evolution of host genomic complexity in metazoans. However, the evolution and diversity of the *Tc1/mariner* superfamily remains poorly understood. Following recent developments in genome sequencing and the availability of a wealth of new genomes, *Tc1/mariner* TEs have been identified in many new taxa across the eukaryotic tree of life. To date, the majority of studies focussing on *Tc1*/*mariner* elements have considered only a single host lineage or just a small number of host lineages. Thus, much remains to be learnt about the evolution of *Tc1/mariner* TEs by performing analyses that consider elements that originate from across host diversity.

**Results:**

We mined the non-redundant database of NCBI using BLASTp searches, with transposase sequences from a diverse set of reference *Tc1/mariner* elements as queries. A total of 5158 *Tc1/mariner* elements were retrieved and used to reconstruct evolutionary relationships within the superfamily. The resulting phylogeny is well resolved and includes several new groups of *Tc1/mariner* elements. In particular, we identify a new family of plant-genome restricted *Tc1/mariner* elements, which we call *PlantMar*. We also show that the *pogo* family is much larger and more diverse than previously appreciated, and we review evidence for a potential revision of its status to become a separate superfamily.

**Conclusions:**

Our study provides an overview of *Tc1-mariner* phylogeny and summarises the impressive diversity of *Tc1-mariner* TEs among sequenced eukaryotes. *Tc1/mariner* TEs are successful in a wide range of eukaryotes, especially unikonts (the taxonomic supergroup containing Amoebozoa, Opisthokonta, Breviatea, and Apusomonadida). In particular, ecdysozoa, and especially arthropods, emerge as important hosts for *Tc1/mariner* elements (except the *PlantMar* family). Meanwhile, the *pogo* family, which is by far the largest *Tc1/mariner* family, also includes many elements from fungal and chordate genomes. Moreover, there is evidence of the repeated exaptation of *pogo* elements in vertebrates, including humans, in addition to the well-known example of *CENP-B*. Collectively, our findings provide a considerable advancement in understanding of *Tc1/mariner* elements, and more generally they suggest that much work remains to improve understanding of the diversity and evolution of DNA TEs.

## Introduction

DNA transposable elements (TEs) or ‘class II elements’ are a major category of repetitive DNA. DNA TEs use a cut-and-paste mechanism catalysed by a transposase enzyme to mobilize within the host genome, and may comprise a considerable proportion of total host genomic DNA [1–3]. DNA TEs typically contain a transposase domain enclosed by terminal inverted repeats (TIRs). However, this organisation is flexible and TEs may contain additional ORFs or motifs, and in some cases they can lack TIRs. TIRs act as recognition sites for the transposase enzyme, which excises the transposon and relocates it to a new position within the genome during transposition.

The *Tc1/mariner* superfamily is an important group of DNA TEs discovered in invertebrate genomes during the early 1980s, and is considered to be the most widespread DNA TE superfamily among eukaryotes [4]. The first *Tc1/mariner* element discovered was *Tc1,* during examination of restriction fragment strain polymorphisms in the nematode roundworm *Caenorhabditis elegans* in 1983 [5]. Three years later, the *mariner* element was identified in the fruitfly *Drosophila mauritiana* during study of the *white-peach* (*w*^*pch*^) eye colour mutant [6]. In 1990, a bacterial insertion sequence found in the *Shigella* genome, IS630, was linked to *Tc1*, as it shares a TA target site duplication (TSD) formed after successful transposition [7]. At first considered to represent different families, *Tc1*, *mariner*, and *IS630* were later gathered together as the *IS630/Tc1/mariner* (ITm) group, based on their shared mode of transposition via a DNA intermediate, their TA target site, and transposase sequence homology [8, 9]. Soon after, the *pogo* family was also classified as a member of ITm [8]. *Pogo* was characterised in the genome of the fruitfly *D. melanogaster,* during a study of the promoter region of the *white* locus, where two insertions, one of them a *pogo* element, caused the *white-eosin* (*w*^*e*^) mutation [10]. In the late 1990s, *mariner*-like elements were discovered in plant genomes, starting with soybean [11].

In total, eight families are currently included within the ITm group, which are classified according to the number of amino acid residues present between the second and third aspartic acid residue (D), or the second aspartic acid and the glutamic acid residue (E) of the transposase catalytic domain (i.e. DDD/E) [12]. The eight described ITm families are: *mariner* (DD34D), *Tc1* (DD34E), *pogo* (DDxD), *DD39D* from plants, *DD37E* from mosquitoes, *DD37D* from insects and nematodes, *DD34E* from ciliates, and the bacteria insertion sequence group *IS630* [12, 13]. However, the branch linking the *DD34E* family to the other families is poorly supported, and its membership to the group is considered questionable (Fig. 4 in [12]). Furthermore, bacterial *IS630* sequences are only distantly related to eukaryotic DNA TEs and are not considered to be similar to eukaryotic DNA TEs. Thus, here we restrict our focus to eukaryotic *Tc1/mariner* elements, and consider *IS630* as the outgroup to the *Tc1/mariner* superfamily.

The *Tc1/mariner* superfamily is well known due to the widespread use of several *Tc1/mariner* elements as genetic tools. For example, *Sleeping Beauty* (*SB*) is a synthetic TE reconstructed from multiple inactive fish *Tc1*-like transposon sequences, that is widely used in genetic engineering for somatic gene delivery and functional genomics (e.g. gene discovery) [14]. Similarly, a *Tc1*-like element found in the *Rana pipiens* genome called *Frog Prince* was reconstructed for gene-trapping in fish, amphibians and mammals [15]. The first *mariner* element to be used as a genetic tool was *Hsmar1*, which was reconstructed from the human genome [16]. *Hsmar1* transposes efficiently in vertebrate cells and has been linked to the formation of non-autonomous MITE elements, making it a useful system to study the transposition dynamics and evolution of *mariner* elements in primate genomes [17]. Additionally, the fungal transposon *Fot1* of the *pogo* family is used as a tagging system to study the regulation of gene expression in fungi [18].

Adding to the fame of *Tc1/mariner* elements, the superfamily includes several high profile examples of the molecular domestication of transposon sequences for host genomic purposes. For example, *SETMAR* is a chimeric gene that is expressed in most cells and tissues in anthropoid primates, which has roles in key processes such as DNA methylation, repair and alternative splicing [19]. *SETMAR* is composed of a *SET* gene, shared among vertebrates, and an *Hsmar1* transposase. The transposase is flanked by a 3′ TIR and an *Alu* retrotransposon on the 5′ end, and transposition is estimated to have occurred 40–58 million years ago in an ancestral lineage of the anthropoid primates [20]. Another important example of the molecular domestication of a *Tc1/mariner* element is *centromere protein B* (*CENP-B*), a conserved protein found in mammalian centromeres [21]. *CENP-B* appears to have been domesticated from a *Tigger-*like element (from the *pogo* family), which belongs to a group of *Tc1/mariner* elements that contain a *CENP-B* box in their 5′ TIR [21]. The *CENP-B* protein binds the *CENP-B* box which, in addition to being located in *Tigger*-like elements, is also located in host alpha-satellite centromeric DNA [22], and is thought to be involved in kinetochore formation (although its exact role in centromere functioning remains unclear) [23].

Despite the importance, diversity, and very large host range of *Tc1/mariner* elements, there are no recent studies of their evolution and classification. Several publications report the diversity of a subset of *Tc1/mariner* elements from the genomes of a focal group of organisms [24–27]. However, the most comprehensive phylogenetic analysis of the evolution of the *Tc1/mariner* superfamily, including most known elements of each family and clade support values, was published in 2001 [12]. Here we employ the many new *Tc1/mariner* sequences detected in recently sequenced eukaryote genomes to perform a large-scale phylogenetic analysis of the *Tc1/mariner* superfamily, which we use to examine the evolution, diversity, and classification of the group.

## Results and discussion

**Fig. 1:**
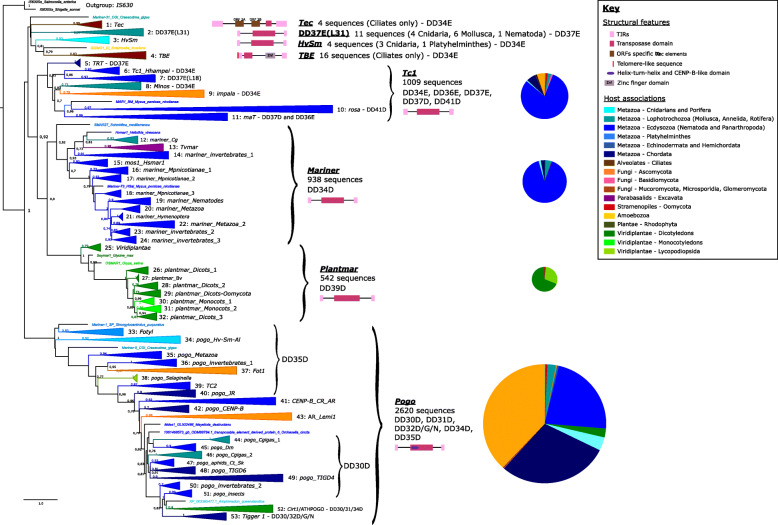
Schematic providing a summary of host associations for monophyletic *Tc1/mariner* groups identified during phylogenetic analysis, which are illustrated as collapsed clades. For each clade (except groups containing less than 3 sequences), a schematic summarising the structure of the TEs contained within each group is illustrated, with structural features represented by different coloured rectangles (please see the accompanying key). TIR: terminal inverted repeat, ORF: open reading frame; Znf: zinc finger domain. For the *Tc1, PlantMar, mariner* and *pogo* families, pie charts show the proportion of elements extracted from each eukaryote host group. The four minor families *Tec*, *HvSm*, DD37E(L31) and *TBE* do not have pie charts as the number of sequences is very small

We recovered 5158 *Tc1/mariner* elements from the genomes of 922 species from across eukaryotic diversity, adding greatly to the known diversity of the *Tc1/mariner* superfamily (Additional file 1). Based on the results of our phylogenetic analyses (Fig. 1, Additional File 2), we present the evolutionary relationships among eight families that form the *Tc1/mariner* superfamily. We identify four previously characterised major families, each of which contain a large number of sequences: *Tc1*: 1009 sequences; *mariner*: 938 sequences; *PlantMar* (formerly referred to as ‘*DD39D*’): 542 sequences; *pogo*: 2620 sequences, and four minor families that contain just a few sequences each: *Tec*: 4 sequences; *TBE*: 16 sequences; *DD37E(L31)*: 11 sequences; *HvSm*: 4 sequences (Fig. 1, Additional File 2). The four minor families include three previously described families (i.e. *DD37E(L31), Tec*, *TBE*) and one new family, which we name *HvSm*, after its host species (*Hydra vulgaris* and *Schmidtea mediterranea*).

We show that *Tc1/Mariner* phylogeny is composed of two main clades, one containing the *Tc1, mariner* and *PlantMar* families plus the four minor families, and one entirely composed of the *pogo* family. The *pogo* family is separated from the other *Tc1/mariner* families by a relatively long branch, indicating considerable evolutionary distinctness. Further, we reveal that *pogo* contains a very large number of elements (more than double the remaining *Tc1/Mariner* elements), which were isolated from the genomes of a diverse array of host taxa, and these elements feature a helix-turn-helix and CENP-B-like domain. Taking these features into account, we suggest below that *pogo* may be more appropriately classified as its own superfamily.

The main *Tc1/mariner* clade consists of a poorly-supported grouping, which unites four minor families that share a sister-group relationship with a well-supported clade containing the *Tc1, mariner,* and *PlantMar* families. The host taxonomic origin of the main *Tc1/mariner* clade is unclear, but there is clear evidence of a single transition to plant hosts leading to the origin of the *PlantMar* family (groups 25–32). Meanwhile, more basal subclades in the *pogo* clade contain DNA TEs of metazoan origin, suggesting an origin in animals (possibly flatworms or cnidarians), with two independent transitions to plant genomes: a small clade involving the club moss *Selaginella* (Clade 38), and a larger clade containing a number of dicotyledonous plant hosts (Clade 52).

Several isolated taxa consisting of a single transposase sequence are scattered across *Tc1/mariner* phylogeny (Fig. 1). These sequences typically occur at the end of relatively long branches, and it is possible that they are sole representatives of additional families, which will become clearer in the future as genomic data continue to accumulate. Below we discuss the major patterns observed in the *Tc1/mariner* phylogeny in more detail, alongside accompanying insights gained about the evolution of this large and important DNA TE superfamily.

### The *Tc1* family

The *Tc1* family is well-supported with 93% bootstrap support, and is composed of seven subfamilies (Fig. 1). The majority of subfamilies within *Tc1* have ≥85% bootstrap support, although the *Minos* and *impala* subfamilies have 77 and 79% support respectively (Fig. 1). Subfamilies are named in accordance with previously described groups or their main host genomes (Fig. 1).

*Tc1* elements share a similar structure, with TIRs of ~ 30 bp, a single ORF encoding a transposase, and a mean length of ~ 1300 bp [9, 28, 29]. However, some *Tc1* elements have long TIRs of several hundred base pairs, such as *Minos* [30, 31]. We confirm the DDD/E structure identified previously for the different *Tc1* families: *TRT* (Group 5) is DD37E, *Tc1_Hhampei* (Group 6) and *Minos* (Group 8) are DD34E, *impala* (Group 9) is DD37E, *rosa* (Group 10) is DD41D, and *maT* (Group 11) is DD37D (Additional file 3A). We identify a large number of newly identified sequences from host genomes including arthropods, fungi, archeaplastids, oomycetes and several bacteria (in black, Additional file 2). The apparent presence of a very small number of TEs apparently isolated from bacterial genomes within the otherwise eukaryote-restricted *Tc1/mariner* superfamily suggests either their horizontal transfer across major domains of life, or that contamination events have occurred (and bacterial host associations are spurious). To attempt to discriminate between these alternatives and confirm integration in a bacterial genomic context, we searched for flanking sequences in the corresponding elements in Genbank. Either no flanking sequence was present (i.e. only partial TE sequence existed without TIRs), or only very short up- or downstream sequences were present. Thus, we suggest that in the absence of evidence for horizontal transmission, it is prudent to consider these sequences as contamination or erroneously labelled with regard to their origin.

This study confirms the success of *Tc1* elements in a diverse range of organisms, but especially ecdysozoan metazoans (i.e. arthropods and nematodes) (Additional file 2). The impressive number of sequences recovered from the spider *Stegodyphus mimosarum* (Clade 11, Additional file 2), together with a pattern of very short terminal branch lengths, suggest these elements underwent a recent expansion or are still undergoing a significant burst of activity in this taxon. The little brown bat, *Myotis lucifugus,* is the only mammalian vertebrate identified by our analyses that possesses *Tc1* sequences (Clade 11, Additional file 2). The *M. lucifugus* genome is of particular interest, because in contrast to other mammalian genomes, it contains recently active DNA TEs [1]. Given that *M. lucifugus* feeds on a very wide range of arthropods (at least 61 insect species and 5 spider species [32]), its diet may increase its exposure to TEs from arthropods and the likelihood of horizontal transmission. The *S. mimosarum* and *M. lucifugus Tc1* sequences are present in the *maT* subfamily (Clade 11, Fig. 1), which contains elements from an unusual grouping of hosts including red algae, oomycetes, basidiomycetes, mycorrhizal fungi, and microsporidians (Additional file 2). Such a patchy taxonomic distribution, together with the parasitic lifestyle of several of these host organisms, suggests a history of horizontal transfer in this group [33]. For example, parasitic organisms such as oomycetes may facilitate horizontal transfer, since they are widespread pathogens that share an intimate association with their hosts, they occupy varied environments, and they infect an extremely large host range [34]. Further study is required to elucidate the relationships between similar *Tc1* elements shared by extremely divergent host taxa, and addressing this question will become more straightforward as genomic data from a wider sampling of host diversity accumulates.

A recent study of *Tc1/mariner* elements in the genome of the Pacific oyster *Crassostrea gigas* identified a new subfamily and a new family of elements. Specifically, these new groups were described as a: (i) a *Mariner-18_CGi*-like group, *DD37E(L18),* which appeared to form a new subfamily within the *Tc1* family; and, (ii) a *Mariner-31_CGi*-like group, *DD37E(L31)*, that appeared to form a whole new family more basal to *pogo* [35]. We included the sequences from this analysis in our sequence alignment (Additional file 4), and found that: (i) the *DD37E(L18)* subfamily is present in the *Tc1* family, but unfortunately it does not group together with *Mariner-18_CGi* (the *C. gigas* sequence it was named after) which occurs in *Minos* (Group 8, Additional File 2); while, (ii) the *DD37E(L31)* family shares a sister-group relationship with the *Tec* subfamily, and again unfortunately does not group together with *Mariner-31_CGi,* which groups as an isolated sequence with the minor families *Tec, DD37E(L31), HvSm* and *TBE* (Fig. 1, Additional file 2). This situation highlights the difficulties associated with describing new TE families on the basis of single taxon studies, and the problematic nomenclature that can arise as a consequence.

### The *mariner* family

*Mariner* is a very well known family of *Tc1/mariner* elements, and previous studies have revealed its wide distribution among metazoans, partly as a consequence of horizontal transmission [36]. Most *mariner* elements are short transposons of ~ 1200-1300 bp, that contain a single ORF encoding a DD34D transposase (Additional file 4), with short ~ 20-30 bp TIRs.

Our analysis demonstrates that *mariner* elements are present primarily in arthropod genomes, and we identify a great many more elements than previously known. Nevertheless, several elements in the *mariner* clade also originate from the genomes of a diverse range of other taxa (i.e. excavates*,* platyhelminths, gastropods, nematodes, and rotifers). In total, we update the number of *mariner* subfamilies to 13 (Additional File 2).

Previous studies have tended to focus on *mariner* elements from particular host groups, for example hydra and flatworms [37] or aphids [27]. Where applicable, we maintain the names adopted for previously identified subfamilies in our phylogeny, but in some cases elements representing these groups are located in multiple subfamilies together with numerous sequences from invertebrate genomes, and we have renamed them accordingly (Additional File 2). These differences result from the greater host diversity considered here and the many numerous new sequences we identify in the *mariner* family. Thus, while studies focussing on single host taxa can be useful to identify new TE diversity within a particular host genome, going forward we suggest a cautious approach toward suggesting new families, based on broad scale analyses that consider a wide range of host taxonomic diversity only.

A small number of elements that are labelled as originating from bacterial host genomes are also present within the *mariner* family, but we could not find any evidence to suggest horizontal transfer, leading us to conclude that these sequences most likely represent labelling errors or contamination, as in the *Tc1* clade.

### The *PlantMar* family

The few studies that have analysed sequences from the *Tc1/mariner* DD39D group typically included them as members of the *mariner* family [11, 38]. In contrast, our analysis facilitated the retrieval of many additional sequences from this group, which almost all originate from plant genomes. We find strong evidence for the existence of a separate plant *Tc1/mariner* family that we name ‘*PlantMar*’ (Clades 25–32, Fig. 1). The *PlantMar* family forms a monophyletic group with full bootstrap support that originates at the end of a long-branch, with a sister-group relationship to the *mariner* family.

The structure of *PlantMar* elements is typical for *Tc1/mariner*, with short TIRs, an overall length of 2-6 kb, and a DD39D transposase domain amino acid structure (Additional File 4). The *PlantMar* family is restricted to dicotyledonous and monocotyledonous plant hosts, and a small number of oomycete hosts [39]. We identified 8 subfamilies in the *PlantMar* family, with elements generally clustering according to whether their host plant is mono- or dicotyledonous (Fig. 1, Additional file 2). None of the elements in the *PlantMar* family belong to early plant phyla such as Glaucophytes (freshwater microscopic algae), Rhodophyta (red algae), Zygnematales (green algae) or Bryophyta (liverworts, hornworts and mosses), leading us to formulate two alternative hypotheses, either these elements: (i) underwent horizontal transfer to angiosperm genomes, most likely from an ancestral *Tc1/mariner* element present in Oomycota, fungal pathogens and/or viruses; or, (ii) were once present in Archaeplastida and were subsequently lost in early-branching phyla, remaining present in higher Viridiplantae genomes only. Given the host taxonomic context present in the closely related *Tc1* and *mariner* families (i.e. a widespread distribution across arthropods), hypothesis (i) appears is more likely, suggesting horizontal transfer followed by a host switch, leading to the origin of a distinct family restricted to plants.

Interestingly, as with evolution of the *Mutator* DNA TEs [40], once a host switch to plants occurred, the ability to switch back to other branches of eukaryotic life seems to have been almost completely lost. With the exception of a few sequences from Oomycota, we find no transitions back to non-plant hosts within the *PlantMar* clade. Given that there are now at least two examples of DNA TEs making strict unidirectional switches onto plant hosts, it will be interesting to examine if this pattern is repeated across a wider swathe of DNA TE diversity, and to investigate the mechanisms that prevent a switch back from plants to a wider diversity of hosts in these cases.

### *TBE* and *Tec* from ciliates

Two small *Tc1/mariner* subfamilies are restricted to ciliates: *Tec* and *TBE* (Clades 1 and 4, Fig. 1, Additional file 2). We maintained the names previously used in the literature for both subfamilies [41, 42]. *TBE* (telomere bearing elements) is named for the presence of telomere-like sequences at the tips of the TIRs, and the *TBE* clade is sister to the *GIZMO* element identified from the amoeba *Entameoba invadens* [41]. Elements in the *TBE* family have small ~ 80 bp TIRs and they carry three ORFs: a transposase, a small ORF of unknown function, and a zinc finger protein [43].

*Tec* elements have a highly unusual structure for DNA transposons, with very long TIRs of ~ 700 bp and three ORFs, one of them in complement [42]. Despite these differences from typical *Tc1/mariner* transposons, *Tec* and *TBE* encode a transposase containing a DD34E motif in the third ORF [44]. Moreover, *Tec* elements carry a site-specific recombinase in the second ORF which can perform transposition in the absence of a dedicated transposase [45]. This is consistent with the very short third ORF that carries the transposase, which may be inactive.

Ciliates are peculiar eukaryotes with cells containing two nuclei: a micronucleus containing the germline, which is mostly transcriptionally inactive and has a TE content of ~ 20%, and a somatic transcriptionally active macronucleus without repeats [46]. During formation of the macronucleus, the non-coding part of the micronuclear DNA is deleted, including the transposon content, leading to the formation of ‘internal eliminated sequences’ (IESs), with TSD-like sequences corresponding to TE remnants [43]. The precise mechanisms of macronucleus formation and *TBE* and *Tec* element transposition remain unclear. A signature of purifying selection detected for ORFs present in *TBE* elements suggests that the excision activity of the transposase may have been harnessed by the host genome, for example in the elimination process of non-coding sequences during macronucleus formation [42, 43]. The peculiar life cycle of ciliates may explain some of the unusual structural differences exhibited by *TBE* and *Tec* elements relative to *Tc1/mariner* TEs present in the genomes of other eukaryotes.

### *HvSm* - a new family with only four sequences

Three sequences from the freshwater cnidarian polyp *Hydra vulgaris,* and one sequence from the platyhelminth *Schmidtea mediterranea* form a new family that we name ‘*HvSm*’, reflecting its main host association (Clade 3, Fig. 1 and Additional file 2). Surprisingly, *HvSm* does not contain sequences from other cnidarian or platyhelminth species, despite the presence of 26 cnidarian genomes and 36 flatworm genomes in Genbank. Nevertheless, this remains a relatively small number of genomes compared to estimates of the total number of described platyhelminthes (> 18,000 [47]) and cnidarians (> 9000 [48]), and we anticipate that more elements will be identified in this family.

We analysed the structure of the four elements in *HvSm* and found that one of them is partial, containing a 579 bp transposase ORF, but no TIRs. The full-length copies contain 585 bp, 756 bp and 1106 bp transposases, with 15 bp, 20 bp and 56 bp TIRs flanked by TA TSDs, and have an overall length of 1716 bp, 1841 bp and 2751 bp, respectively (two full-length sequences are provided in Additional file 5). Each transposase shows a DD34E motif, similar to the closely related *Tec* and *TBE* families, but differentiating them from the *DD37E(L31)* family.

### The *pogo* family

The most recent consideration of the *pogo* family was in 2014, which included 60 sequences from the genomes of 38 host species, belonging to 3 kingdoms of eukaryotic life [49]. In contrast, we have retrieved 2620 *pogo*-like sequences from the genomes of 519 host species, belonging to six kingdoms of eukaryotic life (Fig. 1). Contrary to the *Tc1* and *mariner* families, which occur predominantly in ecdysozoan genomes*, pogo* elements are also found in many ascomycete fungus and chordate genomes (Fig. 1). Thus, we reveal that the *pogo* family is dispersed across a considerably wider diversity of hosts than previously appreciated. Additionally, we find that *pogo* is the largest *Tc1/mariner* family, containing more than half the total number of all *Tc1/mariner* elements recovered in our study (Fig. 1 and Additional file 2).

Notably, we identified distinct DDD motifs in the transposase domains of *pogo* elements: groups 33 to 40 display a DD35D pattern; groups 44 to 51 display a DD30D pattern; while groups 52 and 53 show a varying pattern of DD30-32D (Fig. 1 and Additional file 3B). This reveals new information about the structure of the *pogo* transposase catalytic domain, contrasting with what has been described previously. Groups 41 to 43 do not show a specific pattern, with sequences displaying DD30D, DD31D or DD34D motifs, with no apparently dominant type.

Taking the range of new evidence into account, we suggest that *pogo* may be more appropriately classified as an independent superfamily of DNA TEs, instead of a family within the *Tc1/mariner* superfamily. This evidence includes: the relatively long branch length leading to the *pogo* clade, its distinct pattern of host associations, the size of the *pogo* family, its sister group relationship to a clade containing all other *Tc1/mariner* elements, the distinct transposase structure of *pogo* elements where the transposase domain contains a helix-turn-helix and CENP-B-like domain, and the distinct pattern of DDD motifs in the catalytic domain of *pogo* transposases.

Previous studies revealed the presence of *pogo*-like elements in mammals, reptiles, fish, insects, nematodes, molluscs, fungi and plants [8, 18, 35, 49, 50], and classified them in five main groups: TC2 (human and fish), AR (plants and fungi), JR (metazoans), CR (metazoans) and *Fot1* (fungi) [22, 49]. Our phylogenetic analysis indicates the presence of 21 *pogo* subfamilies (Fig. 1 and Additional file 2), most of which contain sequences from a single host kingdom (e.g. metazoans or fungi, Fig. 1, Additional File 2). Several previously described groups (e.g. AR, JR and CR [22]) are not monophyletic in our phylogeny, and the sequences from these groups are instead scattered across various *pogo* subfamilies (Fig. 1 and Additional file 2). More basal subgroups contain invertebrate sequences and many fungal sequences, especially from *Fusarium,* from which *Fot1* was described (Clade 37, Fig. 1 and Additional file 2). Below we provide a short description of several notable subfamilies, drawing attention to particular points of interest.

Clade 33 is composed of a small set of sequences originating from diverse organisms, such as Amoebozoa, Fungi, and Echinodermata. Several small subgroupings contain sequences from distantly related terrestrial and aquatic organisms, resulting in a diverse and somewhat puzzling host distribution pattern. Meanwhile, the large *Fot1* subfamily consists mainly of fungal sequences, however, one subgroup contains six sequences from the Pacific oyster *Crassostrea gigas*. Oysters live in aquatic marine environments, whereas fungal species containing *pogo*-like elements that group closely to oyster *Fot1*-like elements are terrestrial. Clade 38 is composed of only two sequences from the lycophyte plant *Selaginella moellendorffii*. This model species is a primitive vascular plant and is of interest because it is a very ancient group [51]. *S. moellendorffii* has one of the smallest genomes known among plants and more than a third of its genome is composed of TEs [52]. The detection of two *pogo*-like sequences in such an early vascular plant genome presents two hypotheses: (i) *pogo* elements are ancient DNA TEs that were present in early eukaryote taxa, and have subsequently undergone elimination in most plant lineages, but were successfully retained in many unikonts (i.e. Metazoa and Fungi); or alternatively, (ii) *pogo* elements underwent multiple independent horizontal transfer events to plant genomes, leading to their presence in several dicotyledonous plant genomes and *S. moellendorffii*. Further research into the activity of *pogo*-like elements in early plant taxa is needed to clarify their evolution in this group. *TC2* elements were originally described in the puffer fish, *Takifugu rubripes,* and the human genome (Clade 39, Fig.1 and Additional file 2). We find closely related *TC2*-like elements from a diverse host range in our phylogeny: *Helobdella robusta* a leech species, various vertebrates (fish, gecko, snake and lemur), a mite, a beetle, and many sequences from the spider *Stegodyphus mimosarum*. Either this pattern is a result of the presence of the ancestral element in the common ancestor of Bilateria, followed by loss in most bilaterian lineages, or a consequence of repeated horizontal transfer events.

We identify considerable confusion surrounding the classification of *Tigger* elements. The first *Tigger* elements, *Tigger1* and *Tigger2*, were isolated from mammalian genomes, and were described with reference to their similarity to CENP-B and *pogo* elements more widely [10, 21]. *Tigger1* was specifically classified as a ‘mammalian *pogo*’ [10], which we confirm here. However, we reveal that *Tigger1* (Clade 53, Additional file 2), and *Tigger2* (Clade 40, Additional file 2) are separated by considerable phylogenetic distance in our analysis. Thus, while the transposase sequences of *Tigger1* and *Tigger2* are similar, the availability of a much larger number of *pogo-*like elements now demonstrates that *Tigger1* and *Tigger2* are relatively distantly related within a wider evolutionary context. Further, since the original canonical *pogo* element occurs in a clade situated between *Tigger1* and *Tigger*2 (Clade 45, Additional file 2), it is apparent that *Tigger* elements are polyphyletic, and do not form a distinct monophyletic group. Additionally, over time the use of sequence similarity to classify elements has led to the annotation of new *Tigger*-like elements across a large swathe of *pogo*-like element diversity (Clade 40, 43, 49, 52, and 53, Additional file 2), and we suggest that this practice is abandoned in favour of phylogenetic approaches.

### Domestication of *pogo* elements

*Pogo* transposases are known to have been exapted for host functions in metazoan genomes, with a well-known example being the evolution of centromeric protein *CENP-B* [22]. We find evidence for additional domestications in several other *pogo* lineages. Specifically, we provide evidence of exaptation for seven *pogo*-like elements, which are frequently referred to as ‘*Tigger transposable element derived’* genes (*TIGD1-TIGD7*) [53]*,* in tetrapod host genomes, especially mammals.

We checked the genomic context of the human *TIGD6* gene in Ensembl [54] and identified the nearest upstream and downstream genes: *SLC26A2*, a solute carrier transporter, and *HMGXB3*, a DNA binding protein. We then used the orthology verification tool in Ensembl using the human *TIGD6*, *SLC26A2* and *HMGXB3* genes as queries to identify conservation in their arrangement in the genomes of other mammal species (Additional file 11). Similarly, the seven *TIGD* genes all show syntenic organisation in mammals and other vertebrates (reptiles, amphibians and birds) (Additional files 6, 7, 8, 9, 10, 11 and 12). The shared genomic organisation of the *TIGD*-like elements in vertebrate genomes suggests an ancient insertion event in an ancestral vertebrate. Detailed information for each of these *TIGD*-like elements and their genomic environment is provided in Additional files 6, 7, 8, 9, 10, 11 and 12.

*TIGD1-TIGD7* display full-length ORF sequences corresponding to the transposase domain, suggesting conservation of functionality, and likely exaptation for host genomic purposes. In *Homo,* BioGrid [55] lists 3 protein-protein interactions for *TIGD1*, 3 for *TIGD2*, 4 for *TIGD3*, 3 for *TIGD4*, 50 for *TIGD5*, 21 for *TIGD6*, and 5 for *TIGD7*. Thus, there is good experimental evidence that *TIGD* proteins often interact with large numbers of host proteins, suggesting an embedded role for *TIGD* genes in the host genome context. Further, the Bgee [56] and Genevisible [57] gene expression databases suggest that *TIGD* genes are widely expressed in *Homo*: *TIGD1–*168 organs, with highest expression in the brain and immune cells; *TIGD2–*178 organs, with highest expression in the placenta; *TIGD3–*63 organs, with highest expression in the cerebellar hemisphere, blood and leukocytes; *TIGD4–*103 organs, with highest expression in sperm and the testes; *TIGD5–*203 organs, with highest expression in the quadriceps femoris muscle, the deltoid muscle, the parotid gland, and the epithelium of the nasal cavity and kidney; *TIGD6–*136 organs, with highest expression in the prostate gland, spinal cord, and across the endocrine system; and *TIGD7–*175 organs, with highest expression in the testis.

Considering the pattern of divergent *TIGD*-like sequences in our phylogenetic tree (Additional file 2), we performed a NCBI BLASTp search focussing on *TIGD*-like sequences, using coding sequence corresponding to the ancestral transposase domain of each *TIGD1–7* gene as queries. We then performed a phylogenetic analysis on the retrieved sequences to examine *TIGD* diversity in more detail (alignment: Additional file 13, tree: Additional file 14). Below we briefly summarise the major patterns present in the *TIGD* tree and discuss their implications.

*TIGD*-like sequences occur in highly supported clades (≥97%) and are restricted to tetrapod hosts and their immediate relatives (i.e. the Coelacanth lobe-finned fish, *Latimeria chalumnae*) (Additional file 14). In several cases, closely related sequences from invertebrate hosts occur more basally to *TIGD* clades (i.e. *TIGD1, TIGD3, TIGD4, TIGD6*), suggesting multiple independent domestication events of different ancestral *pogo* elements (Additional file 14). In contrast, the *TIGD2, TIGD5*, and *TIGD7* clades are united together in a group, and it is possible that these genes may represent paralogues (i.e. be descended by gene-duplication from a single *pogo* domestication event). The host distribution of *TIGD* sequences within tetrapods remains patchy. Additionally, while sequences in several *TIGD* clades are widely distributed across tetrapod diversity (*TIGD1, TIGD4, TIGD5*), the taxonomic distribution of others are more narrow (for example, no sequences were identified from birds for *TIGD3*)*,* or are either partially restricted to mammals (*TIGD2*) or are entirely restricted to mammals (*TIGD6, TIGD7*). These findings suggest that either *TIGD* genes have been selectively retained in certain host lineages following an ancient origin pre-dating the tetrapods, or that sequences in certain host lineages have become too divergent for our current approach to recover. Detailed work to distinguish between these alternatives would be valuable to further illuminate *TIGD* evolution in the future.

The *TIGD* catalytic motif shows considerable variation, presumably as a consequence of positive selection following exaptation to optimise *TIGD* proteins for new roles in the host genome: *TIGD1* is DD32D; *TIGD2* is mostly DD34S; *TIGD3* is DA30P in mammals, DA35P in birds, and DD33H in reptiles; *TIGD4* is DD30K or DE30K in vertebrates, except birds where we could not identify the third position; *TIGD5* is DA60E in mammals, DS/T48E in birds, and DN33D in reptiles; *TIGD6* is mostly DD30N; and *TIGD7* is DD34N (Additional file 3B and C).

The evidence discussed above suggests that *TIGD* genes may play important fundamental roles in vertebrates and the group deserves closer research attention. We are conscious that our search will not have uncovered all *Tigger*-like elements, and intensive study of *TIGD* genes, particularly involving validation in the lab, will likely yield considerable further insights into their domestication and roles.

### Host range evolution and horizontal transfer

The majority of *Tc1/mariner* elements were recovered from ecdysozoan host genomes (i.e. arthropods and nematodes). However, host range often varies among families. Most dramatically, the *PlantMar* family contains elements from plants and stramenopiles only. Meanwhile, the *pogo* family includes large proportions of elements that originate from fungus and chordate genomes (Fig. 1). Interestingly, despite a relatively cosmopolitan distribution across eukaryotes, relatively few *Tc1/mariner* elements are present in more basal eukaryote lineages, for example, we identified just two elements in amoeboid protists (Amoebozoa), and no elements in green algae (Chlorophyta).

Very little is known about the mechanisms that underlie host range in TEs, and the patterns we observe for *Tc1/mariner* elements are no exception. For example, whether host range is primarily driven by encounter or by compatibility filers, sensu theory from host-parasite interactions [58], remains very much an open research question.

**Fig. 2:**
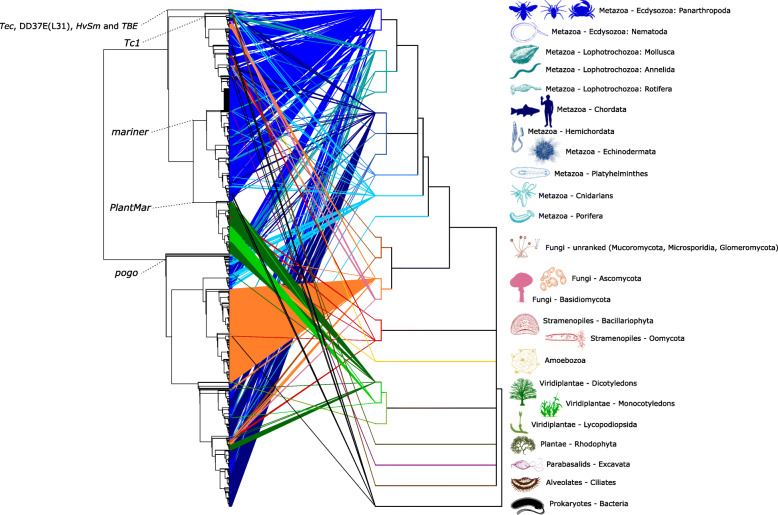
Tanglegram summarising the fit between *Tc1/mariner* phylogeny (on the left) and eukaryotic host phylogeny (on the right). Coloured silhouettes indicate the host group that corresponds to each branch of eukaryotic diversity, and lines of corresponding colour link each TE with its host group. Where a great number of elements link to a certain host group, the lines appear as a solid block

A tanglegram indicating links between *Tc1/mariner* phylogeny and host phylogeny at the level of eukaryote orders is presented in Fig. 2. The tanglegram illustrates a high level of incongruence, suggesting widespread horizontal transfer of *Tc1/mariner* TEs across host diversity. An alternative explanation for the observed pattern is that each *Tc1/mariner* family was present in the eukaryotic ancestral lineage, and subsequently active elements representing each *Tc1/mariner* family have been selectively retained in just some host lineages. However, this would require invoking a very large number of loss events across eukaryote phylogeny. Consequently, given the unlikeliness of the alternative hypothesis together with recent research demonstrating the frequency with which horizontal transmission can occur (see below), we suggest that a history of horizontal transfer is the most likely explanation for the observed host distribution of *Tc1/mariner* elements.

Over recent years, considerable evidence of the widespread horizontal transfer of TEs (HTTs) has become apparent [33, 59, 60]. Of particular relevance is a recent analysis of HTT among 195 insect genomes, which found that *Tc1/mariner* elements were the most frequently horizontally transmitted TE group [60]. Indeed, despite their highly conservative approach, the authors identified > 1000 putative horizontal transfer events involving *Tc1/mariner* elements among a relatively small sample of insect genomes [60], equating to approximately 5 HTTs per insect species considered. Moreover, *Tc1/mariner* elements were also found to occupy the highest mean fraction of the host genome among horizontally transferred TE groups [60], adding evidence to their propensity to transfer horizontally.

Confirming recent examples of HTT based on complete transposase sequences remains problematic. We identified 15 potential cases of recent HTTs in the *Tc1/mariner* superfamily, based on strong clade support values between two or more sequences present in the genomes of distantly related eukaryotes (Table 1). For example, in the *mariner* family, a sequence from the sheep *Ovis aries*, clusters with sequences from the ant *Oocera biroi*, and the bacterium *Pseudomonas monteilii* (Clade 32, Additional file 2). Ecologically, it is plausible to invoke a HTT event between these taxa given their shared environment. However, as we were unable to confirm the host genomic context of the transposases in question, we cannot rule out other explanations for the observed patterns (such as contamination during lab work or sequencing). In the *pogo* family, we identified a potential HTT between the blue tit (*Cyanistes caeruleus*) and the pepper (*Capsicum annuum*) (Clade 52, Additional file 2), and the lettuce (*Latuca sativa*) and the yellow sugar cane aphid (*Sipha flava*) (Clade 50, Additional file 2). In both cases, we searched for full-length elements in each genome to check flanking sequences (see Methods). However, once more, in both cases the element or partial element was located on a small contig, making verification impossible (Table 1). We were able to find a full-length element in just three cases of potential HTT, which involved the following host genomes: the White-Ruffed Manakin bird (*Corapipo altera*) and the squinting bush brown butterfly (*Bicyclus anynana*); the Queensland fruit fly (*Bactrocera tryoni*) and the bacterium *Desulfovibrio*; and, the Chinese tree shrew (*Tupaia chinensis*) and several hymenopterans (Table 1).

**Table 1:**
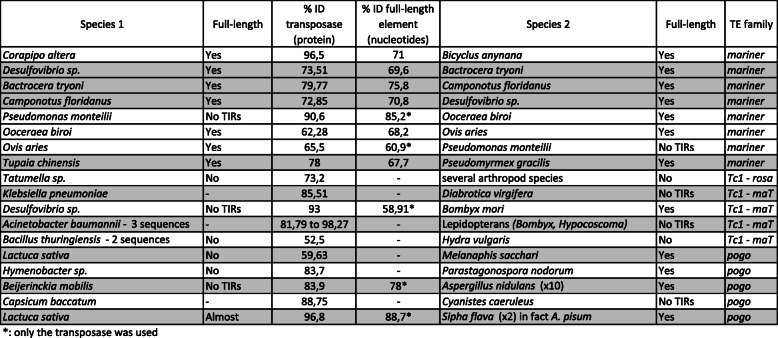
Potential cases of horizontal transfer and the shared amino acid and nucleotide identity of transposases

^*^only the transposase was used

For each case of potential HTT we identified, if a genome was available and we were able to identify a full transposase domain, we performed an NCBI BLASTn search of the host genome using the transposase domain of the element as the query. In five cases out of seven, this identified just one hit for the TE involved, suggesting that the sequence may result from contamination rather than represent a real transposon present in that host genome. In the two cases where > 1 copy of the transposase was found, the identity of each hit was > 95% in one genome, but < 95% in the other species, suggesting its presence in one species may be in doubt. Thus, no convincing cases of very recent HTT could be identified. As more high quality long-read genomes become available for interrogation, our ability to identify recent HTT events will increase, and the *Tc1/mariner* superfamily represents an excellent model for further study.

## Conclusions

*Tc1/mariner* is a widespread DNA TE superfamily that is especially common in fungal and animal hosts. Four major *Tc1/mariner* families dominate the superfamily (*Tc1, mariner, PlantMar, pogo*), while four minor families contain just a few sequences each (*Tec*, *HvSm*, DD37E(L31), *TBE*). The well-known *Tc1* and *mariner* families are well-supported, closely phylogenetically related, and found predominantly in invertebrate genomes. The *PlantMar* family is the sister-group to the *mariner* family, and host usage in this group indicates a strict switch from invertebrates to angiosperms. *Pogo* is by far the largest *Tc1/Mariner* family and displays the widest host distribution, with a large number of sequences from fungal and animal genomes, and a smaller number of sequences from plant, oomycete and amoeboid protist genomes (Figs. 1 and 2). We report several lines of evidence that suggest *pogo* may be more appropriately considered to be a separate DNA TE superfamily. Additionally, we find compelling evidence that *pogo* elements have been exapted by tetrapod genomes, and we provide evidence that support the molecular domestication of *TIGD1-TIGD7*.

Several questions arise from our study, relating to the evolution and host range of *Tc1/mariner* elements. A key question is what enables *Tc1/mariner* elements to exploit such a diverse variety of hosts? For example, does their typically short and simple structure assist in their propensity to persist in the genomes of diverse host organisms, or do they possess a currently unidentified mechanism that facilitates host generalism? Additionally, what processes explain the variability of TIRs in *Tc1/mariner* elements, and is this in some way related to their host-parasite dynamics? The extent to which *Tc1/mariner* elements have been domesticated by host genomes, especially *pogo* elements, also remains unclear. Further research is required to clarify potential host shifts among distantly related host taxa, and it remains to be determined whether *Tc1/mariner* elements occasionally invade bacterial genomes or if their apparent presence in several bacteria can be decisively attributed to contamination. Meanwhile, a taxonomic issue is whether the *pogo* family would be better elevated to superfamily status, given the differences that set it apart from other *Tc1/mariner* elements.

## Methods

### Mining and alignment of Tc1/mariner elements

We employed BLASTp queries of the NCBI *nr* database and a PSI-BLAST [61] of the swissprot database [62], using an in-house pipeline. Our query sequences were the *Tc1/mariner* DDD/E transposase domains provided in the supplementary material of Yuan & Wessler [4], and sequences for *Tc1/mariner* transposons described in specific relevant publications [1, 12, 22, 24–27, 35, 49, 63, 64], all in amino acid format and from seven *Tc1/mariner* families. We focus on amino acid data since amino acid sequences evolve more slowly than DNA sequence data, facilitating sequence alignment at deeper evolutionary timescales, such as those across an entire DNA TE superfamily. This is important not just for phylogenetic accuracy, but also for interpreting patterns in transposase structure, such as diagnostic features of the DDD/E motif.

A preliminary phylogeny was estimated using query sequences together with those downloaded from the database search, after which one sequence was used as an additional query for any newly identified clades. A pipeline involving a filtering step to selectively retain matches with a minimum of 50% identity over at least 50% of the length of the query sequence was utilised. We manually added sequences from the studies of Ray et al. (2008) for *Myotis lucifugus*, Dupeyron et al. (2014) for *Armadillidium vulgare*, Puzakov et al. (2018) for *Crassostrea gigas* [1, 35, 65], and sequences resulting from an independent DNA TEs annotation in *Myzus persicae nicotianae* (Toby Baril, unpublished data). In total, 5158 *Tc1/mariner* amino acid transposases were retrieved. Matches were extracted and processed into fasta format with the awk and sed EMBOSS tool *v6.6.0.0* [66]. These hits correspond to transposase domains, and they do not reflect copy number in the respective host genomes from which they were retrieved, neither do they provide any indication of the abundance of non-autonomous elements in these genomes.

To briefly check the copy number of *Tc1/mariner* elements in eukaryote genomes, we used reference *mariner* and *pogo* elements described in *Hydra vulgaris* (Mariner-16_HM and Mariner-18_HM, respectively) to perform BLASTn searches on the WGS data of this species in NCBI. Two Whole Genome Sequence (WGS) projects are available in NCBI and with a similar quality, so we selected only one of them (ACZU00000000) to avoid hit duplication. The hit tables were downloaded and we counted full-length, partial and MITE copies as follows: full-length copies were considered when a scaffold hit showed at least 95% identity over at least 95% of the length of the element, partial copies when the hit was at least 80% identity over at least 50% of the length of the element, non-autonomous copies when the hit was at least 50% identity over at least 25% of the element length, and MITEs were inferred when the hit was at least 95% identity over both the TIRs (according to hit coordinates). The summary of the copy numbers identified following this test can be found in Additional file 10. We assessed the number of amino acid residues between the second D and the third D/E in the transposase DDD/E motifs of each *Tc1/mariner* family, in our amino acid alignment in MEGA7 [67].

### Phylogenetic analyses

We focussed on the transposase domain, since this is a highly conserved region among DNA TEs and there is an established precedent for using this region for higher level phylogenetic analyses in DNA TEs [4]. Transposase domains were aligned using the DDE domain alignment of Yuan & Wessler [4] as a basis, and the profile alignment option of MUSCLE [68].

To infer the evolutionary history of the *Tc1/mariner* superfamily, we used FastTree *v2.1.11* [69], which applies minimum-evolution subtree-pruning-regrafting (SPRs) and maximum-likelihood nearest-neighbor interchanges (NNIs). We used the -spr 4 option to improve SPRs, the ‘–mlacc 2’ and ‘-slownni’ options to increase accuracy, and we performed 1000 bootstrap repetitions. We used members of the *IS630* TE group as an outgroup to root our phylogeny, since this group is considered most closely related to *Tc1/mariner* elements [4].

The tips of our tree were colour labelled by host taxon according to major taxonomic groupings in FigTree v1.4 (http://tree.bio.ed.ac.uk/software/figtree/). One element per superfamily and elements from newly identified groups were annotated with Artemis software [70], using the ORFfinder and BLASTx tools on NCBI for conserved domains, and the palindrome analyser tool of DNA Analyser to find TIRs [71]. Retrieval of classification information for the host species was computed with ETE3 [72] using in house python scripts. The tanglegram was produced using RStudio v3.5.1 Tydiverse [73] and ape v5.3 [74].

### Inferences of synteny

Conservation of the genomic location of transposases and neighbouring genes constitutes evidence of synteny. Considering the apparent conservation of three *pogo*-like elements in vertebrate organisms in subgroups 48, 49 and 53 (Additional file 2), we searched for their location and evidence of orthology in genomes available in Ensembl [54], using human sequences as a queries. We downloaded the tables provided by the orthology information contained on the website. According to the *Homo sapiens* genomic environment for each *pogo* sequence, we searched for orthology information for genes that were upstream and downstream of the transposase sequence. Summary tables were downloaded and manually checked, and the RStudio Tydiverse package was used to merge each table by the species column to provide a location for each *TIGD* element, and upstream and downstream genes.

### Study of potential horizontally transferred elements

Potential horizontal transfer events were studied as follows: firstly, the amino acid percentage identity of the DDE domain was calculated using stretcher from EMBOSS tools [66]. Then, we searched for the nucleotide sequence of the elements involved in NCBI and the percentage identity of the DNA transposase sequence was also calculated. In three cases, we found the full-length element in both species involved, and we calculated the percentage identity of the whole element between each pair (Table 1). Following these steps, we searched for the element with the transposase nucleotide sequence as a query in the genome of the species’ involved, if available in NCBI, to attempt to detect other copies. Only two pairs of species potentially involved in HTTs showed more than one hit for this search. However, the percentage identity was < 92% for these copies, and the overall percentage identity between the full-length elements in both species was < 80%, so we did not pursue this further.

## Supplementary information

**Additional file 1.** Taxonomic information for each eukaryote host represented in the phylogenetic tree

**Additional file 2. **Phylogenetic tree of the amino-acid DDD/E transposase domain of 5158 *Tc1/mariner* elements. The tree results from a phylogenetic analysis using maximum likelihood inference, with 1000 bootstrap repetitions. Clade support values above 70% are indicated adjacent to each clade. Clades are divided into groups, with a corresponding clade name and number to the right. Elements are named according to their Repbase or Genbank ID, or according to the name provided in the article describing them. The host genome for each element is indicated to the right hand side of its ID, and labels are coloured broadly according to the taxonomic kingdom and class that the host species belongs to: shades of blue for metazoans, purple for excavates (Parabasalids), dark red for oomycetes (Stramenopiles), yellow for amoebozoans, shades of orange for fungi (pink for Basidiomycota), and shades of green for plants.

**Additional file 3. **Alignment text files showing the DDD/E structure of each clade showing a conserved amino acid residues number between the second D and the third D or the E of the transposase domain. A) DDD/E alignment caption for *HvSm* and *PlantMar*. B) DDD alignment caption for *TIGD1–4*. C) DDD alignment caption for *TIGD*5–7.

**Additional file 4.** The amino acid alignment used to perform our phylogenetic analysis.

**Additional file 5. **Fasta sequences of the 75 full-length *Tc1/mariner* elements used in this study.

**Additional files 6 to 12.** Conserved locations of *TIGD1* to *TIGD7* in host vertebrate species and information about the upstream and downstream genes flanking them, retrieved from Ensembl [54]. Negative numbers indicate that the considered gene is upstream of the *TIGD* element.

**Additional File 13.** The amino acid alignment used to perform the *TIGD1–TIGD7* phylogenetic analysis.

**Additional File 14.** Phylogenetic tree of the amino-acid DDD/E transposase domain of *TIGD*-like elements. The tree results from a phylogenetic analysis using maximum likelihood inference, with 1000 bootstrap repetitions. Clades are divided into groups, with a corresponding clade name and number to the right. The host genome for each element is indicated to the right hand side of its ID, and labels are coloured broadly according to the taxonomic kingdom and class that the host species belongs to: shades of blue for metazoans, purple for excavates (Parabasalids), dark red for oomycetes (Stramenopiles), yellow for amoebozoans, shades of orange for fungi (pink for Basidiomycota), and shades of green for plants.

**Additional File 15.** Table showing the results of assessing the copy number of full-length *mariner* and *pogo* elements in the *Hydra vulgaris* genome.

## Data Availability

The data used in this study are available in Supplementary material.

## Abbreviations

TEs: Transposable elements
TIRs: Terminal inverted repeats
IESs: Internal eliminated sequences
TSD: Target site duplication
ITm: IS630-*Tc1/mariner*
CENP-B: Centromere protein B
ORF: Open reading frame
MITEs: Miniature inverted-repeat transposable elements
