# Supplementary material for: Phylogenetic analysis of the Tc1/mariner superfamily reveals the unexplored diversity of pogo-like elements
Source: Mob DNA. 2020 Jun 29;11:21. doi: 10.1186/s13100-020-00212-0 (PMC7325037; doi:10.1186/s13100-020-00212-0)
Supplement: Supplementary file 1 — Additional file 1. Taxonomic information for each eukaryote host represented in the phylogenetic tree [file 13100_2020_212_MOESM1_ESM.pdf]

Genus	species	sub-species	Kingdom	Phyla	Class	Order	Family	Number
Euplotes	*crassus*	-	Alveolata	Ciliophora	Hypotrichia	Euplotidae	*Tec*	4
Steinernema	*carpocapsae*	-	Metazoa	Nematoda	Chromadorea	Rhabditida	DD37E(L31)	1
Pinctada	*martensii*	-	Metazoa	Mollusca	Bivalvia	Pterioida	DD37E(L31)	1
Crassostrea	*virginica*	-	Metazoa	Mollusca	Bivalvia	Ostreoida	DD37E(L31)	1
Modiolus	*philippinarum*	-	Metazoa	Mollusca	Bivalvia	Mytiloida	DD37E(L31)	1
Mytilus	*galloprovencialis*	-	Metazoa	Mollusca	Bivalvia	Mytiloida	DD37E(L31)	1
Bathymodiolus	*platifrons*	-	Metazoa	Mollusca	Bivalvia	Mytiloida	DD37E(L31)	1
Exaiptasia	*pallida*	-	Metazoa	Cnidaria	Anthozoa	Actiniaria	DD37E(L31)	3
Mizuhopecten	*yessoensis*	-	Metazoa	Mollusca	Bivalvia	Pectinoida	DD37E(L31)	1
Acropora	*digitifera*	-	Metazoa	Cnidaria	Anthozoa	Scleractinia	DD37E(L31)	1
Schmidtea	*mediterranea*	-	Metazoa	Platyhelminthes	Rhabditophora	Tricladida	*HvSm*	1
Hydra	*vulgaris*	-	Metazoa	Cnidaria	Hydrozoa	Anthoathecata	*HvSm*	3
Oxytricha	*fallax*	-	Alveolata	Ciliophora	Stichotrichia	Oxytrichidae	*TBE*	13
Oxytricha	*trifallax*	-	Alveolata	Ciliophora	Stichotrichia	Oxytrichidae	*TBE*	3
Achipteria	*coleoptrata*	-	Metazoa	Arthropoda	Arachnida	Sarcoptiformes	*Tc1*	1
Achlya	*hypogyna*	-	Stramenopiles	-	Oomycetes	Saprolegniales	*Tc1*	1
Acinetobacter	*baumannii*	-	Bacteria	Proteobacteria	Gammaproteobacteria	Pseudomonadales	*Tc1*	3
Acipenser	*fulvescens*	-	Metazoa	Chordata	Actinopteri	Acipenseriformes	*Tc1*	1
Acromyrmex	*echinatior*	-	Metazoa	Arthropoda	Insecta	Hymenoptera	*Tc1*	1
Acyrthosiphon	*pisum*	-	Metazoa	Arthropoda	Insecta	Hemiptera	*Tc1*	1
Aedes	*albopictus*	-	Metazoa	Arthropoda	Insecta	Diptera	*Tc1*	1
Aedes	*atropalpus*	-	Metazoa	Arthropoda	Insecta	Diptera	*Tc1*	1
Albugo	*laibachii*	-	Stramenopiles	-	Oomycetes	Albuginales	*Tc1*	4
Amphiprion	*ocellaris*	-	Metazoa	Chordata	Actinopteri		*Tc1*	1
Amyelois	*transitella*	-	Metazoa	Arthropoda	Insecta	Lepidoptera	*Tc1*	1
Anabarilius	*grahami*	-	Metazoa	Chordata	Actinopteri	Cypriniformes	*Tc1*	1
Ancylostoma	*caninum*	-	Metazoa	Nematoda	Chromadorea	Strongylida	*Tc1*	13
Ancylostoma	*ceylanicum*	-	Metazoa	Nematoda	Chromadorea	Strongylida	*Tc1*	11
Ancylostoma	*duodenale*	-	Metazoa	Nematoda	Chromadorea	Strongylida	*Tc1*	9
Anopheles	*albimanus*	-	Metazoa	Arthropoda	Insecta	Diptera	*Tc1*	1
Anopheles	*gambiae*	-	Metazoa	Arthropoda	Insecta	Diptera	*Tc1*	5
Anoplophora	*glabripennis*	-	Metazoa	Arthropoda	Insecta	Coleoptera	*Tc1*	7
Apareiodon	*affinis*	-	Metazoa	Chordata	Actinopteri	Characiformes	*Tc1*	1
Apareiodon	*vittatus*	-	Metazoa	Chordata	Actinopteri	Characiformes	*Tc1*	2
Apis	*mellifera*	-	Metazoa	Arthropoda	Insecta	Hymenoptera	*Tc1*	7
Apostichopus	*parvimensis*	-	Metazoa	Echinodermata	Holothuroidea	Aspidochirotida	*Tc1*	1
Armadillidium	*vulgare*	-	Metazoa	Arthropoda	Malacostraca	Isopoda	*Tc1*	5
Aspergillus	*nidulans*	-	Fungi	Ascomycota	Eurotiomycetes	Eurotiales	*Tc1*	1
Aspergillus	*niger*	-	Fungi	Ascomycota	Eurotiomycetes	Eurotiales	*Tc1*	2
Aspergillus	*oryzae*	-	Fungi	Ascomycota	Eurotiomycetes	Eurotiales	*Tc1*	2
Atta	*cephalotes*	-	Metazoa	Arthropoda	Insecta	Hymenoptera	*Tc1*	2
Atta	*colombica*	-	Metazoa	Arthropoda	Insecta	Hymenoptera	*Tc1*	4
Bacillus	*thuringiensis*	-	Bacteria	Firmicutes	Bacilli	Bacillales	*Tc1*	3
Bactrocera	*tryoni*	-	Metazoa	Arthropoda	Insecta	Diptera	*Tc1*	5
Blattella	*germanica*	-	Metazoa	Arthropoda	Insecta	Blattodea	*Tc1*	6
Bombyx	*mandarina*	-	Metazoa	Arthropoda	Insecta	Lepidoptera	*Tc1*	4
Bombyx	*mori*	-	Metazoa	Arthropoda	Insecta	Lepidoptera	*Tc1*	5
Caenorhabditis	*brenneri*	-	Metazoa	Nematoda	Chromadorea	Rhabditida	*Tc1*	2
Caenorhabditis	*briggsae*	-	Metazoa	Nematoda	Chromadorea	Rhabditida	*Tc1*	10
Caenorhabditis	*elegans*	-	Metazoa	Nematoda	Chromadorea	Rhabditida	*Tc1*	21
Caenorhabditis	*nigoni*	-	Metazoa	Nematoda	Chromadorea	Rhabditida	*Tc1*	33
Caenorhabditis	*remanei*	-	Metazoa	Nematoda	Chromadorea	Rhabditida	*Tc1*	18
Camponotus	*floridanus*	-	Metazoa	Arthropoda	Insecta	Hymenoptera	*Tc1*	12
Ceratitis	*rosa*	-	Metazoa	Arthropoda	Insecta	Diptera	*Tc1*	2
Chilo	*suppressalis*	-	Metazoa	Arthropoda	Insecta	Lepidoptera	*Tc1*	7
Chondrus	*crispus*	-	Plantae	Rhodophyta	Florideophyceae	Gigartinales	*Tc1*	8
Cokeromyces	*recurvatus*	-	Fungi	Mucoromycota	Mucoromycetes	Mucorales	*Tc1*	1
Cordyceps	*confragosa*	-	Fungi	Ascomycota	Sordariomycetes	Hypocreales	*Tc1*	1
Crassostrea	*gigas*	-	Metazoa	Mollusca	Bivalvia	Ostreoida	*Tc1*	22
Crassostrea	*virginica*	-	Metazoa	Mollusca	Bivalvia	Ostreoida	*Tc1*	1
Crotalus	*pyrrhus*	-	Metazoa	Chordata		Squamata	*Tc1*	1
Cryptotermes	*secundus*	-	Metazoa	Arthropoda	Insecta	Blattodea	*Tc1*	7
Ctenocephalides	*felis*	-	Metazoa	Arthropoda	Insecta	Siphonaptera	*Tc1*	1
Cunninghamella	*bertholletiae*	-	Fungi	Mucoromycota	Mucoromycetes	Mucorales	*Tc1*	1
Danio	*rerio*	-	Metazoa	Chordata	Actinopteri	Cypriniformes	*Tc1*	1
Desulfovibrio	*sp.*	-	Bacteria	Proteobacteria	Deltaproteobacteria	Desulfovibrionales	*Tc1*	1
Diabrotica	*virgifera*	-	Metazoa	Arthropoda	Insecta	Coleoptera	*Tc1*	2
Dinothrombium	*tinctorium*	-	Metazoa	Arthropoda	Arachnida	Trombidiformes	*Tc1*	2
Drosophila	*ananassae*	-	Metazoa	Arthropoda	Insecta	Diptera	*Tc1*	1
Drosophila	*hydei*	-	Metazoa	Arthropoda	Insecta	Diptera	*Tc1*	3
Drosophila	*melanogaster*	-	Metazoa	Arthropoda	Insecta	Diptera	*Tc1*	2
Drosophila	*virilis*	-	Metazoa	Arthropoda	Insecta	Diptera	*Tc1*	1
Drosophila	*yakuba*	-	Metazoa	Arthropoda	Insecta	Diptera	*Tc1*	6
Dufourea	*novaeangliae*	-	Metazoa	Arthropoda	Insecta	Hymenoptera	*Tc1*	8
Eurytemora	*affinis*	-	Metazoa	Arthropoda	Hexanauplia	Calanoida	*Tc1*	1
Fasciola	*hepatica*	-	Metazoa	Platyhelminthes	Trematoda	Plagiorchiida	*Tc1*	1
Folsomia	*candida*	-	Metazoa	Arthropoda	Collembola	Entomobryomorpha	*Tc1*	10
Fundulus	*heteroclitus*	-	Metazoa	Chordata	Actinopteri	Cyprinodontiformes	*Tc1*	1
Fusarium	*fujikuroi*	-	Fungi	Ascomycota	Sordariomycetes	Hypocreales	*Tc1*	2

Fusarium	*oxysporum*	-	Fungi	Ascomycota	Sordariomycetes	Hypocreales	*Tc1*	19
Gadus	*morhua*	-	Metazoa	Chordata	Actinopteri	Gadiformes	*Tc1*	2
Galleria	*mellonella*	-	Metazoa	Arthropoda	Insecta	Lepidoptera	*Tc1*	2
Ganoderma	*sinense*	-	Fungi	Basidiomycota	Agaricomycetes	Polyporales	*Tc1*	1
Gasterosteus	*aculeatus*	-	Metazoa	Chordata	Actinopteri	Perciformes	*Tc1*	4
Habropoda	*laboriosa*	-	Metazoa	Arthropoda	Insecta	Hymenoptera	*Tc1*	1
Haemonchus	*contortus*	-	Metazoa	Nematoda	Chromadorea	Strongylida	*Tc1*	19
Haemonchus	*placei*	-	Metazoa	Nematoda	Chromadorea	Strongylida	*Tc1*	8
Halyomorpha	*halys*	-	Metazoa	Arthropoda	Insecta	Hemiptera	*Tc1*	1
Haplochromis	*burtoni*	-	Metazoa	Chordata	Actinopteri	Cichliformes	*Tc1*	1
Harpegnathos	*saltator*	-	Metazoa	Arthropoda	Insecta	Hymenoptera	*Tc1*	28
Helicoverpa	*armigera*	-	Metazoa	Arthropoda	Insecta	Lepidoptera	*Tc1*	1
Heligmosomoides	*polygyrus*	-	Metazoa	Nematoda	Chromadorea	Strongylida	*Tc1*	8
Heliothis	*virescens*	-	Metazoa	Arthropoda	Insecta	Lepidoptera	*Tc1*	1
Hydra	*vulgaris*	-	Metazoa	Cnidaria	Hydrozoa	Anthoathecata	*Tc1*	6
Hyposmocoma	*kahamanoa*	-	Metazoa	Arthropoda	Insecta	Lepidoptera	*Tc1*	5
Hypothenemus	*hampei*	-	Metazoa	Arthropoda	Insecta	Coleoptera	*Tc1*	21
Klebsiella	*pneumoniae*	-	Bacteria	Proteobacteria	Gammaproteobacteria	Enterobacterales	*Tc1*	1
Lasius	*niger*	-	Metazoa	Arthropoda	Insecta	Hymenoptera	*Tc1*	8
Lepeophtheirus	*salmonis*	-	Metazoa	Arthropoda	Hexanauplia	Siphonostomatoida	*Tc1*	3
Leptinotarsa	*decemlineata*	-	Metazoa	Arthropoda	Insecta	Coleoptera	*Tc1*	2
Lichtheimia	*corymbifera*	-	Fungi	Mucoromycota	Mucoromycetes	Mucorales	*Tc1*	1
Linepithema	*humile*	-	Metazoa	Arthropoda	Insecta	Hymenoptera	*Tc1*	1
Litomosoides	*sigmodontis*	-	Metazoa	Nematoda	Chromadorea	Rhabditida	*Tc1*	1
Lucilia	*cuprina*	-	Metazoa	Arthropoda	Insecta	Diptera	*Tc1*	2
Misgolas	*hubbardi*	-	Metazoa	Arthropoda	Arachnida	Araneae	*Tc1*	1
Mucor	*circinelloides*	-	Fungi	Mucoromycota	Mucoromycetes	Mucorales	*Tc1*	1
Mucor	*racemosus*	-	Fungi	Mucoromycota	Mucoromycetes	Mucorales	*Tc1*	1
Mucor	*ramosissimus*	-	Fungi	Mucoromycota	Mucoromycetes	Mucorales	*Tc1*	1
Mucor	*velutinosus*	-	Fungi	Mucoromycota	Mucoromycetes	Mucorales	*Tc1*	1
Musca	*domestica*	-	Metazoa	Arthropoda	Insecta	Diptera	*Tc1*	1
Myotis	*lucifugus*	-	Metazoa	Chordata	Mammalia	Chiroptera	*Tc1*	9
Myzus	*persicae*	*nicotianae*	Metazoa	Arthropoda	Insecta	Hemiptera	*Tc1*	30
Necator	*americanus*	-	Metazoa	Nematoda	Chromadorea	Strongylida	*Tc1*	1
Nematostella	*vectensis*	-	Metazoa	Cnidaria	Anthozoa	Actiniaria	*Tc1*	1
Nilaparvata	*lugens*	-	Metazoa	Arthropoda	Insecta	Hemiptera	*Tc1*	7
Nippostrongylus	*brasiliensis*	-	Metazoa	Nematoda	Chromadorea	Strongylida	*Tc1*	8
Nuttalliella	*namaqua*	-	Metazoa	Arthropoda	Arachnida	Ixodida	*Tc1*	1
Oesophagostomum	*dentatum*	-	Metazoa	Nematoda	Chromadorea	Strongylida	*Tc1*	13
Oncorhynchus	*mykiss*	-	Metazoa	Chordata	Actinopteri	Salmoniformes	*Tc1*	18
Onthophagus	*taurus*	-	Metazoa	Arthropoda	Insecta	Coleoptera	*Tc1*	4
Ooceraea	*biroi*	-	Metazoa	Arthropoda	Insecta	Hymenoptera	*Tc1*	1
Orbicella	*faveolata*	-	Metazoa	Cnidaria	Anthozoa	Scleractinia	*Tc1*	1
Oreochromis	*niloticus*	-	Metazoa	Chordata	Actinopteri	Cichliformes	*Tc1*	2
Oryzias	*latipes*	-	Metazoa	Chordata	Actinopteri	Beloniformes	*Tc1*	2
Ostrinia	*furnacalis*	-	Metazoa	Arthropoda	Insecta	Lepidoptera	*Tc1*	2
Papilio	*machaon*	-	Metazoa	Arthropoda	Insecta	Lepidoptera	*Tc1*	3
Papilio	*xuthus*	-	Metazoa	Arthropoda	Insecta	Lepidoptera	*Tc1*	2
Parhyale	*hawaiensis*	-	Metazoa	Arthropoda	Malacostraca	Amphipoda	*Tc1*	1
Parodon	*nasus*	-	Metazoa	Chordata	Actinopteri	Characiformes	*Tc1*	2
Penicillium	*arizonense*	-	Fungi	Ascomycota	Eurotiomycetes	Eurotiales	*Tc1*	1
Penicillium	*brasilianum*	-	Fungi	Ascomycota	Eurotiomycetes	Eurotiales	*Tc1*	3
Penicillium	*digitatum*	-	Fungi	Ascomycota	Eurotiomycetes	Eurotiales	*Tc1*	6
Penicillium	*nalgiovense*	-	Fungi	Ascomycota	Eurotiomycetes	Eurotiales	*Tc1*	2
Penicillium	*rubens*	-	Fungi	Ascomycota	Eurotiomycetes	Eurotiales	*Tc1*	1
Penicillium	*subrubescens*	-	Fungi	Ascomycota	Eurotiomycetes	Eurotiales	*Tc1*	1
Philodina	*roseola*	-	Metazoa	Rotifera	Bdelloidea	Philodinida	*Tc1*	1
Phytophthora	*megakarya*	-	Stramenopiles	-	Oomycetes	Peronosporales	*Tc1*	2
Phytophthora	*nicotianae*	-	Stramenopiles	-	Oomycetes	Peronosporales	*Tc1*	1
Phytophthora	*palmivora*	-	Stramenopiles	-	Oomycetes	Peronosporales	*Tc1*	5
Pieris	*rapae*	-	Metazoa	Arthropoda	Insecta	Lepidoptera	*Tc1*	2
Pleuronectes	*platessa*	-	Metazoa	Chordata	Actinopteri	Pleuronectiformes	*Tc1*	4
Pseudoloma	*neurophilia*	-	Fungi	Microsporidia			*Tc1*	1
Ramazzottius	*varieornatus*	-	Metazoa	Tardigrada	Eutardigrada	Parachela	*Tc1*	1
Rana	*pipiens*	-	Metazoa	Chordata	Amphibia	Anura	*Tc1*	1
Rediviva	*aurata*	-	Metazoa	Arthropoda	Insecta	Hymenoptera	*Tc1*	1
Rhagoletis	*zephyria*	-	Metazoa	Arthropoda	Insecta	Diptera	*Tc1*	1
Rhizophagus	*irregularis*	-	Fungi	Mucoromycota	Glomeromycetes	Glomerales	*Tc1*	2
Rhizopus	*delemar*	-	Fungi	Mucoromycota	Mucoromycetes	Mucorales	*Tc1*	1
Rhizopus	*microsporus*	-	Fungi	Mucoromycota	Mucoromycetes	Mucorales	*Tc1*	1
Rhizopus	*oryzae*	-	Fungi	Mucoromycota	Mucoromycetes	Mucorales	*Tc1*	1
Rhopalosiphum	*maidis*	-	Metazoa	Arthropoda	Insecta	Hemiptera	*Tc1*	3
Rhynchosporium	*agropyri*	-	Fungi	Ascomycota	Leotiomycetes	Helotiales	*Tc1*	2
Rhynchosporium	*commune*	-	Fungi	Ascomycota	Leotiomycetes	Helotiales	*Tc1*	1
Riptortus	*pedestris*	-	Metazoa	Arthropoda	Insecta	Hemiptera	*Tc1*	1
Salmo	*salar*	-	Metazoa	Chordata	Actinopteri	Salmoniformes	*Tc1*	3
Seriola	*dumerili*	-	Metazoa	Chordata	Actinopteri	Carangiformes	*Tc1*	1
Sparassis	*crispa*	-	Fungi	Basidiomycota	Agaricomycetes	Polyporales	*Tc1*	1
Spodoptera	*litura*	-	Metazoa	Arthropoda	Insecta	Lepidoptera	*Tc1*	1
Stehodyphus	*mimosarum*	-	Metazoa	Arthropoda	Arachnida	Araneae	*Tc1*	346

Stomoxys	*calcitrans*	-	Metazoa	Arthropoda	Insecta	Diptera	*Tc1*	4
Takifugu	*rubripes*	-	Metazoa	Chordata	Actinopteri	Tetraodontiformes	*Tc1*	3
Talaromyces	*marneffei*	-	Fungi	Ascomycota	Eurotiomycetes	Eurotiales	*Tc1*	4
Talaromyces	*stipitatus*	-	Fungi	Ascomycota	Eurotiomycetes	Eurotiales	*Tc1*	5
Teladorsagia	*circumcincta*	-	Metazoa	Nematoda	Chromadorea	Strongylida	*Tc1*	4
Tetraodon	*nigroviridis*	-	Metazoa	Chordata	Actinopteri	Tetraodontiformes	*Tc1*	2
Trachymyrmex	*cornetzi*	-	Metazoa	Arthropoda	Insecta	Hymenoptera	*Tc1*	2
Trachymyrmex	*septentrionalis*	-	Metazoa	Arthropoda	Insecta	Hymenoptera	*Tc1*	9
Tribolium	*castaneum*	-	Metazoa	Arthropoda	Insecta	Coleoptera	*Tc1*	4
Vanessa	*tameamea*	-	Metazoa	Arthropoda	Insecta	Lepidoptera	*Tc1*	2
Vollenhovia	*emeryi*	-	Metazoa	Arthropoda	Insecta	Hymenoptera	*Tc1*	4
Xenopus	*tropicalis*	-	Metazoa	Chordata	Amphibia	Anura	*Tc1*	3
Aegilops	*tauschii*	-	Viridiplantae	Streptophyta	Liliopsida	Poales	*plantmar*	25
Aphanomyces	*astaci*	-	Stramenopiles	-	Oomycetes	Saprolegniales	*plantmar*	8
Arabidopsis	*lyrata*	-	Viridiplantae	Streptophyta	Eudicotyledons	Brassicales	*plantmar*	2
Arundinaria	*fargesii*	-	Viridiplantae	Streptophyta	Liliopsida	Poales	*plantmar*	1
Bambusa	*bambos*	-	Viridiplantae	Streptophyta	Liliopsida	Poales	*plantmar*	1
Bambusa	*emeiensis*	-	Viridiplantae	Streptophyta	Liliopsida	Poales	*plantmar*	1
Bambusa	*multiplex*	-	Viridiplantae	Streptophyta	Liliopsida	Poales	*plantmar*	1
Beta	*vulgaris*	-	Viridiplantae	Streptophyta	Eudicotyledons	Caryophyllales	*plantmar*	42
Brachypodium	*distachyon*	-	Viridiplantae	Streptophyta	Liliopsida	Poales	*plantmar*	8
Brassica	*napus*	-	Viridiplantae	Streptophyta	Eudicotyledons	Brassicales	*plantmar*	6
Brassica	*oleracea*	-	Viridiplantae	Streptophyta	Eudicotyledons	Brassicales	*plantmar*	6
Brassica	*rapa*	-	Viridiplantae	Streptophyta	Eudicotyledons	Brassicales	*plantmar*	5
Camelina	*sativa*	-	Viridiplantae	Streptophyta	Eudicotyledons	Brassicales	*plantmar*	22
Camellia	*sinensis*	-	Viridiplantae	Streptophyta	Eudicotyledons	Ericales	*plantmar*	3
Capsicum	*annuum*	-	Viridiplantae	Streptophyta	Eudicotyledons	Solanales	*plantmar*	9
Cephalostachyum	*pergracile*	-	Viridiplantae	Streptophyta	Liliopsida	Poales	*plantmar*	1
Chenopodium	*quinoa*	-	Viridiplantae	Streptophyta	Eudicotyledons	Caryophyllales	*plantmar*	11
Chimonobambusa	*marmorea*	-	Viridiplantae	Streptophyta	Liliopsida	Poales	*plantmar*	1
Chimonocalamus	*pallens*	-	Viridiplantae	Streptophyta	Liliopsida	Poales	*plantmar*	1
Coffea	*arabica*	-	Viridiplantae	Streptophyta	Eudicotyledons	Gentianales	*plantmar*	18
Coffea	*eugenioides*	-	Viridiplantae	Streptophyta	Eudicotyledons	Gentianales	*plantmar*	15
Corchorus	*capsularis*	-	Viridiplantae	Streptophyta	Eudicotyledons	Malvales	*plantmar*	3
Corchorus	*olitorius*	-	Viridiplantae	Streptophyta	Eudicotyledons	Malvales	*plantmar*	12
Cuscuta	*australis*	-	Viridiplantae	Streptophyta	Eudicotyledons	Solanales	*plantmar*	1
Cynodon	*dactylon*	-	Viridiplantae	Streptophyta	Liliopsida	Poales	*plantmar*	1
Daucus	*carota*	-	Viridiplantae	Streptophyta	Eudicotyledons	Apiales	*plantmar*	21
Dendrocalamus	*minor*	-	Viridiplantae	Streptophyta	Liliopsida	Poales	*plantmar*	1
Erythranthe	*guttata*	-	Viridiplantae	Streptophyta	Eudicotyledons	Lamiales	*plantmar*	5
Eutrema	*salsugineum*	-	Viridiplantae	Streptophyta	Eudicotyledons	Brassicales	*plantmar*	1
Fargesia	*fungosa*	-	Viridiplantae	Streptophyta	Liliopsida	Poales	*plantmar*	1
Fistulifera	*solaris*	-	Stramenopiles	Bacillariophyta	Bacillariophyceae	Naviculales	*plantmar*	1
Gelidocalamus	*annulatus*	-	Viridiplantae	Streptophyta	Liliopsida	Poales	*plantmar*	1
Glycine	*max*	-	Viridiplantae	Streptophyta	Eudicotyledons	Fabales	*plantmar*	8
Glycine	*soja*	-	Viridiplantae	Streptophyta	Eudicotyledons	Fabales	*plantmar*	5
Helianthus	*annuus*	-	Viridiplantae	Streptophyta	Eudicotyledons	Asterales	*plantmar*	4
Hevea	*brasiliensis*	-	Viridiplantae	Streptophyta	Eudicotyledons	Malpighiales	*plantmar*	1
Hibanobambusa	*tranquillans*	-	Viridiplantae	Streptophyta	Liliopsida	Poales	*plantmar*	1
Himalayacalamus	*intermedius*	-	Viridiplantae	Streptophyta	Liliopsida	Poales	*plantmar*	1
Hordeum	*vulgare*	-	Viridiplantae	Streptophyta	Liliopsida	Poales	*plantmar*	1
Ipomoea	*nil*	-	Viridiplantae	Streptophyta	Eudicotyledons	Solanales	*plantmar*	3
Jatropha	*curcas*	-	Viridiplantae	Streptophyta	Eudicotyledons	Malpighiales	*plantmar*	1
Lactuca	*sativa*	-	Viridiplantae	Streptophyta	Eudicotyledons	Asterales	*plantmar*	12
Medicago	*truncatula*	-	Viridiplantae	Streptophyta	Eudicotyledons	Fabales	*plantmar*	23
Melocalamus	*arrectus*	-	Viridiplantae	Streptophyta	Liliopsida	Poales	*plantmar*	1
Melocanna	*baccifera*	-	Viridiplantae	Streptophyta	Liliopsida	Poales	*plantmar*	1
Nicotiana	*attenuata*	-	Viridiplantae	Streptophyta	Eudicotyledons	Solanales	*plantmar*	1
Nicotiana	*tabacum*	-	Viridiplantae	Streptophyta	Eudicotyledons	Solanales	*plantmar*	2
Nicotiana	*tomentosiformis*	-	Viridiplantae	Streptophyta	Eudicotyledons	Solanales	*plantmar*	1
Oligostachyum	*sulcatum*	-	Viridiplantae	Streptophyta	Liliopsida	Poales	*plantmar*	1
Oryza	*brachyantha*	-	Viridiplantae	Streptophyta	Liliopsida	Poales	*plantmar*	4
Oryza	*rufipogon*	-	Viridiplantae	Streptophyta	Liliopsida	Poales	*plantmar*	1
Oryza	*sativa*	-	Viridiplantae	Streptophyta	Liliopsida	Poales	*plantmar*	45
Otatea	*acuminata*	-	Viridiplantae	Streptophyta	Liliopsida	Poales	*plantmar*	1
Panicum	*hallii*	-	Viridiplantae	Streptophyta	Liliopsida	Poales	*plantmar*	5
Panicum	*miliaceum*	-	Viridiplantae	Streptophyta	Liliopsida	Poales	*plantmar*	4
Papaver	*somniferum*	-	Viridiplantae	Streptophyta	Dicotyledons	Ranunculales	*plantmar*	26
Phyllostachys	*edulis*	-	Viridiplantae	Streptophyta	Liliopsida	Poales	*plantmar*	19
Pisum	*sativum*	-	Viridiplantae	Streptophyta	Eudicotyledons	Fabales	*plantmar*	4
Plasmopara	*halstedii*	-	Stramenopiles	-	Oomycetes	Peronosporales	*plantmar*	1
Pleioblastus	*fortunei*	-	Viridiplantae	Streptophyta	Liliopsida	Poales	*plantmar*	1
Pleioblastus	*gramineus*	-	Viridiplantae	Streptophyta	Liliopsida	Poales	*plantmar*	1
Pseudo-nitzschia	*multiseries*	-	Stramenopiles	Bacillariophyta	Bacillariophyceae	Bacillariales	*plantmar*	2
Pseudosasa	*japonica*	-	Viridiplantae	Streptophyta	Liliopsida	Poales	*plantmar*	1
Pseudostachyum	*polymorphum*	-	Viridiplantae	Streptophyta	Liliopsida	Poales	*plantmar*	1
Raphanus	*sativus*	-	Viridiplantae	Streptophyta	Eudicotyledons	Brassicales	*plantmar*	1
Sasa	*veitchii*	-	Viridiplantae	Streptophyta	Liliopsida	Poales	*plantmar*	1
Schizostachyum	*funghomii*	-	Viridiplantae	Streptophyta	Liliopsida	Poales	*plantmar*	1
Semiarundinaria	*densiflora*	-	Viridiplantae	Streptophyta	Liliopsida	Poales	*plantmar*	1

Setaria	*italica*	-	Viridiplantae	Streptophyta	Liliopsida	Poales	*plantmar*	9
Shibataea	*chinensis*	-	Viridiplantae	Streptophyta	Liliopsida	Poales	*plantmar*	1
Solanum	*tuberosum*	-	Viridiplantae	Streptophyta	Eudicotyledons	Solanales	*plantmar*	10
Sorghum	*bicolor*	-	Viridiplantae	Streptophyta	Liliopsida	Poales	*plantmar*	4
Spinacia	*oleracea*	-	Viridiplantae	Streptophyta	Eudicotyledons	Caryophyllales	*plantmar*	85
Thyrsostachys	*oliveri*	-	Viridiplantae	Streptophyta	Liliopsida	Poales	*plantmar*	1
Trifolium	*pratense*	-	Viridiplantae	Streptophyta	Eudicotyledons	Fabales	*plantmar*	2
Tripsacum	*pilosum*	-	Viridiplantae	Streptophyta	Liliopsida	Poales	*plantmar*	1
Zea	*diploperennis*	-	Viridiplantae	Streptophyta	Liliopsida	Poales	*plantmar*	2
Zea	*mays*	-	Viridiplantae	Streptophyta	Liliopsida	Poales	*plantmar*	6
Acromyrmex	*echinatior*	-	Metazoa	Arthropoda	Insecta	Hymenoptera	*mariner*	7
Adineta	*vaga*	-	Metazoa	Rotifera	Bdelloidea	Adinetida	*mariner*	20
Aethina	*tumida*	-	Metazoa	Arthropoda	Insecta	Coleoptera	*mariner*	1
Agrilus	*planipennis*	-	Metazoa	Arthropoda	Insecta	Coleoptera	*mariner*	2
Alvinella	*caudata*	-	Metazoa	Annelida	Polychaeta	Terebellida	*mariner*	1
Amegilla	*dulcifera*	-	Metazoa	Arthropoda	Insecta	Hymenoptera	*mariner*	2
Amyelois	*transitella*	-	Metazoa	Arthropoda	Insecta	Lepidoptera	*mariner*	2
Ancylostoma	*caninum*	-	Metazoa	Nematoda	Chromadorea	Strongylida	*mariner*	1
Ancylostoma	*ceylanicum*	-	Metazoa	Nematoda	Chromadorea	Strongylida	*mariner*	17
Andrena	*erigeniae*	-	Metazoa	Arthropoda	Insecta	Hymenoptera	*mariner*	2
Anopheles	*gambiae*	-	Metazoa	Arthropoda	Insecta	Diptera	*mariner*	2
Aphis	*glycines*	-	Metazoa	Arthropoda	Insecta	Hemiptera	*mariner*	1
Aphis	*gossypii*	-	Metazoa	Arthropoda	Insecta	Hemiptera	*mariner*	1
Apis	*cerana*	-	Metazoa	Arthropoda	Insecta	Hymenoptera	*mariner*	2
Apis	*mellifera*	-	Metazoa	Arthropoda	Insecta	Hymenoptera	*mariner*	5
Atta	*cephalotes*	-	Metazoa	Arthropoda	Insecta	Hymenoptera	*mariner*	6
Atta	*colombica*	-	Metazoa	Arthropoda	Insecta	Hymenoptera	*mariner*	3
Attacus	*atlas*	-	Metazoa	Arthropoda	Insecta	Lepidoptera	*mariner*	1
Bactrocera	*tryoni*	-	Metazoa	Arthropoda	Insecta	Diptera	*mariner*	7
Bicyclus	*anynana*	-	Metazoa	Arthropoda	Insecta	Lepidoptera	*mariner*	1
Bittacus	*strigosus*	-	Metazoa	Arthropoda	Insecta	Mecoptera	*mariner*	1
Bombus	*terrestris*	-	Metazoa	Arthropoda	Insecta	Hymenoptera	*mariner*	1
Bombyx	*mori*	-	Metazoa	Arthropoda	Insecta	Lepidoptera	*mariner*	2
Buenoa	*sp.*	-	Metazoa	Arthropoda	Insecta	Hemiptera	*mariner*	1
Bythograea	*thermydron*	-	Metazoa	Arthropoda	Malacostraca	Decapoda	*mariner*	5
Caenorhabditis	*brenneri*	-	Metazoa	Nematoda	Chromadorea	Rhabditida	*mariner*	5
Caenorhabditis	*briggsae*	-	Metazoa	Nematoda	Chromadorea	Rhabditida	*mariner*	1
Caenorhabditis	*elegans*	-	Metazoa	Nematoda	Chromadorea	Rhabditida	*mariner*	21
Caenorhabditis	*nigoni*	-	Metazoa	Nematoda	Chromadorea	Rhabditida	*mariner*	70
Caenorhabditis	*remanei*	-	Metazoa	Nematoda	Chromadorea	Rhabditida	*mariner*	13
Camponotus	*floridanus*	-	Metazoa	Arthropoda	Insecta	Hymenoptera	*mariner*	41
Campsomeris	*sp.*	-	Metazoa	Arthropoda	Insecta	Hymenoptera	*mariner*	1
Centruroides	*sculpturatus*	-	Metazoa	Arthropoda	Arachnida	Scorpiones	*mariner*	15
Ceratina	*calcarata*	-	Metazoa	Arthropoda	Insecta	Hymenoptera	*mariner*	1
Ceratitis	*capitata*	-	Metazoa	Arthropoda	Insecta	Diptera	*mariner*	3
Cerotoma	*trifurcata*	-	Metazoa	Arthropoda	Insecta	Coleoptera	*mariner*	1
Cheiroseius	*sp.*	-	Metazoa	Arthropoda	Arachnida	Mesostigmata	*mariner*	1
Chilo	*suppressalis*	-	Metazoa	Arthropoda	Insecta	Lepidoptera	*mariner*	1
Chrysoperla	*plorabunda*	-	Metazoa	Arthropoda	Insecta	Neuroptera	*mariner*	5
Chrysops	*vittatus*	-	Metazoa	Arthropoda	Insecta	Diptera	*mariner*	1
Chymomyza	*amoena*	-	Metazoa	Arthropoda	Insecta	Diptera	*mariner*	1
Corapipo	*altera*	-	Metazoa	Chordata	Aves	Passeriformes	*mariner*	1
Crassostrea	*gigas*	-	Metazoa	Mollusca	Bivalvia	Ostreoida	*mariner*	4
Cryptotermes	*secundus*	-	Metazoa	Arthropoda	Insecta	Blattodea	*mariner*	6
Ctenocephalides	*felis*	-	Metazoa	Arthropoda	Insecta	Siphonaptera	*mariner*	10
Ctenolepisma	*lineata*	-	Metazoa	Arthropoda	Insecta	Zygentoma	*mariner*	3
Culex	*restuans*	-	Metazoa	Arthropoda	Insecta	Diptera	*mariner*	1
Cyphomyrmex	*costatus*	-	Metazoa	Arthropoda	Insecta	Hymenoptera	*mariner*	2
Cyphononyx	*dorsalis*	-	Metazoa	Arthropoda	Insecta	Hymenoptera	*mariner*	3
Dermatophagoides	*pteronyssinus*	-	Metazoa	Arthropoda	Arachnida	Sarcoptiformes	*mariner*	2
Desulfovibrio	*sp.*	-	Bacteria	Proteobacteria	Deltaproteobacteria	Desulfovibrionales	*mariner*	1
Dinoponera	*quadriceps*	-	Metazoa	Arthropoda	Insecta	Hymenoptera	*mariner*	3
Diploscapter	*pachys*	-	Metazoa	Nematoda	Chromadorea	Rhabditida	*mariner*	1
Diuraphis	*noxia*	-	Metazoa	Arthropoda	Insecta	Hemiptera	*mariner*	5
Drosophila	*elegans*	-	Metazoa	Arthropoda	Insecta	Diptera	*mariner*	1
Drosophila	*ficusphila*	-	Metazoa	Arthropoda	Insecta	Diptera	*mariner*	1
Drosophila	*mauritiana*	-	Metazoa	Arthropoda	Insecta	Diptera	*mariner*	9
Drosophila	*melanogaster*	-	Metazoa	Arthropoda	Insecta	Diptera	*mariner*	2
Drosophila	*nikananu*	-	Metazoa	Arthropoda	Insecta	Diptera	*mariner*	2
Drosophila	*sechellia*	-	Metazoa	Arthropoda	Insecta	Diptera	*mariner*	2
Drosophila	*simulans*	-	Metazoa	Arthropoda	Insecta	Diptera	*mariner*	7
Drosophila	*takahashii*	-	Metazoa	Arthropoda	Insecta	Diptera	*mariner*	1
Drosophila	*teissieri*	-	Metazoa	Arthropoda	Insecta	Diptera	*mariner*	2
Dufourea	*novaeangliae*	-	Metazoa	Arthropoda	Insecta	Hymenoptera	*mariner*	14
Elysia	*chlorotica*	-	Metazoa	Mollusca	Gastropoda	-	*mariner*	12
Epicauta	*funebris*	-	Metazoa	Arthropoda	Insecta	Coleoptera	*mariner*	1
Eriphia	*verrucosa*	-	Metazoa	Arthropoda	Malacostraca	Decapoda	*mariner*	3
Folsomia	*candida*	-	Metazoa	Arthropoda	Collembola	Entomobryomorpha	*mariner*	1
Forficula	*auricularia*	-	Metazoa	Arthropoda	Insecta	Dermaptera	*mariner*	18
Galba	*truncatula*	-	Metazoa	Mollusca	Gastropoda	-	*mariner*	1

Galendromus	*occidentalis*	-	Metazoa	Arthropoda	Arachnida	Mesostigmata	*mariner*	2
Girardia	*tigrina*	-	Metazoa	Platyhelminthes	Rhabditophora	Tricladida	*mariner*	8
Glossina	*palpalis*	-	Metazoa	Arthropoda	Insecta	Diptera	*mariner*	1
Gnatocerus	*cornutus*	-	Metazoa	Arthropoda	Insecta	Coleoptera	*mariner*	1
Habropoda	*laboriosa*	-	Metazoa	Arthropoda	Insecta	Hymenoptera	*mariner*	3
Haematobia	*irritans*	-	Metazoa	Arthropoda	Insecta	Diptera	*mariner*	1
Haemonchus	*contortus*	-	Metazoa	Nematoda	Chromadorea	Strongylida	*mariner*	9
Haemonchus	*placei*	-	Metazoa	Nematoda	Chromadorea	Strongylida	*mariner*	1
Halyomorpha	*halys*	-	Metazoa	Arthropoda	Insecta	Hemiptera	*mariner*	11
Harpegnathos	*saltator*	-	Metazoa	Arthropoda	Insecta	Hymenoptera	*mariner*	1
Heliothis	*virescens*	-	Metazoa	Arthropoda	Insecta	Lepidoptera	*mariner*	4
Heteropoda	*venatoria*	-	Metazoa	Arthropoda	Arachnida	Araneae	*mariner*	2
Homo	*sapiens*	-	Metazoa	Chordata	Mammalia	Primates	*mariner*	4
Hydra	*littoralis*	-	Metazoa	Cnidaria	Hydrozoa	Anthoathecata	*mariner*	1
Hydra	*vulgaris*	-	Metazoa	Cnidaria	Hydrozoa	Anthoathecata	*mariner*	14
Hyposmocoma	*kahamanoa*	-	Metazoa	Arthropoda	Insecta	Lepidoptera	*mariner*	11
Lasius	*niger*	-	Metazoa	Arthropoda	Insecta	Hymenoptera	*mariner*	13
Linepithema	*humile*	-	Metazoa	Arthropoda	Insecta	Hymenoptera	*mariner*	1
Macrostomum	*lignano*	-	Metazoa	Platyhelminthes	Rhabditophora	Macrostomida	*mariner*	2
Mamestra	*brassicae*	-	Metazoa	Arthropoda	Insecta	Lepidoptera	*mariner*	1
Mantispa	*pulchella*	-	Metazoa	Arthropoda	Insecta	Neuroptera	*mariner*	2
Mayetiola	*destructor*	-	Metazoa	Arthropoda	Insecta	Diptera	*mariner*	4
Mayetiola	*hordei*	-	Metazoa	Arthropoda	Insecta	Diptera	*mariner*	3
Megachile	*rotundata*	-	Metazoa	Arthropoda	Insecta	Hymenoptera	*mariner*	2
Messor	*bouvieri*	-	Metazoa	Arthropoda	Insecta	Hymenoptera	*mariner*	4
Monomorium	*pharaonis*	-	Metazoa	Arthropoda	Insecta	Hymenoptera	*mariner*	1
Musca	*domestica*	-	Metazoa	Arthropoda	Insecta	Diptera	*mariner*	1
Myotis	*lucifugus*	-	Metazoa	Chordata	Mammalia	Chiroptera	*mariner*	16
Myzus	*persicae*	-	Metazoa	Arthropoda	Insecta	Hemiptera	*mariner*	1
Myzus	*persicae*	*nicotianae*	Metazoa	Arthropoda	Insecta	Hemiptera	*mariner*	241
Nilaparvata	*lugens*	-	Metazoa	Arthropoda	Insecta	Hemiptera	*mariner*	1
Oesophagostomum	*dentatum*	-	Metazoa	Nematoda	Chromadorea	Strongylida	*mariner*	1
Oncopeltus	*fasciatus*	-	Metazoa	Arthropoda	Insecta	Hemiptera	*mariner*	1
Ooceraea	*biroi*	-	Metazoa	Arthropoda	Insecta	Hymenoptera	*mariner*	11
Ophion	*sp.*	-	Metazoa	Arthropoda	Insecta	Hymenoptera	*mariner*	1
Oryctes	*borbonicus*	-	Metazoa	Arthropoda	Insecta	Coleoptera	*mariner*	1
Oscinella	*sp.*	-	Metazoa	Arthropoda	Insecta	Diptera	*mariner*	1
Ovis	*aries*	-	Metazoa	Chordata	Mammalia	Artiodactyla	*mariner*	1
Pachliopta	*aristolochiae*	-	Metazoa	Arthropoda	Insecta	Lepidoptera	*mariner*	1
Papilio	*machaon*	-	Metazoa	Arthropoda	Insecta	Lepidoptera	*mariner*	3
Papilio	*polytes*	-	Metazoa	Arthropoda	Insecta	Lepidoptera	*mariner*	1
Papilio	*xuthus*	-	Metazoa	Arthropoda	Insecta	Lepidoptera	*mariner*	1
Parambassis	*ranga*	-	Metazoa	Chordata	Actinopteri	-	*mariner*	1
Parasteatoda	*tepidariorum*	-	Metazoa	Arthropoda	Arachnida	Araneae	*mariner*	1
Phlebotomus	*papatasi*	-	Metazoa	Arthropoda	Insecta	Diptera	*mariner*	1
Pieris	*rapae*	-	Metazoa	Arthropoda	Insecta	Lepidoptera	*mariner*	1
Pilumnus	*hirtellus*	-	Metazoa	Arthropoda	Malacostraca	Decapoda	*mariner*	2
Pleistodontes	*addicotti*	-	Metazoa	Arthropoda	Insecta	Hymenoptera	*mariner*	1
Pleistodontes	*regalis*	-	Metazoa	Arthropoda	Insecta	Hymenoptera	*mariner*	1
Podisus	*maculiventris*	-	Metazoa	Arthropoda	Insecta	Hemiptera	*mariner*	1
Pogonomyrmex	*barbatus*	-	Metazoa	Arthropoda	Insecta	Hymenoptera	*mariner*	2
Polistes	*canadensis*	-	Metazoa	Arthropoda	Insecta	Hymenoptera	*mariner*	1
Polistes	*dominula*	-	Metazoa	Arthropoda	Insecta	Hymenoptera	*mariner*	4
Polistes	*formosanus*	-	Metazoa	Arthropoda	Insecta	Hymenoptera	*mariner*	2
Portunus	*pelagicus*	-	Metazoa	Arthropoda	Malacostraca	Decapoda	*mariner*	3
Pseudomonas	*monteilii*	-	Bacteria	Proteobacteria	Gammaproteobacteria	Pseudomonadales	*mariner*	1
Pseudomyrmex	*gracilis*	-	Metazoa	Arthropoda	Insecta	Hymenoptera	*mariner*	4
Rhagoletis	*zephyria*	-	Metazoa	Arthropoda	Insecta	Diptera	*mariner*	3
Schmidtea	*mediterranea*	-	Metazoa	Platyhelminthes	Rhabditophora	Tricladida	*mariner*	1
Sitodiplosis	*mosellana*	-	Metazoa	Arthropoda	Insecta	Diptera	*mariner*	2
Solenopsis	*invicta*	-	Metazoa	Arthropoda	Insecta	Hymenoptera	*mariner*	1
Sphyracephala	*europaea*	-	Metazoa	Arthropoda	Insecta	Diptera	*mariner*	1
Spodoptera	*litura*	-	Metazoa	Arthropoda	Insecta	Lepidoptera	*mariner*	2
Stegodyphus	*mimosarum*	-	Metazoa	Arthropoda	Arachnida	Araneae	*mariner*	10
Stomoxys	*uruma*	-	Metazoa	Arthropoda	Insecta	Diptera	*mariner*	1
Strongyloides	*ratti*	-	Metazoa	Nematoda	Chromadorea	Rhabditida	*mariner*	2
Tapinoma	*nigerrimum*	-	Metazoa	Arthropoda	Insecta	Hymenoptera	*mariner*	3
Tapinoma	*sessile*	-	Metazoa	Arthropoda	Insecta	Hymenoptera	*mariner*	1
Tatumella	*sp.*	-	Bacteria	Proteobacteria	Gammaproteobacteria	Enterobacterales	*mariner*	1
Teleopsis	*dalmanni*	-	Metazoa	Arthropoda	Insecta	Diptera	*mariner*	3
Trachymyrmex	*cornetzi*	-	Metazoa	Arthropoda	Insecta	Hymenoptera	*mariner*	1
Trachymyrmex	*septentrionalis*	-	Metazoa	Arthropoda	Insecta	Hymenoptera	*mariner*	20
Trachymyrmex	*zeteki*	-	Metazoa	Arthropoda	Insecta	Hymenoptera	*mariner*	6
Tribolium	*castaneum*	-	Metazoa	Arthropoda	Insecta	Coleoptera	*mariner*	5
Trichomonas	*vaginalis*	-	Parabasalia	Excavata	-	Trichomonadida	*mariner*	5
Trichuris	*suis*	-	Metazoa	Nematoda	Enoplea	Trichinellida	*mariner*	50
Tupaia	*chinensis*	-	Metazoa	Chordata	Mammalia	Scandentia	*mariner*	1
Ventiella	*sulfuris*	-	Metazoa	Arthropoda	Malacostraca	Amphipoda	*mariner*	2
Vollenhovia	*emeryi*	-	Metazoa	Arthropoda	Insecta	Hymenoptera	*mariner*	5
Wasmannia	*auropunctata*	-	Metazoa	Arthropoda	Insecta	Hymenoptera	*mariner*	1

Xenopus	*tropicalis*	-	Metazoa	Chordata	Amphibia	Anura	*mariner*	1
Abrus	*precatorius*	-	Plantae	Streptophyta	Eudicotyledons	Fabales	*pogo*	5
Acanthisitta	*chloris*	-	Metazoa	Chordata	Aves	Passeriformes	*pogo*	1
Acidomyces	*richmondensis*	-	Fungi	Ascomycota	Dothideomycetes	Capnodiales	*pogo*	1
Acinonyx	*jubatus*	-	Metazoa	Chordata	Mammalia	Carnivora	*pogo*	5
Acyrthosiphon	*pisum*	-	Metazoa	Arthropoda	Insecta	Hemiptera	*pogo*	35
Aedes	*aegypti*	-	Metazoa	Arthropoda	Insecta	Diptera	*pogo*	12
Aedes	*albopictus*	-	Metazoa	Arthropoda	Insecta	Diptera	*pogo*	5
Aethina	*tumida*	-	Metazoa	Arthropoda	Insecta	Coleoptera	*pogo*	1
Agrilus	*planipennis*	-	Metazoa	Arthropoda	Insecta	Coleoptera	*pogo*	5
Ailuropoda	*melanoleuca*	-	Metazoa	Chordata	Mammalia	Carnivora	*pogo*	7
Albugo	*laibachii*	-	Stramenopiles	-	Oomycetes	Albuginales	*pogo*	4
Alligator	*mississippiensis*	-	Metazoa	Chordata	Sauropsida	Crocodylia	*pogo*	9
Alligator	*sinensis*	-	Metazoa	Chordata	Sauropsida	Crocodylia	*pogo*	4
Alternaria	*alternata*	-	Fungi	Ascomycota	Dothideomycetes	Pleosporales	*pogo*	3
Alternaria	*arborescens*	-	Fungi	Ascomycota	Dothideomycetes	Pleosporales	*pogo*	7
Alternaria	*gaisen*	-	Fungi	Ascomycota	Dothideomycetes	Pleosporales	*pogo*	7
Alternaria	*sp.*	-	Fungi	Ascomycota	Dothideomycetes	Pleosporales	*pogo*	1
Alternaria	*tenuissima*	-	Fungi	Ascomycota	Dothideomycetes	Pleosporales	*pogo*	21
Amblyomma	*aureolatum*	-	Metazoa	Arthropoda	Arachnida	Ixodida	*pogo*	4
Amblyomma	*sculptum*	-	Metazoa	Arthropoda	Arachnida	Ixodida	*pogo*	2
Amblyomma	*triste*	-	Metazoa	Arthropoda	Arachnida	Ixodida	*pogo*	1
Amphimedon	*queenslandica*	-	Metazoa	Porifera	Demospongiae	Haplosclerida	*pogo*	1
Amphiprion	*ocellaris*	-	Metazoa	Chordata	Actinopteri	-	*pogo*	3
Amyelois	*transitella*	-	Metazoa	Arthropoda	Insecta	Lepidoptera	*pogo*	15
Anabarilius	*grahami*	-	Metazoa	Chordata	Actinopteri	Cypriniformes	*pogo*	2
Anabas	*testudineus*	-	Metazoa	Chordata	Actinopteri	Anabantiformes	*pogo*	2
Anolis	*carolinensis*	-	Metazoa	Chordata	Sauropsida	Squamata	*pogo*	4
Anopheles	*gambiae*	-	Metazoa	Arthropoda	Insecta	Diptera	*pogo*	1
Anoplophora	*glabripennis*	-	Metazoa	Arthropoda	Insecta	Coleoptera	*pogo*	12
Antrostomus	*carolinensis*	-	Metazoa	Chordata	Aves	Caprimulgiformes	*pogo*	1
Aotus	*nancymaae*	-	Metazoa	Chordata	Mammalia	Primates	*pogo*	3
Apaloderma	*vittatum*	-	Metazoa	Chordata	Aves	Trogoniformes	*pogo*	1
Aphanomyces	*invadans*	-	Stramenopiles	-	Oomycetes	Saprolegniales	*pogo*	2
Aphis	*gossypii*	-	Metazoa	Arthropoda	Insecta	Hemiptera	*pogo*	11
Aplysia	*californica*	-	Metazoa	Mollusca	Gastropoda	Aplysiida	*pogo*	6
Aptenodytes	*forsteri*	-	Metazoa	Chordata	Aves	Sphenisciformes	*pogo*	1
Apteryx	*australis*	-	Metazoa	Chordata	Aves	Apterygiformes	*pogo*	1
Apteryx	*rowi*	-	Metazoa	Chordata	Aves	Apterygiformes	*pogo*	2
Aquila	*chrysaetos*	-	Metazoa	Chordata	Aves	Accipitriformes	*pogo*	1
Arabidopsis	*thaliana*	-	Plantae	Streptophyta	Eudicotyledons	Brassicales	*pogo*	3
Aspergillus	*aculeatus*	-	Fungi	Ascomycota	Eurotiomycetes	Eurotiales	*pogo*	1
Aspergillus	*awamori*	-	Fungi	Ascomycota	Eurotiomycetes	Eurotiales	*pogo*	2
Aspergillus	*calidoustus*	-	Fungi	Ascomycota	Eurotiomycetes	Eurotiales	*pogo*	4
Aspergillus	*cristatus*	-	Fungi	Ascomycota	Eurotiomycetes	Eurotiales	*pogo*	39
Aspergillus	*fumigatus*	-	Fungi	Ascomycota	Eurotiomycetes	Eurotiales	*pogo*	4
Aspergillus	*glaucus*	-	Fungi	Ascomycota	Eurotiomycetes	Eurotiales	*pogo*	1
Aspergillus	*nidulans*	-	Fungi	Ascomycota	Eurotiomycetes	Eurotiales	*pogo*	15
Aspergillus	*niger*	-	Fungi	Ascomycota	Eurotiomycetes	Eurotiales	*pogo*	7
Aspergillus	*oryzae*	-	Fungi	Ascomycota	Eurotiomycetes	Eurotiales	*pogo*	3
Aspergillus	*ruber*	-	Fungi	Ascomycota	Eurotiomycetes	Eurotiales	*pogo*	2
Aspergillus	*sclerotialis*	-	Fungi	Ascomycota	Eurotiomycetes	Eurotiales	*pogo*	9
Aspergillus	*terreus*	-	Fungi	Ascomycota	Eurotiomycetes	Eurotiales	*pogo*	3
Aspergillus	*udagawae*	-	Fungi	Ascomycota	Eurotiomycetes	Eurotiales	*pogo*	2
Astatotilapia	*calliptera*	-	Metazoa	Chordata	Actinopteri	Cichliformes	*pogo*	4
Austrofundulus	*limnaeus*	-	Metazoa	Chordata	Actinopteri	Cyprinodontiformes	*pogo*	1
Bactrocera	*latifrons*	-	Metazoa	Arthropoda	Insecta	Diptera	*pogo*	1
Bactrocera	*oleae*	-	Metazoa	Arthropoda	Insecta	Diptera	*pogo*	1
Balaenoptera	*acutorostrata*	-	Metazoa	Chordata	Mammalia	Artiodactyla	*pogo*	5
Balearica	*regulorum*	-	Metazoa	Chordata	Aves	Gruiformes	*pogo*	1
Beijerinckia	*mobilis*	-	Bacteria	Proteobacteria	Alphaproteobacteria	Rhizobiales	*pogo*	1
Bemisia	*tabaci*	-	Metazoa	Arthropoda	Insecta	Hemiptera	*pogo*	2
Bicyclus	*anynana*	-	Metazoa	Arthropoda	Insecta	Lepidoptera	*pogo*	7
Bipolaris	*oryzae*	-	Fungi	Ascomycota	Dothideomycetes	Pleosporales	*pogo*	1
Bipolaris	*sorokiniana*	-	Fungi	Ascomycota	Dothideomycetes	Pleosporales	*pogo*	3
Bison	*bison*	-	Metazoa	Chordata	Mammalia	Artiodactyla	*pogo*	3
Blastomyces	*percursus*	-	Fungi	Ascomycota	Eurotiomycetes	Onygenales	*pogo*	2
Blattella	*germanica*	-	Metazoa	Arthropoda	Insecta	Blattodea	*pogo*	2
Bombyx	*mandarina*	-	Metazoa	Arthropoda	Insecta	Lepidoptera	*pogo*	6
Bombyx	*mori*	-	Metazoa	Arthropoda	Insecta	Lepidoptera	*pogo*	10
Bos	*indicus*	-	Metazoa	Chordata	Mammalia	Artiodactyla	*pogo*	10
Bos	*mutus*	-	Metazoa	Chordata	Mammalia	Artiodactyla	*pogo*	4
Bos	*taurus*	-	Metazoa	Chordata	Mammalia	Artiodactyla	*pogo*	10
Botrytis	*cinerea*	-	Fungi	Ascomycota	Leotiomycetes	Helotiales	*pogo*	2
Brassica	*rapa*	-	Plantae	Streptophyta	Eudicotyledons	Brassicales	*pogo*	1
Bubalus	*bubalis*	-	Metazoa	Chordata	Mammalia	Artiodactyla	*pogo*	8
Buceros	*rhinoceros*	-	Metazoa	Chordata	Aves	Bucerotiformes	*pogo*	1
Caenorhabditis	*elegans*	-	Metazoa	Nematoda	Chromadorea	Rhabditida	*pogo*	1
Cajanus	*cajan*	-	Plantae	Streptophyta	Eudicotyledons	Fabales	*pogo*	1
Calidris	*pugnax*	-	Metazoa	Chordata	Aves	Charadriiformes	*pogo*	1

Callithrix	*jacchus*	-	Metazoa	Chordata	Mammalia	Primates	*pogo*	4
Callorhinus	*ursinus*	-	Metazoa	Chordata	Mammalia	Carnivora	*pogo*	6
Calypte	*anna*	-	Metazoa	Chordata	Aves	Apodiformes	*pogo*	1
Camelina	*sativa*	-	Plantae	Streptophyta	Eudicotyledons	Brassicales	*pogo*	3
Camelus	*bactrianus*	-	Metazoa	Chordata	Mammalia	Artiodactyla	*pogo*	3
Camelus	*dromedarius*	-	Metazoa	Chordata	Mammalia	Artiodactyla	*pogo*	2
Camelus	*ferus*	-	Metazoa	Chordata	Mammalia	Artiodactyla	*pogo*	5
Candida	*albicans*	-	Fungi	Ascomycota	Saccharomycetes	Saccharomycetales	*pogo*	21
Candida	*dubliniensis*	-	Fungi	Ascomycota	Saccharomycetes	Saccharomycetales	*pogo*	1
Candida	*glabrata*	-	Fungi	Ascomycota	Saccharomycetes	Saccharomycetales	*pogo*	1
Canis	*lupus*	-	Metazoa	Chordata	Mammalia	Carnivora	*pogo*	7
Capitella	*teleta*	-	Metazoa	Annelida	Polychaeta	Capitellida	*pogo*	9
Capra	*hircus*	-	Metazoa	Chordata	Mammalia	Artiodactyla	*pogo*	6
Capsella	*rubella*	-	Plantae	Streptophyta	Eudicotyledons	Brassicales	*pogo*	2
Capsicum	*baccatum*	-	Plantae	Streptophyta	Eudicotyledons	Solanales	*pogo*	1
Cariama	*cristata*	-	Metazoa	Chordata	Aves	Cariamiformes	*pogo*	1
Carlito	*syrichta*	-	Metazoa	Chordata	Mammalia	Primates	*pogo*	2
Castor	*canadensis*	-	Metazoa	Chordata	Mammalia	Rodentia	*pogo*	1
Cathartes	*aura*	-	Metazoa	Chordata	Aves	Accipitriformes	*pogo*	1
Cavia	*aperea*	-	Metazoa	Chordata	Mammalia	Rodentia	*pogo*	1
Cavia	*porcellus*	-	Metazoa	Chordata	Mammalia	Rodentia	*pogo*	1
Cebus	*capucinus*	*imitator*	Metazoa	Chordata	Mammalia	Primates	*pogo*	3
Centruroides	*sculpturatus*	-	Metazoa	Arthropoda	Arachnida	Scorpiones	*pogo*	29
Ceratina	*calcarata*	-	Metazoa	Arthropoda	Insecta	Hymenoptera	*pogo*	1
Ceratotherium	*simum*	*simum*	Metazoa	Chordata	Mammalia	Perissodactyla	*pogo*	1
Ceratotherium	*simum*	-	Metazoa	Chordata	Mammalia	Perissodactyla	*pogo*	4
Cercocebus	*atys*	-	Metazoa	Chordata	Mammalia	Primates	*pogo*	2
Chaetomium	*globosum*	-	Fungi	Ascomycota	Sordariomycetes	Sordariales	*pogo*	14
Chaetura	*pelagica*	-	Metazoa	Chordata	Aves	Apodiformes	*pogo*	1
Charadrius	*vociferus*	-	Metazoa	Chordata	Aves	Charadriiformes	*pogo*	1
Chelonia	*mydas*	-	Metazoa	Chordata	Sauropsida	Testudines	*pogo*	7
Chilo	*suppressalis*	-	Metazoa	Arthropoda	Insecta	Lepidoptera	*pogo*	1
Chinchilla	*lanigera*	-	Metazoa	Chordata	Mammalia	Rodentia	*pogo*	5
Chlamydotis	*macqueenii*	-	Metazoa	Chordata	Aves	Gruiformes	*pogo*	1
Chlorocebus	*sabaeus*	-	Metazoa	Chordata	Mammalia	Primates	*pogo*	3
Choloepus	*hoffmanni*	-	Metazoa	Chordata	Mammalia	Pilosa	*pogo*	2
Chrysemys	*picta*	*bellii*	Metazoa	Chordata	Sauropsida	Testudines	*pogo*	3
Chrysemys	*picta*	-	Metazoa	Chordata	Sauropsida	Testudines	*pogo*	3
Chrysochloris	*asiatica*	-	Metazoa	Chordata	Mammalia	-	*pogo*	3
Cimex	*lectularius*	-	Metazoa	Arthropoda	Insecta	Hemiptera	*pogo*	1
Clastoptera	*arizonana*	-	Metazoa	Arthropoda	Insecta	Hemiptera	*pogo*	2
Coccidioides	*immitis*	-	Fungi	Ascomycota	Eurotiomycetes	Onygenales	*pogo*	1
Coleophoma	*cylindrospora*	-	Fungi	Ascomycota	Leotiomycetes	Helotiales	*pogo*	1
Colius	*striatus*	-	Metazoa	Chordata	Aves	Coliiformes	*pogo*	1
Colletotrichum	*chlorophyti*	-	Fungi	Ascomycota	Sordariomycetes	Glomerellales	*pogo*	1
Colletotrichum	*fructicola*	-	Fungi	Ascomycota	Sordariomycetes	Glomerellales	*pogo*	1
Colletotrichum	*higginsianum*	-	Fungi	Ascomycota	Sordariomycetes	Glomerellales	*pogo*	14
Colletotrichum	*incanum*	-	Fungi	Ascomycota	Sordariomycetes	Glomerellales	*pogo*	12
Colletotrichum	*sublineola*	-	Fungi	Ascomycota	Sordariomycetes	Glomerellales	*pogo*	3
Colletotrichum	*tofieldiae*	-	Fungi	Ascomycota	Sordariomycetes	Glomerellales	*pogo*	6
Colobus	*angolensis*	*palliatus*	Metazoa	Chordata	Mammalia	Primates	*pogo*	2
Columba	*livia*	-	Metazoa	Chordata	Aves	Columbiformes	*pogo*	1
Corvus	*brachyrhynchos*	-	Metazoa	Chordata	Aves	Passeriformes	*pogo*	1
Corvus	*cornix*	*cornix*	Metazoa	Chordata	Aves	Passeriformes	*pogo*	1
Crassostrea	*gigas*	-	Metazoa	Mollusca	Bivalvia	Ostreoida	*pogo*	19
Crassostrea	*virginica*	-	Metazoa	Mollusca	Bivalvia	Ostreoida	*pogo*	1
Cricetulus	*griseus*	-	Metazoa	Chordata	Mammalia	Rodentia	*pogo*	2
Crocodylus	*porosus*	-	Metazoa	Chordata	Sauropsida	Crocodylia	*pogo*	2
Cryptotermes	*secundus*	-	Metazoa	Arthropoda	Insecta	Blattodea	*pogo*	6
Ctenocephalides	*felis*	-	Metazoa	Arthropoda	Insecta	Siphonaptera	*pogo*	9
Cuculus	*canorus*	-	Metazoa	Chordata	Aves	Cuculiformes	*pogo*	1
Cuerna	*arida*	-	Metazoa	Arthropoda	Insecta	Hemiptera	*pogo*	1
Culex	*pipiens*	*quinquefasciatus*	Metazoa	Arthropoda	Insecta	Diptera	*pogo*	1
Culex	*quinquefasciatus*	-	Metazoa	Arthropoda	Insecta	Diptera	*pogo*	4
Cyanistes	*caeruleus*	-	Metazoa	Chordata	Aves	Passeriformes	*pogo*	2
Cynoglossus	*semilaevis*	-	Metazoa	Chordata	Actinopteri	Pleuronectiformes	*pogo*	1
Cyphomyrmex	*costatus*	-	Metazoa	Arthropoda	Insecta	Hymenoptera	*pogo*	5
Cyprinus	*carpio*	-	Metazoa	Chordata	Actinopteri	Cypriniformes	*pogo*	1
Danaus	*plexippus*	-	Metazoa	Arthropoda	Insecta	Lepidoptera	*pogo*	3
Daphnia	*pulex*	-	Metazoa	Arthropoda	Branchiopoda	Diplostraca	*pogo*	1
Dasypus	*novemcinctus*	-	Metazoa	Chordata	Mammalia	Cingulata	*pogo*	3
Delphinapterus	*leucas*	-	Metazoa	Chordata	Mammalia	Artiodactyla	*pogo*	5
Dendronephthya	*gigantea*	-	Metazoa	Cnidaria	Anthozoa	Alcyonacea	*pogo*	1
Dermatophagoides	*pteronyssinus*	-	Metazoa	Arthropoda	Arachnida	Sarcoptiformes	*pogo*	2
Desmodus	*rotundus*	-	Metazoa	Chordata	Mammalia	Chiroptera	*pogo*	3
Diabrotica	*virgifera*	-	Metazoa	Arthropoda	Insecta	Coleoptera	*pogo*	3
Diaphorina	*citri*	-	Metazoa	Arthropoda	Insecta	Hemiptera	*pogo*	2
Dinoponera	*quadriceps*	-	Metazoa	Arthropoda	Insecta	Hymenoptera	*pogo*	3
Dipodomys	*ordii*	-	Metazoa	Chordata	Mammalia	Rodentia	*pogo*	1
Diuraphis	*noxia*	-	Metazoa	Arthropoda	Insecta	Hemiptera	*pogo*	5

Drechmeria	*coniospora*	-	Fungi	Ascomycota	Sordariomycetes	Hypocreales	*pogo*	4
Dromaius	*novaehollandiae*	-	Metazoa	Chordata	Aves	Casuariiformes	*pogo*	5
Drosophila	*eugracilis*	-	Metazoa	Arthropoda	Insecta	Diptera	*pogo*	1
Drosophila	*ficusphila*	-	Metazoa	Arthropoda	Insecta	Diptera	*pogo*	1
Drosophila	*melanogaster*	-	Metazoa	Arthropoda	Insecta	Diptera	*pogo*	4
Dryobates	*pubescens*	-	Metazoa	Chordata	Aves	Piciformes	*pogo*	1
Dufourea	*novaeangliae*	-	Metazoa	Arthropoda	Insecta	Hymenoptera	*pogo*	3
Echinops	*telfairi*	-	Metazoa	Chordata	Mammalia	-	*pogo*	4
Egretta	*garzetta*	-	Metazoa	Chordata	Aves	Pelecaniformes	*pogo*	1
Elephantulus	*edwardii*	-	Metazoa	Chordata	Mammalia	Macroscelidea	*pogo*	3
Elysia	*chlorotica*	-	Metazoa	Mollusca	Gastropoda	-	*pogo*	2
Enhydra	*lutris*	*kenyoni*	Metazoa	Chordata	Mammalia	Carnivora	*pogo*	2
Enhydra	*lutris*	-	Metazoa	Chordata	Mammalia	Carnivora	*pogo*	3
Entamoeba	*invadens*	-	Amoebozoa	-	Archamoebae	-	*pogo*	1
Eptesicus	*fuscus*	-	Metazoa	Chordata	Mammalia	Chiroptera	*pogo*	3
Equus	*asinus*	-	Metazoa	Chordata	Mammalia	Perissodactyla	*pogo*	6
Equus	*caballus*	-	Metazoa	Chordata	Mammalia	Perissodactyla	*pogo*	6
Equus	*przewalskii*	-	Metazoa	Chordata	Mammalia	Perissodactyla	*pogo*	4
Erinaceus	*europaeus*	-	Metazoa	Chordata	Mammalia	Eulipotyphla	*pogo*	2
Eufriesea	*mexicana*	-	Metazoa	Arthropoda	Insecta	Hymenoptera	*pogo*	6
Eumetopias	*jubatus*	-	Metazoa	Chordata	Mammalia	Carnivora	*pogo*	3
Eurypyga	*helias*	-	Metazoa	Chordata	Aves	Gruiformes	*pogo*	1
Eutrema	*salsugineum*	-	Plantae	Streptophyta	Eudicotyledons	Brassicales	*pogo*	1
Exidia	*glandulosa*	-	Fungi	Basidiomycota	Agaricomycetes	Auriculariales	*pogo*	1
Exserohilum	*turcicum*	-	Fungi	Ascomycota	Pezizomycetes	Pleosporales	*pogo*	1
Falco	*peregrinus*	-	Metazoa	Chordata	Aves	Falconiformes	*pogo*	1
Felis	*catus*	-	Metazoa	Chordata	Mammalia	Carnivora	*pogo*	9
Francisella	*tularensis*	-	Bacteria	Proteobacteria	Gammaproteobacteria	Thiotrichales	*pogo*	1
Fukomys	*damarensis*	-	Metazoa	Chordata	Mammalia	Rodentia	*pogo*	2
Fulmarus	*glacialis*	-	Metazoa	Chordata	Aves	Procellariiformes	*pogo*	1
Fusarium	*fujikuroi*	-	Fungi	Ascomycota	Sordariomycetes	Hypocreales	*pogo*	11
Fusarium	*graminearum*	-	Fungi	Ascomycota	Sordariomycetes	Hypocreales	*pogo*	1
Fusarium	*mangiferae*	-	Fungi	Ascomycota	Sordariomycetes	Hypocreales	*pogo*	1
Fusarium	*nygamai*	-	Fungi	Ascomycota	Sordariomycetes	Hypocreales	*pogo*	7
Fusarium	*oxysporum*	-	Fungi	Ascomycota	Sordariomycetes	Hypocreales	*pogo*	340
Fusarium	*poae*	-	Fungi	Ascomycota	Sordariomycetes	Hypocreales	*pogo*	71
Fusarium	*proliferatum*	-	Fungi	Ascomycota	Sordariomycetes	Hypocreales	*pogo*	7
Fusarium	*sp.*	-	Fungi	Ascomycota	Sordariomycetes	Hypocreales	*pogo*	7
Fusarium	*venenatum*	-	Fungi	Ascomycota	Sordariomycetes	Hypocreales	*pogo*	1
Galendromus	*occidentalis*	-	Metazoa	Arthropoda	Arachnida	Mesostigmata	*pogo*	8
Galeopterus	*variegatus*	-	Metazoa	Chordata	Mammalia	Dermoptera	*pogo*	4
Galleria	*mellonella*	-	Metazoa	Arthropoda	Insecta	Lepidoptera	*pogo*	4
Gavia	*stellata*	-	Metazoa	Chordata	Aves	Gaviiformes	*pogo*	1
Gavialis	*gangeticus*	-	Metazoa	Chordata	Sauropsida	Crocodylia	*pogo*	6
Geospiza	*fortis*	-	Metazoa	Chordata	Aves	Passeriformes	*pogo*	1
Glycine	*max*	-	Plantae	Streptophyta	Eudicotyledons	Fabales	*pogo*	2
Glycine	*soja*	-	Plantae	Streptophyta	Eudicotyledons	Fabales	*pogo*	3
Gopherus	*agassizii*	-	Metazoa	Chordata	Sauropsida	Testudines	*pogo*	1
Gorilla	*gorilla*	*gorilla*	Metazoa	Chordata	Mammalia	Primates	*pogo*	4
Gossypium	*arboreum*	-	Plantae	Streptophyta	Eudicotyledons	Malvales	*pogo*	1
Gossypium	*raimondii*	-	Plantae	Streptophyta	Eudicotyledons	Malvales	*pogo*	1
Graphocephala	*atropunctata*	-	Metazoa	Arthropoda	Insecta	Hemiptera	*pogo*	3
Habropoda	*laboriosa*	-	Metazoa	Arthropoda	Insecta	Hymenoptera	*pogo*	3
Haliaeetus	*leucocephalus*	-	Metazoa	Chordata	Aves	Accipitriformes	*pogo*	1
Halyomorpha	*halys*	-	Metazoa	Arthropoda	Insecta	Hemiptera	*pogo*	8
Harpegnathos	*saltator*	-	Metazoa	Arthropoda	Insecta	Hymenoptera	*pogo*	7
Helianthus	*annuus*	-	Plantae	Streptophyta	Eudicotyledons	Asterales	*pogo*	1
Heliconius	*melpomene*	-	Metazoa	Arthropoda	Insecta	Lepidoptera	*pogo*	4
Helicoverpa	*armigera*	-	Metazoa	Arthropoda	Insecta	Lepidoptera	*pogo*	8
Heliothis	*virescens*	-	Metazoa	Arthropoda	Insecta	Lepidoptera	*pogo*	7
Helobdella	*robusta*	-	Metazoa	Annelida	Clitellata	Hirudinida	*pogo*	13
Heterocephalus	*glaber*	-	Metazoa	Chordata	Mammalia	Rodentia	*pogo*	2
Hippocampus	*comes*	-	Metazoa	Chordata	Actinopteri	Syngnathiformes	*pogo*	1
Hipposideros	*armiger*	-	Metazoa	Chordata	Mammalia	Chiroptera	*pogo*	7
Hirsutella	*minnesotensis*	-	Fungi	Ascomycota	Sordariomycetes	Hypocreales	*pogo*	12
Histoplasma	*capsulatum*	-	Fungi	Ascomycota	Eurotiomycetes	Onygenales	*pogo*	30
Homo	*sapiens*	-	Metazoa	Chordata	Mammalia	Primates	*pogo*	20
Hyalella	*azteca*	-	Metazoa	Arthropoda	Malacostraca	Amphipoda	*pogo*	1
Hydra	*vulgaris*	-	Metazoa	Cnidaria	Hydrozoa	Anthoathecata	*pogo*	93
Hymenobacter	*sp.*	-	Fungi	Bacteroidetes	Cytophagia	Cytophagales	*pogo*	1
Hyposmocoma	*kahamanoa*	-	Metazoa	Arthropoda	Insecta	Lepidoptera	*pogo*	7
Hypothenemus	*hampei*	-	Metazoa	Arthropoda	Insecta	Coleoptera	*pogo*	2
Ictidomys	*tridecemlineatus*	-	Metazoa	Chordata	Mammalia	Rodentia	*pogo*	3
Ixodes	*ricinus*	-	Metazoa	Arthropoda	Arachnida	Ixodida	*pogo*	3
Jaculus	*jaculus*	-	Metazoa	Chordata	Mammalia	Rodentia	*pogo*	1
Kryptolebias	*marmoratus*	-	Metazoa	Chordata	Actinopteri	Cyprinodontiformes	*pogo*	1
Labeo	*rohita*	-	Metazoa	Chordata	Actinopteri	Cypriniformes	*pogo*	4
Lactuca	*sativa*	-	Plantae	Streptophyta	Eudicotyledons	Asterales	*pogo*	2
Lagenorhynchus	*obliquidens*	-	Metazoa	Chordata	Mammalia	Artiodactyla	*pogo*	4
Laodelphax	*striatellus*	-	Metazoa	Arthropoda	Insecta	Hemiptera	*pogo*	1

Lasius	*niger*	-	Metazoa	Arthropoda	Insecta	Hymenoptera	*pogo*	2
Latimeria	*chalumnae*	-	Metazoa	Chordata	-	Coelacanthiformes	*pogo*	12
Lepidothrix	*coronata*	-	Metazoa	Chordata	Aves	Passeriformes	*pogo*	1
Lepisosteus	*oculatus*	-	Metazoa	Chordata	Actinopteri	Semionotiformes	*pogo*	1
Leptinotarsa	*decemlineata*	-	Metazoa	Arthropoda	Insecta	Coleoptera	*pogo*	9
Leptonychotes	*weddellii*	-	Metazoa	Chordata	Mammalia	Carnivora	*pogo*	5
Leptosomus	*discolor*	-	Metazoa	Chordata	Aves	Coraciiformes	*pogo*	1
Limosa	*lapponica*	*baueri*	Metazoa	Chordata	Aves	Charadriiformes	*pogo*	1
Lipomyces	*starkeyi*	-	Fungi	Ascomycota	Saccharomycetes	Saccharomycetales	*pogo*	1
Lipotes	*vexillifer*	-	Metazoa	Chordata	Mammalia	Artiodactyla	*pogo*	4
Locusta	*migratoria*	-	Metazoa	Arthropoda	Insecta	Orthoptera	*pogo*	1
Lonchura	*striata*	*domestica*	Metazoa	Chordata	Aves	Passeriformes	*pogo*	1
Loxodonta	*africana*	-	Metazoa	Chordata	Mammalia	Proboscidea	*pogo*	6
Lynx	*pardinus*	-	Metazoa	Chordata	Mammalia	Carnivora	*pogo*	1
Macaca	*mulatta*	-	Metazoa	Chordata	Mammalia	Primates	*pogo*	3
Macaca	*nemestrina*	-	Metazoa	Chordata	Mammalia	Primates	*pogo*	2
Magnaporthe	*grisea*	-	Fungi	Ascomycota	Pezizomycetes	Sordariales	*pogo*	1
Manacus	*vitellinus*	-	Metazoa	Chordata	Aves	Passeriformes	*pogo*	1
Mandrillus	*leucophaeus*	-	Metazoa	Chordata	Mammalia	Primates	*pogo*	2
Manis	*javanica*	-	Metazoa	Chordata	Mammalia	Pholidota	*pogo*	1
Marchantia	*polymorpha*	-	Plantae	Streptophyta	Marchantiopsida	Marchantiales	*pogo*	3
Marmota	*marmota*	*marmota*	Metazoa	Chordata	Mammalia	Rodentia	*pogo*	1
Marmota	*marmota*	-	Metazoa	Chordata	Mammalia	Rodentia	*pogo*	2
Mayetiola	*destructor*	-	Metazoa	Arthropoda	Insecta	Diptera	*pogo*	1
Medicago	*truncatula*	-	Plantae	Streptophyta	Eudicotyledons	Fabales	*pogo*	19
Megachile	*rotundata*	-	Metazoa	Arthropoda	Insecta	Hymenoptera	*pogo*	5
Melanaphis	*sacchari*	-	Metazoa	Arthropoda	Insecta	Hemiptera	*pogo*	14
Melitaea	*cinxia*	-	Metazoa	Arthropoda	Insecta	Lepidoptera	*pogo*	1
Melopsittacus	*undulatus*	-	Metazoa	Chordata	Aves	Psittaciformes	*pogo*	1
Merops	*nubicus*	-	Metazoa	Chordata	Aves	Coraciiformes	*pogo*	1
Mesitornis	*unicolor*	-	Metazoa	Chordata	Aves	Gruiformes	*pogo*	1
Mesocricetus	*auratus*	-	Metazoa	Chordata	Mammalia	Rodentia	*pogo*	1
Metarhizium	*anisopliae*	-	Fungi	Ascomycota	Sordariomycetes	Hypocreales	*pogo*	2
Metarhizium	*guizhouense*	-	Fungi	Ascomycota	Sordariomycetes	Hypocreales	*pogo*	4
Metarhizium	*majus*	-	Fungi	Ascomycota	Sordariomycetes	Hypocreales	*pogo*	1
Metarhizium	*robertsii*	-	Fungi	Ascomycota	Sordariomycetes	Hypocreales	*pogo*	3
Metaseiulus	*occidentalis*	-	Metazoa	Arthropoda	Arachnida	Mesostigmata	*pogo*	1
Metschnikowia	*sp.*	-	Fungi	Ascomycota	Saccharomycetes	Saccharomycetales	*pogo*	2
Microcebus	*murinus*	-	Metazoa	Chordata	Mammalia	Primates	*pogo*	2
Microplitis	*demolitor*	-	Metazoa	Arthropoda	Insecta	Hymenoptera	*pogo*	4
Microtus	*ochrogaster*	-	Metazoa	Chordata	Mammalia	Rodentia	*pogo*	1
Miniopterus	*natalensis*	-	Metazoa	Chordata	Mammalia	Chiroptera	*pogo*	2
Monodelphis	*domestica*	-	Metazoa	Chordata	Mammalia	Didelphimorphia	*pogo*	23
Monopterus	*albus*	-	Metazoa	Chordata	Actinopteri	Synbranchiformes	*pogo*	1
Monosporascus	*ibericus*	-	Fungi	Ascomycota	Sordariomycetes	Xylariales	*pogo*	1
Monosporascus	*sp.*	-	Fungi	Ascomycota	Sordariomycetes	Xylariales	*pogo*	2
Morchella	*conica*	-	Fungi	Ascomycota	Pezizomycetes	Pezizales	*pogo*	1
Mus	*caroli*	-	Metazoa	Chordata	Mammalia	Rodentia	*pogo*	1
Mus	*musculus*	-	Metazoa	Chordata	Mammalia	Rodentia	*pogo*	2
Mus	*pahari*	-	Metazoa	Chordata	Mammalia	Rodentia	*pogo*	1
Musca	*domestica*	-	Metazoa	Arthropoda	Insecta	Diptera	*pogo*	3
Mustela	*putorius*	*furo*	Metazoa	Chordata	Mammalia	Carnivora	*pogo*	3
Mustela	*putorius*	-	Metazoa	Chordata	Mammalia	Carnivora	*pogo*	5
Myotis	*brandtii*	-	Metazoa	Chordata	Mammalia	Chiroptera	*pogo*	4
Myotis	*davidii*	-	Metazoa	Chordata	Mammalia	Chiroptera	*pogo*	1
Myotis	*lucifugus*	-	Metazoa	Chordata	Mammalia	Chiroptera	*pogo*	6
Myzus	*cerasi*	-	Metazoa	Arthropoda	Insecta	Hemiptera	*pogo*	1
Myzus	*persicae*	*nicotianae*	Metazoa	Arthropoda	Insecta	Hemiptera	*pogo*	17
Myzus	*persicae*	-	Metazoa	Arthropoda	Insecta	Hemiptera	*pogo*	13
Nannospalax	*galili*	-	Metazoa	Chordata	Mammalia	Rodentia	*pogo*	1
Nanorana	*parkeri*	-	Metazoa	Chordata	Amphibia	Anura	*pogo*	9
Nectria	*haematococca*	-	Fungi	Ascomycota	Pezizomycetes	Sordariales	*pogo*	2
Neomonachus	*schauinslandi*	-	Metazoa	Chordata	Mammalia	Carnivora	*pogo*	4
Neophocaena	*asiaeorientalis*	*asiaeorientalis*	Metazoa	Chordata	Mammalia	Artiodactyla	*pogo*	1
Neophocaena	*asiaeorientalis*	-	Metazoa	Chordata	Mammalia	Artiodactyla	*pogo*	7
Neovison	*vison*	-	Metazoa	Chordata	Mammalia	Carnivora	*pogo*	4
Nestor	*notabilis*	-	Metazoa	Chordata	Aves	Psittaciformes	*pogo*	1
Nicotiana	*tomentosiformis*	-	Plantae	Streptophyta	Eudicotyledons	Solanales	*pogo*	1
Nilaparvata	*lugens*	-	Metazoa	Arthropoda	Insecta	Hemiptera	*pogo*	11
Nipponia	*nippon*	-	Metazoa	Chordata	Aves	Pelecaniformes	*pogo*	1
Nomascus	*leucogenys*	-	Metazoa	Chordata	Mammalia	Primates	*pogo*	3
Notamacropus	*eugenii*	-	Metazoa	Chordata	Mammalia	Diprotodontia	*pogo*	1
Notechis	*scutatus*	-	Metazoa	Chordata	Sauropsida	Squamata	*pogo*	5
Nuttalliella	*namaqua*	-	Metazoa	Arthropoda	Arachnida	Ixodida	*pogo*	1
Ochotona	*princeps*	-	Metazoa	Chordata	Mammalia	Lagomorpha	*pogo*	1
Octodon	*degus*	-	Metazoa	Chordata	Mammalia	Rodentia	*pogo*	2
Octopus	*bimaculoides*	-	Metazoa	Mollusca	Cephalopoda	Octopoda	*pogo*	5
Odobenus	*rosmarus*	*divergens*	Metazoa	Chordata	Mammalia	Carnivora	*pogo*	1
Odobenus	*rosmarus*	-	Metazoa	Chordata	Mammalia	Carnivora	*pogo*	3
Odocoileus	*virginianus*	*texanus*	Metazoa	Chordata	Mammalia	Artiodactyla	*pogo*	1

Odocoileus	*virginianus*	-	Metazoa	Chordata	Mammalia	Artiodactyla	*pogo*	8
Oidiodendron	*maius*	-	Fungi	Ascomycota	Leotiomycetes	-	*pogo*	1
Olea	*europaea*	-	Plantae	Streptophyta	Eudicotyledons	Lamiales	*pogo*	11
Onthophagus	*taurus*	-	Metazoa	Arthropoda	Insecta	Coleoptera	*pogo*	17
Ophiophagus	*hannah*	-	Metazoa	Chordata	Sauropsida	Squamata	*pogo*	1
Ophiostoma	*novo-ulmi*	-	Fungi	Ascomycota	Sordariomycetes	Ophiostomatales	*pogo*	1
Ophiostoma	*ulmi*	-	Fungi	Ascomycota	Sordariomycetes	Ophiostomatales	*pogo*	1
Opisthocomus	*hoazin*	-	Metazoa	Chordata	Aves	Opisthocomiformes	*pogo*	1
Orchesella	*cincta*	-	Metazoa	Arthropoda	Collembola	Entomobryomorpha	*pogo*	2
Orcinus	*orca*	-	Metazoa	Chordata	Mammalia	Artiodactyla	*pogo*	5
Oreochromis	*niloticus*	-	Metazoa	Chordata	Actinopteri	Cichliformes	*pogo*	5
Ornithodoros	*erraticus*	-	Metazoa	Arthropoda	Arachnida	Ixodida	*pogo*	1
Ornithorhynchus	*anatinus*	-	Metazoa	Chordata	Mammalia	Monotremata	*pogo*	3
Orussus	*abietinus*	-	Metazoa	Arthropoda	Insecta	Hymenoptera	*pogo*	1
Orycteropus	*afer*	*afer*	Metazoa	Chordata	Mammalia	Tubulidentata	*pogo*	1
Orycteropus	*afer*	-	Metazoa	Chordata	Mammalia	Tubulidentata	*pogo*	2
Oryctolagus	*cuniculus*	-	Metazoa	Chordata	Mammalia	Lagomorpha	*pogo*	3
Oryzias	*latipes*	-	Metazoa	Chordata	Actinopteri	Beloniformes	*pogo*	1
Oryzias	*melastigma*	-	Metazoa	Chordata	Actinopteri	Beloniformes	*pogo*	1
Ostrinia	*furnacalis*	-	Metazoa	Arthropoda	Insecta	Lepidoptera	*pogo*	2
Otolemur	*garnettii*	-	Metazoa	Chordata	Mammalia	Primates	*pogo*	3
Ovis	*aries*	-	Metazoa	Chordata	Mammalia	Artiodactyla	*pogo*	21
Pan	*paniscus*	-	Metazoa	Chordata	Mammalia	Primates	*pogo*	3
Pan	*troglodytes*	-	Metazoa	Chordata	Mammalia	Primates	*pogo*	3
Panthera	*pardus*	-	Metazoa	Chordata	Mammalia	Carnivora	*pogo*	8
Panthera	*tigris*	*altaica*	Metazoa	Chordata	Mammalia	Carnivora	*pogo*	2
Panthera	*tigris*	-	Metazoa	Chordata	Mammalia	Carnivora	*pogo*	4
Pantholops	*hodgsonii*	-	Metazoa	Chordata	Mammalia	Artiodactyla	*pogo*	4
Papaver	*somniferum*	-	Plantae	Streptophyta	Dicotyledons	Ranunculales	*pogo*	1
Papilio	*machaon*	-	Metazoa	Arthropoda	Insecta	Lepidoptera	*pogo*	3
Papilio	*xuthus*	-	Metazoa	Arthropoda	Insecta	Lepidoptera	*pogo*	9
Papio	*anubis*	-	Metazoa	Chordata	Mammalia	Primates	*pogo*	2
Paracoccidioides	*brasiliensis*	-	Fungi	Ascomycota	Eurotiomycetes	Onygenales	*pogo*	3
Paramormyrops	*kingsleyae*	-	Metazoa	Chordata	Actinopteri	Osteoglossiformes	*pogo*	19
Parastagonospora	*nodorum*	-	Fungi	Ascomycota	Dothideomycetes	Pleosporales	*pogo*	1
Parasteatoda	*tepidariorum*	-	Metazoa	Arthropoda	Arachnida	Araneae	*pogo*	9
Paroedura	*picta*	-	Metazoa	Chordata	Sauropsida	Squamata	*pogo*	1
Parus	*major*	-	Metazoa	Chordata	Aves	Passeriformes	*pogo*	1
Patagioenas	*fasciata*	-	Metazoa	Chordata	Aves	Columbiformes	*pogo*	1
Pelecanus	*crispus*	-	Metazoa	Chordata	Aves	Pelecaniformes	*pogo*	1
Pelodiscus	*sinensis*	-	Metazoa	Chordata	Sauropsida	Testudines	*pogo*	9
Penicillium	*antarcticum*	-	Fungi	Ascomycota	Eurotiomycetes	Eurotiales	*pogo*	11
Penicillium	*arizonense*	-	Fungi	Ascomycota	Eurotiomycetes	Eurotiales	*pogo*	3
Penicillium	*brasilianum*	-	Fungi	Ascomycota	Eurotiomycetes	Eurotiales	*pogo*	21
Penicillium	*camemberti*	-	Fungi	Ascomycota	Eurotiomycetes	Eurotiales	*pogo*	4
Penicillium	*digitatum*	-	Fungi	Ascomycota	Eurotiomycetes	Eurotiales	*pogo*	5
Penicillium	*flavigenum*	-	Fungi	Ascomycota	Eurotiomycetes	Eurotiales	*pogo*	2
Penicillium	*griseofulvum*	-	Fungi	Ascomycota	Eurotiomycetes	Eurotiales	*pogo*	1
Penicillium	*oxalicum*	-	Fungi	Ascomycota	Eurotiomycetes	Eurotiales	*pogo*	1
Penicillium	*roqueforti*	-	Fungi	Ascomycota	Eurotiomycetes	Eurotiales	*pogo*	2
Penicillium	*rubens*	-	Fungi	Ascomycota	Eurotiomycetes	Eurotiales	*pogo*	5
Penicillium	*subrubescens*	-	Fungi	Ascomycota	Eurotiomycetes	Eurotiales	*pogo*	4
Penicillium	*vulpinum*	-	Fungi	Ascomycota	Eurotiomycetes	Eurotiales	*pogo*	2
Peromyscus	*maniculatus*	*bairdii*	Metazoa	Chordata	Mammalia	Rodentia	*pogo*	1
Pestalotiopsis	*fici*	-	Fungi	Ascomycota	Sordariomycetes	Xylariales	*pogo*	1
Phaethon	*lepturus*	-	Metazoa	Chordata	Aves	Pelecaniformes	*pogo*	1
Phalacrocorax	*carbo*	-	Metazoa	Chordata	Aves	Pelecaniformes	*pogo*	1
Phascolarctos	*cinereus*	-	Metazoa	Chordata	Mammalia	Diprotodontia	*pogo*	9
Phoenicopterus	*ruber*	*ruber*	Metazoa	Chordata	Aves	Phoenicopteriformes	*pogo*	1
Phyllostomus	*discolor*	-	Metazoa	Chordata	Mammalia	Chiroptera	*pogo*	1
Physeter	*catodon*	-	Metazoa	Chordata	Mammalia	Artiodactyla	*pogo*	4
Phytophthora	*sojae*	-	Stramenopiles	-	Oomycetes	Peronosporales	*pogo*	7
Picoides	*pubescens*	-	Metazoa	Chordata	Aves	Piciformes	*pogo*	1
Pieris	*rapae*	-	Metazoa	Arthropoda	Insecta	Lepidoptera	*pogo*	1
Piliocolobus	*tephrosceles*	-	Metazoa	Chordata	Mammalia	Primates	*pogo*	3
Plutella	*xylostella*	-	Metazoa	Arthropoda	Insecta	Lepidoptera	*pogo*	1
Pochonia	*chlamydosporia*	-	Fungi	Ascomycota	Sordariomycetes	Hypocreales	*pogo*	6
Podospora	*comata*	-	Fungi	Ascomycota	Sordariomycetes	Sordariales	*pogo*	2
Poecilia	*formosa*	-	Metazoa	Chordata	Actinopteri	Cyprinodontiformes	*pogo*	1
Poecilia	*mexicana*	-	Metazoa	Chordata	Actinopteri	Cyprinodontiformes	*pogo*	2
Poecilia	*reticulata*	-	Metazoa	Chordata	Actinopteri	Cyprinodontiformes	*pogo*	1
Pogona	*vitticeps*	-	Metazoa	Chordata	Sauropsida	Squamata	*pogo*	4
Polistes	*dominula*	-	Metazoa	Arthropoda	Insecta	Hymenoptera	*pogo*	1
Pomacea	*canaliculata*	-	Metazoa	Mollusca	Gastropoda	Architaenioglossa	*pogo*	2
Pongo	*abelii*	-	Metazoa	Chordata	Mammalia	Primates	*pogo*	3
Procavia	*capensis*	-	Metazoa	Chordata	Mammalia	Hyracoidea	*pogo*	1
Propithecus	*coquereli*	-	Metazoa	Chordata	Mammalia	Primates	*pogo*	3
Protobothrops	*mucrosquamatus*	-	Metazoa	Chordata	Sauropsida	Squamata	*pogo*	1
Pseudogymnoascus	*destructans*	-	Fungi	Ascomycota	Leotiomycetes	-	*pogo*	3
Pseudogymnoascus	*sp.*	-	Fungi	Ascomycota	Leotiomycetes	-	*pogo*	18

Pseudogymnoascus	*verrucosus*	-	Fungi	Ascomycota	Leotiomycetes	-	*pogo*	1
Pseudomyrmex	*gracilis*	-	Metazoa	Arthropoda	Insecta	Hymenoptera	*pogo*	1
Pseudonaja	*textilis*	-	Metazoa	Chordata	Sauropsida	Squamata	*pogo*	8
Pseudopodoces	*humilis*	-	Metazoa	Chordata	Aves	Passeriformes	*pogo*	1
Pterocles	*gutturalis*	-	Metazoa	Chordata	Aves	Ciconiiformes	*pogo*	1
Pteropus	*alecto*	-	Metazoa	Chordata	Mammalia	Chiroptera	*pogo*	9
Pteropus	*vampyrus*	-	Metazoa	Chordata	Mammalia	Chiroptera	*pogo*	6
Puma	*concolor*	-	Metazoa	Chordata	Mammalia	Carnivora	*pogo*	4
Purpureocillium	*lilacinum*	-	Fungi	Ascomycota	Sordariomycetes	Hypocreales	*pogo*	7
Pygoscelis	*adeliae*	-	Metazoa	Chordata	Aves	Sphenisciformes	*pogo*	1
Pyrenophora	*teres*	-	Fungi	Ascomycota	Dothideomycetes	Pleosporales	*pogo*	8
Pyricularia	*grisea*	-	Fungi	Ascomycota	Sordariomycetes	Magnaporthales	*pogo*	2
Python	*bivittatus*	-	Metazoa	Chordata	Sauropsida	Squamata	*pogo*	6
Rasamsonia	*emersonii*	-	Fungi	Ascomycota	Eurotiomycetes	Eurotiales	*pogo*	7
Rattus	*norvegicus*	-	Metazoa	Chordata	Mammalia	Rodentia	*pogo*	1
Rhagoletis	*zephyria*	-	Metazoa	Arthropoda	Insecta	Diptera	*pogo*	4
Rhincodon	*typus*	-	Metazoa	Chordata	Chondrichthyes	Orectolobiformes	*pogo*	1
Rhinolophus	*sinicus*	-	Metazoa	Chordata	Mammalia	Chiroptera	*pogo*	1
Rhinopithecus	*bieti*	-	Metazoa	Chordata	Mammalia	Primates	*pogo*	2
Rhinopithecus	*roxellana*	-	Metazoa	Chordata	Mammalia	Primates	*pogo*	3
Rhipicephalus	*pulchellus*	-	Metazoa	Arthropoda	Arachnida	Ixodida	*pogo*	1
Rhipicephalus	*zambeziensis*	-	Metazoa	Arthropoda	Arachnida	Ixodida	*pogo*	1
Rhizophagus	*irregularis*	-	Fungi	Mucoromycota	Glomeromycetes	Glomerales	*pogo*	9
Rhynchosporium	*secalis*	-	Fungi	Ascomycota	Leotiomycetes	Helotiales	*pogo*	1
Ricinus	*communis*	-	Plantae	Streptophyta	Eudicotyledons	Malpighiales	*pogo*	1
Rousettus	*aegyptiacus*	-	Metazoa	Chordata	Mammalia	Chiroptera	*pogo*	4
Saccharomyces	*cerevisiae*	-	Fungi	Ascomycota	Saccharomycetes	Saccharomycetales	*pogo*	1
Saccoglossus	*kowalevskii*	-	Metazoa	Hemichordata	Enteropneusta	-	*pogo*	10
Saimiri	*boliviensis*	*boliviensis*	Metazoa	Chordata	Mammalia	Primates	*pogo*	2
Saimiri	*boliviensis*	-	Metazoa	Chordata	Mammalia	Primates	*pogo*	2
Sarcophilus	*harrisii*	-	Metazoa	Chordata	Mammalia	Dasyuromorphia	*pogo*	7
Schizaphis	*graminum*	-	Metazoa	Arthropoda	Insecta	Hemiptera	*pogo*	2
Schizosaccharomyces	*pombe*	-	Fungi	Ascomycota	Schizosacchatomycetes	-	*pogo*	3
Schmidtea	*mediterranea*	-	Metazoa	Platyhelminthes	Rhabditophora	Tricladida	*pogo*	5
Scleroderma	*citrinum*	-	Fungi	Basidiomycota	Agaricomycetes	Boletales	*pogo*	1
Scleropages	*formosus*	-	Metazoa	Chordata	Actinopteri	Osteoglossiformes	*pogo*	4
Sclerotinia	*sclerotiorum*	-	Fungi	Ascomycota	Leotiomycetes	Helotiales	*pogo*	6
Scytalidium	*lignicola*	-	Fungi	Ascomycota	Leotiomycetes	-	*pogo*	2
Selaginella	*moellendorffii*	-	Plantae	Streptophyta	Lycopodiopsida	Selaginellales	*pogo*	2
Serinus	*canaria*	-	Metazoa	Chordata	Aves	Passeriformes	*pogo*	1
Sesamum	*indicum*	-	Plantae	Streptophyta	Eudicotyledons	Lamiales	*pogo*	1
Sinocyclocheilus	*anshuiensis*	-	Metazoa	Chordata	Actinopteri	Cypriniformes	*pogo*	2
Sinocyclocheilus	*rhinocerous*	-	Metazoa	Chordata	Actinopteri	Cypriniformes	*pogo*	2
Sipha	*flava*	-	Metazoa	Arthropoda	Insecta	Hemiptera	*pogo*	23
Solenopsis	*invicta*	-	Metazoa	Arthropoda	Insecta	Hymenoptera	*pogo*	3
Sorex	*araneus*	-	Metazoa	Chordata	Mammalia	Eulipotyphla	*pogo*	1
Sousa	*chinensis*	-	Metazoa	Chordata	Mammalia	Artiodactyla	*pogo*	3
Sphaerobolus	*stellatus*	-	Fungi	Basidiomycota	Agaricomycetes	Geastrales	*pogo*	1
Spodoptera	*frugiperda*	-	Metazoa	Arthropoda	Insecta	Lepidoptera	*pogo*	1
Spodoptera	*litura*	-	Metazoa	Arthropoda	Insecta	Lepidoptera	*pogo*	6
Stachybotrys	*chartarum*	-	Fungi	Ascomycota	Sordariomycetes	Hypocreales	*pogo*	2
Stegodyphus	*mimosarum*	-	Metazoa	Arthropoda	Arachnida	Araneae	*pogo*	25
Stemphylium	*lycopersici*	-	Fungi	Ascomycota	Dothideomycetes	Pleosporales	*pogo*	6
Strongylocentrotus	*purpuratus*	-	Metazoa	Echinodermata	Echinoidea	Echinoida	*pogo*	2
Struthio	*camelus*	*australis*	Metazoa	Chordata	Aves	Struthioniformes	*pogo*	1
Sugiyamaella	*lignohabitans*	-	Fungi	Ascomycota	Saccharomycetes	Saccharomycetales	*pogo*	1
Sus	*scrofa*	-	Metazoa	Chordata	Mammalia	Artiodactyla	*pogo*	5
Tachysurus	*fulvidraco*	-	Metazoa	Chordata	Actinopteri	Siluriformes	*pogo*	3
Taeniopygia	*guttata*	-	Metazoa	Chordata	Aves	Passeriformes	*pogo*	1
Takifugu	*rubripes*	-	Metazoa	Chordata	Actinopteri	Tetraodontiformes	*pogo*	2
Talaromyces	*marneffei*	-	Fungi	Ascomycota	Eurotiomycetes	Eurotiales	*pogo*	21
Talaromyces	*stipitatus*	-	Fungi	Ascomycota	Eurotiomycetes	Eurotiales	*pogo*	85
Tarsius	*syrichta*	-	Metazoa	Chordata	Mammalia	Primates	*pogo*	1
Tauraco	*erythrolophus*	-	Metazoa	Chordata	Aves	Musophagiformes	*pogo*	1
Temnothorax	*curvispinosus*	-	Metazoa	Arthropoda	Insecta	Hymenoptera	*pogo*	3
Terrapene	*mexicana*	*triunguis*	Metazoa	Chordata	Sauropsida	Testudines	*pogo*	1
Terrapene	*mexicana*	-	Metazoa	Chordata	Sauropsida	Testudines	*pogo*	5
Thamnophis	*sirtalis*	-	Metazoa	Chordata	Sauropsida	Squamata	*pogo*	2
Theropithecus	*gelada*	-	Metazoa	Chordata	Mammalia	Primates	*pogo*	1
Tilletia	*indica*	-	Fungi	Basidiomycota	Exobasidiomycetes	Tilletiales	*pogo*	1
Tilletia	*walkeri*	-	Fungi	Basidiomycota	Exobasidiomycetes	Tilletiales	*pogo*	1
Tinamus	*guttatus*	-	Metazoa	Chordata	Aves	Tinamiformes	*pogo*	1
Trachymyrmex	*cornetzi*	-	Metazoa	Arthropoda	Insecta	Hymenoptera	*pogo*	2
Tribolium	*castaneum*	-	Metazoa	Arthropoda	Insecta	Coleoptera	*pogo*	3
Trichechus	*manatus*	*latirostris*	Metazoa	Chordata	Mammalia	Sirenia	*pogo*	2
Trichechus	*manatus*	-	Metazoa	Chordata	Mammalia	Sirenia	*pogo*	3
Trichinella	*britovi*	-	Metazoa	Nematoda	Enoplea	Trichinellida	*pogo*	5
Trichinella	*murrelli*	-	Metazoa	Nematoda	Enoplea	Trichinellida	*pogo*	2
Trichinella	*nativa*	-	Metazoa	Nematoda	Enoplea	Trichinellida	*pogo*	5
Trichinella	*nelsoni*	-	Metazoa	Nematoda	Enoplea	Trichinellida	*pogo*	1

Trichinella	*papuae*	-	Metazoa	Nematoda	Enoplea	Trichinellida	*pogo*	5
Trichinella	*patagoniensis*	-	Metazoa	Nematoda	Enoplea	Trichinellida	*pogo*	2
Trichinella	*pseudospiralis*	-	Metazoa	Nematoda	Enoplea	Trichinellida	*pogo*	1
Trichinella	*sp.*	-	Metazoa	Nematoda	Enoplea	Trichinellida	*pogo*	3
Trichinella	*sp. T6*	-	Metazoa	Nematoda	Enoplea	Trichinellida	*pogo*	2
Trichinella	*sp. T8*	-	Metazoa	Nematoda	Enoplea	Trichinellida	*pogo*	1
Trichinella	*sp. T9*	-	Metazoa	Nematoda	Enoplea	Trichinellida	*pogo*	2
Trichinella	*spiralis*	-	Metazoa	Nematoda	Enoplea	Trichinellida	*pogo*	2
Trichinella	*zimbabwensis*	-	Metazoa	Nematoda	Enoplea	Trichinellida	*pogo*	3
Trichogramma	*pretiosum*	-	Metazoa	Arthropoda	Insecta	Hymenoptera	*pogo*	4
Trichoplusia	*ni*	-	Metazoa	Arthropoda	Insecta	Lepidoptera	*pogo*	8
Trichuris	*suis*	-	Metazoa	Nematoda	Enoplea	Trichinellida	*pogo*	28
Tulasnella	*calospora*	-	Fungi	Basidiomycota	Agaricomycetes	Cantharellales	*pogo*	1
Tupaia	*belangeri*	-	Metazoa	Chordata	Mammalia	Scandentia	*pogo*	2
Tupaia	*chinensis*	-	Metazoa	Chordata	Mammalia	Scandentia	*pogo*	2
Tursiops	*truncatus*	-	Metazoa	Chordata	Mammalia	Artiodactyla	*pogo*	4
Tyto	*alba*	-	Metazoa	Chordata	Aves	Strigiformes	*pogo*	1
Umbilicaria	*pustulata*	-	Fungi	Ascomycota	Lecanoromycetes	Umbilicariales	*pogo*	11
Urocitellus	*parryii*	-	Metazoa	Chordata	Mammalia	Rodentia	*pogo*	2
Ursus	*americanus*	-	Metazoa	Chordata	Mammalia	Carnivora	*pogo*	2
Ursus	*arctos*	*horribilis*	Metazoa	Chordata	Mammalia	Carnivora	*pogo*	1
Ursus	*arctos*	-	Metazoa	Chordata	Mammalia	Carnivora	*pogo*	4
Ursus	*maritimus*	-	Metazoa	Chordata	Mammalia	Carnivora	*pogo*	4
Vanessa	*tameamea*	-	Metazoa	Arthropoda	Insecta	Lepidoptera	*pogo*	1
Verticillium	*dahliae*	-	Fungi	Ascomycota	Sordariomycetes	Glomerellales	*pogo*	5
Vicugna	*pacos*	-	Metazoa	Chordata	Mammalia	Artiodactyla	*pogo*	3
Vollenhovia	*emeryi*	-	Metazoa	Arthropoda	Insecta	Hymenoptera	*pogo*	3
Vombatus	*ursinus*	-	Metazoa	Chordata	Mammalia	Diprotodontia	*pogo*	8
Vulpes	*vulpes*	-	Metazoa	Chordata	Mammalia	Carnivora	*pogo*	5
Wasmannia	*auropunctata*	-	Metazoa	Arthropoda	Insecta	Hymenoptera	*pogo*	1
Xenopus	*laevis*	-	Metazoa	Chordata	Amphibia	Anura	*pogo*	1
Xenopus	*tropicalis*	-	Metazoa	Chordata	Amphibia	Anura	*pogo*	1
Xiphophorus	*maculatus*	-	Metazoa	Chordata	Actinopteri	Cyprinodontiformes	*pogo*	1
Yarrowia	*lipolytica*	-	Fungi	Ascomycota	Saccharomycetes	Saccharomycetales	*pogo*	2
Zalophus	*californianus*	-	Metazoa	Chordata	Mammalia	Carnivora	*pogo*	4
Zeugodacus	*cucurbitae*	-	Metazoa	Arthropoda	Insecta	Diptera	*pogo*	3
Zonotrichia	*albicollis*	-	Metazoa	Chordata	Aves	Passeriformes	*pogo*	1
Zootermopsis	*nevadensis*	-	Metazoa	Arthropoda	Insecta	Blattodea	*pogo*	8
Zostera	*marina*	-	Plantae	Streptophyta	Liliopsida	Alismatales	*pogo*	1
