# Supplementary figures and images for: Phylogenetic analysis of the Tc1/mariner superfamily reveals the unexplored diversity of pogo-like elements
Source: Mob DNA. 2020 Jun 29;11:21. doi: 10.1186/s13100-020-00212-0 (PMC7325037; doi:10.1186/s13100-020-00212-0)

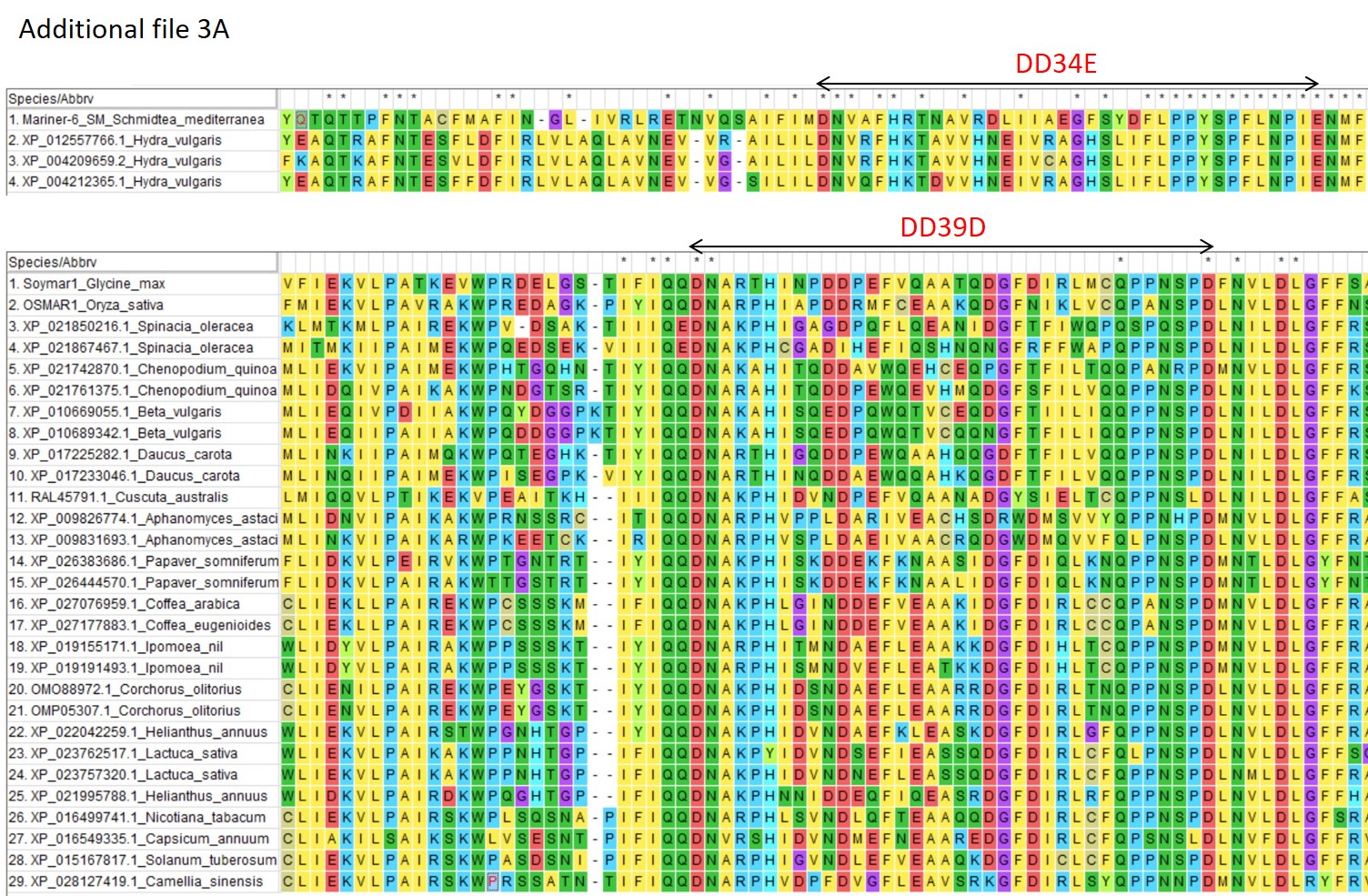

Supplement: Supplementary file 3 — Additional file 3. Alignment text files showing the DDD/E structure of each clade showing a conserved amino acid residues number between the second D and the third D or the E of the transposase domain. A) DDD/E alignment caption for HvSm and PlantMar. B) DDD alignment caption for TIGD1–4. C) DDD alignment caption for TIGD5–7. [file 13100_2020_212_MOESM3_ESM.zip › Additional-file3A_HvSm_PlantMar_DDD-Edomain.jpg]

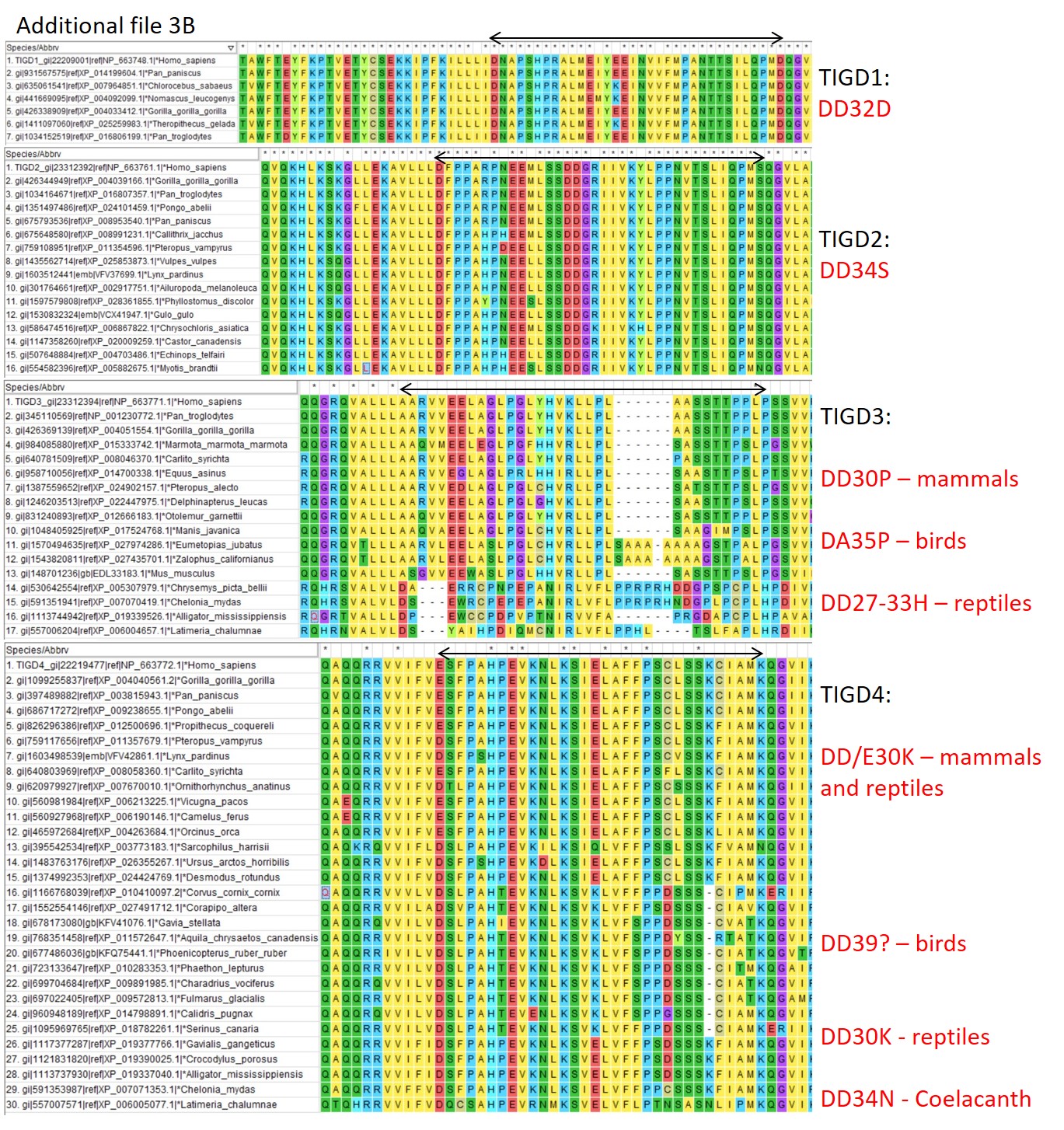

Supplement: Supplementary file 3 — Additional file 3. Alignment text files showing the DDD/E structure of each clade showing a conserved amino acid residues number between the second D and the third D or the E of the transposase domain. A) DDD/E alignment caption for HvSm and PlantMar. B) DDD alignment caption for TIGD1–4. C) DDD alignment caption for TIGD5–7. [file 13100_2020_212_MOESM3_ESM.zip › Additional-file3B_TIGD1-4_DDDdomain.jpg]

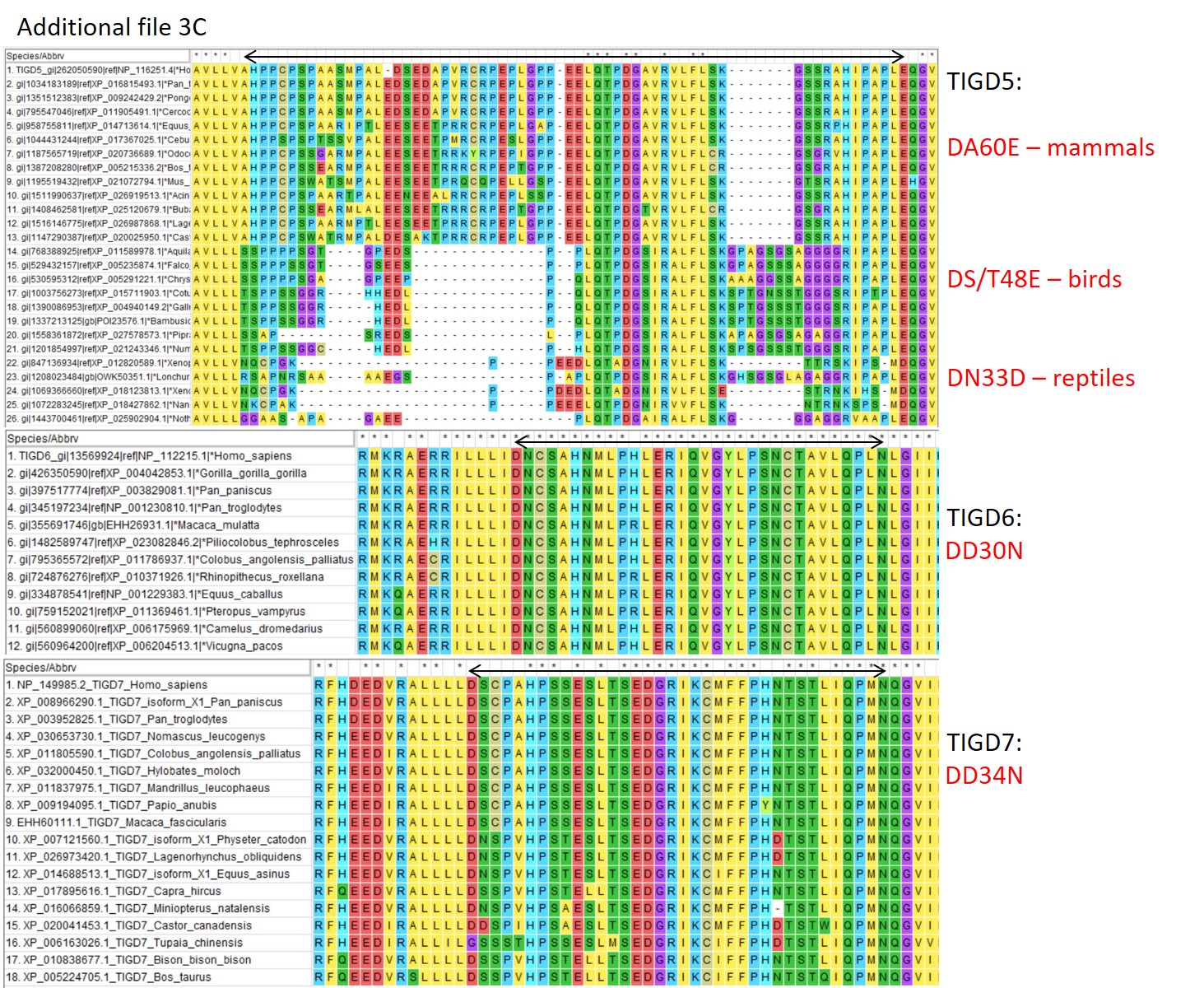

Supplement: Supplementary file 3 — Additional file 3. Alignment text files showing the DDD/E structure of each clade showing a conserved amino acid residues number between the second D and the third D or the E of the transposase domain. A) DDD/E alignment caption for HvSm and PlantMar. B) DDD alignment caption for TIGD1–4. C) DDD alignment caption for TIGD5–7. [file 13100_2020_212_MOESM3_ESM.zip › Additional-file3C_TIGD5-7_DDDdomain.jpg]
