# Supplementary material for: Phylogenetic analysis of the Tc1/mariner superfamily reveals the unexplored diversity of pogo-like elements
Source: Mob DNA. 2020 Jun 29;11:21. doi: 10.1186/s13100-020-00212-0 (PMC7325037; doi:10.1186/s13100-020-00212-0)
Supplement: Supplementary file 6 — Additional files 6 to 12. Conserved locations of TIGD1 to TIGD7 in host vertebrate species and information about the upstream and downstream genes flanking them, retrieved from Ensembl [54]. Negative numbers indicate that the considered gene is upstream of the TIGD element. [file 13100_2020_212_MOESM6_ESM.zip › 13100_2020_212_MOESM8_ESM.pdf]

Species	TIGD3 Ensembl name	Scaffold or chromosome	Pos. TIGD3 1 (bp)	Pos. TIGD3 2 (bp)	Sense	Dist. DPF2 1 (bp)	Dist. DPF2 2 (bp)	DPF2 Ensembl name	Scaffold or chromosome	Pos. DPF2 1 (bp)	Pos. DPF2 2 (bp)	Sense	DistSLC1	DistSLC2	SLC25A45 Ensembl name	Scaffold or chromosome	Pos. SLC25A45 1 (bp)	Pos. SLC25A45 2 (bp)	Sense
Abingdon island giant tortoise_Chelonoidis abingdonii	ENSCABG00000021472	PKMU01000757.1	656378	657817	1	-3128149	-3157936	ENSCABG00000001614	PKMU01005734.1	3784527	3815753	-1	14873	10637	ENSCABG00000021471	PKMU01000757.1	641505	647180	1
Agassiz's desert tortoise_Gopherus agassizii	ENSGAGG00000017236	PPEB01009481.1	39300	40739	-1	-118354	-161816	ENSGAGG00000014005	PPEB01001615.1	157654	202555	-1	-9651	-12531	ENSGAGG00000017237	PPEB01009481.1	48951	53270	-1
Algerian mouse_Mus spretus	MGP_SPRETEiJ_G0023488	19	2824469	2827427	-1	-5224	-18567	MGP_SPRETEiJ_G0023489	19	2829693	2845994	-1	17591	12541	MGP_SPRETEiJ_G0023487	19	2806878	2814886	1
Alpine marmot_Marmota marmota marmota	ENSMMMG00000010038	CZRN01000004.1	67527110	67528525	1	18374	4309	ENSMMMG00000010009	CZRN01000004.1	67508736	67524216	1	-16237	-19691	ENSMMMG00000010041	CZRN01000004.1	67543347	67548216	-1
American beaver_Castor canadensis	ENSCCNG00000024854	MTKA01001235.1	2853691	2855106	-1	-4339	-17496	ENSCCNG00000024857	MTKA01001235.1	2858030	2872602	-1	12678	9542	ENSCCNG00000024841	MTKA01001235.1	2841013	2845564	1
American bison_Bison bison bison	ENSBBBG00000019013	KN264804.1	1643325	1644740	1	17032	6882	ENSBBBG00000019075	KN264804.1	1626293	1637858	1	-14270	-17417	ENSBBBG00000004847	KN264804.1	1657595	1662157	-1
American black bear_Ursus americanus	ENSUAMG00000000430	LZNR01004112.1	49505	50926	-1	-4932	-15176	ENSUAMG00000000449	LZNR01004112.1	54437	66102	-1	16151	11534	ENSUAMG00000000421	LZNR01004112.1	33354	39392	1
American mink_Neovison vison	ENSNVIG00000016755	FNWR01000105.1	4352938	4354362	-1	-5547	-18285	ENSNVIG00000016764	FNWR01000105.1	4358485	4372647	-1	12811	9574	ENSNVIG00000016662	FNWR01000105.1	4340127	4344788	1
Angola colobus_Colobus angolensis palliatus	ENSCANG00000011603	KN980607.1	4750690	4752105	-1	-4273	-24833	ENSCANG00000043074	KN980607.1	4754963	4776938	-1	37088	30564	ENSCANG00000042819	KN980607.1	4713602	4721541	1
Arabian camel_Camelus dromedarius	ENSCDRG00005009809	10	7166284	7167699	-1	-4298	-16226	ENSCDRG00005009844	10	7170582	7183925	-1	14546	8992	ENSCDRG00005009601	10	7151738	7158707	1
Arctic ground squirrel_Urocitellus parryii	ENSUPAG00010009889	QVIC01000173.1	3230052	3231467	-1	-4511	-18146	ENSUPAG00010009890	QVIC01000173.1	3234563	3249613	-1	20229	-21	ENSUPAG00010009854	QVIC01000173.1	3209823	3231488	1
Armadillo_Dasypus novemcinctus	ENSDNOG00000004634	JH577683.1	1022015	1023418	1	17557	2518	ENSDNOG00000042153	JH577683.1	1004458	1020900	1	-21122	-28677	ENSDNOG00000047325	JH577683.1	1043137	1052095	-1
Black snub-nosed monkey_Rhinopithecus bieti	ENSRBIG00000030267	MCGX01000834.1	1439678	1441093	1	23414	4508	ENSRBIG00000027097	MCGX01000834.1	1416264	1436585	1	-16935	-25001	ENSRBIG00000031082	MCGX01000834.1	1456613	1466094	-1
Bolivian squirrel monkey_Saimiri boliviensis boliviensis	ENSSBOG00000019092	JH378199.1	1697728	1699143	1	22563	4105	ENSSBOG00000029354	JH378199.1	1675165	1695038	1	-16039	-22050	ENSSBOG00000010340	JH378199.1	1713767	1721193	-1
Bonobo_Pan paniscus	ENSPPAG00000001675	11	64058807	64060222	1	21699	4495	ENSPPAG00000043218	11	64037108	64055727	1	-16868	-22528	ENSPPAG00000035170	11	64075675	64082750	-1
Bushbaby_Otolemur garnettii	ENSOGAG00000027212	GL873659.1	2254916	2256349	1	26053	6949	ENSOGAG00000014697	GL873659.1	2228863	2249400	1	-14234	-18238	ENSOGAG00000014703	GL873659.1	2269150	2274587	-1
Canada lynx_Lynx canadensis	ENSLCNG00005012902	D1	108788456	108789871	1	17447	4627	ENSLCNG00005012875	D1	108771009	108785244	1	-8339	-13903	ENSLCNG00005012925	D1	108796795	108803774	-1
Cat_Felis catus	ENSFCAG00000025695	D1	110521556	110522971	1	16905	4607	ENSFCAG00000023858	D1	110504651	110518364	1	-9865	-15106	ENSFCAG00000034268	D1	110531421	110538077	-1
Chacoan peccary_Catagonus wagneri	ENSCWAG00000013059	PVHT021183668.1	3644631	3646046	-1	-5572	-20066	ENSCWAG00000013089	PVHT021183668.1	3650203	3666112	-1	18395	14328	ENSCWAG00000012825	PVHT021183668.1	3626236	3631718	1
Chimpanzee_Pan troglodytes	ENSPTRG00000003873	11	65420085	65421500	1	22079	4241	ENSPTRG00000003872	11	65398006	65417259	1	-18121	-24962	ENSPTRG00000003874	11	65438206	65446462	-1
Chinese hamster CHOK1GS_Cricetulus griseus	ENSCGRG00001010362	scaffold_9	41661388	41662800	-1	-4991	-19530	ENSCGRG00001020101	scaffold_9	41666379	41682330	-1	12645	7580	ENSCGRG00001011502	scaffold_9	41648743	41655220	1
Chinese hamster CriGri_Cricetulus griseus	ENSCGRG00000018629	JH003119.1	31206	32618	-1	-2630	-19292	ENSCGRG00000002327	JH003119.1	33836	51910	-1	13984	6755	ENSCGRG00000001623	JH003119.1	17222	25863	1
Chinese hamster PICR_Cricetulus griseus	ENSCGRG00015014670	RAZU01000139.1	2770900	2772312	1	19389	6550	ENSCGRG00015014613	RAZU01000139.1	2751511	2765762	1	-6881	-12648	ENSCGRG00015014675	RAZU01000139.1	2777781	2784960	-1
Common wombat_Vombatus ursinus	ENSVURG00010002040	UNPS02011369.1	5399795	5401222	-1	-1,8E+07	-1,8E+07	ENSVURG00010018078	UNPS02014945.1	23114047	23115831	-1	15510	4032	ENSVURG00010001921	UNPS02011369.1	5384285	5397190	1
Cow_Bos taurus	ENSBTAG00000003079	29	43555590	43557005	1	16862	5489	ENSBTAG00000003077	29	43538728	43551516	1	-13619	-17829	ENSBTAG00000008568	29	43569209	43574834	-1
Crab-eating macaque_Macaca fascicularis	ENSMFAG00000023252	14	9159462	9160877	-1	-18137	-35405	ENSMFAG00000000560	14	9177599	9196282	-1	32432	25807	ENSMFAG00000045518	14	9127030	9135070	1
Damara mole rat_Fukomys damarensis	ENSFDAG00000010778	KN122776.1	6296002	6298498	-1	-3013	-16388	ENSFDAG00000020975	KN122776.1	6299015	6314886	-1	16678	14014	ENSFDAG00000010168	KN122776.1	6279324	6284484	1
Degu_Octodon degus	ENSODEG00000020531	JH651533.1	23059768	23061183	-1	-19529	-33433	ENSODEG00000017997	JH651533.1	23079297	23094616	-1	32926	20150	ENSODEG00000017936	JH651533.1	23026842	23041033	1
Dingo_Canis lupus dingo	ENSCAFG00020014976	QKWQ01000195.1	4075043	4076461	1	18012	4810	ENSCAFG00020014969	QKWQ01000195.1	4057031	4071651	1	-13789	-17372	ENSCAFG00020014978	QKWQ01000195.1	4088832	4093833	-1
Dog - Basenji_Canis lupus familiaris	ENSCAFG00030010539	18	4097754	4099172	1	17899	4810	ENSCAFG00030010337	18	4079855	4094362	1	-13724	-17348	ENSCAFG00030010570	18	4111478	4116520	-1
Dog - Great Dane_Canis lupus familiaris	ENSCAFG00040019230	18	51965272	51966690	-1	-6031	-17899	ENSCAFG00040019263	18	51971303	51984589	-1	17214	13577	ENSCAFG00040019124	18	51948058	51953113	1
Dog_Canis lupus familiaris	ENSCAFG00000013628	18	51828247	51829665	-1	-4823	-17422	ENSCAFG00000013654	18	51833070	51847087	-1	17208	13571	ENSCAFG00000028559	18	51811039	51816094	1
Donkey_Equus asinus asinus	ENSEASG00005017947	PSZQ01004146.1	32381	33796	-1	-3622	-14400	ENSEASG00005017960	PSZQ01004146.1	36003	48196	-1	13976	10133	ENSEASG00005017943	PSZQ01004146.1	18405	23663	1
Drill_Mandrillus leucophaeus	ENSMLEG00000002444	KN976557.1	4349466	4350881	1	20726	5159	ENSMLEG00000034766	KN976557.1	4328740	4345722	1	-33790	-39330	ENSMLEG00000030963	KN976557.1	4383256	4390211	-1
Elephant_Loxodonta africana	ENSLAFG00000032647	scaffold_71	4129477	4130892	-1	-5961	-15956	ENSLAFG00000016450	scaffold_71	4135438	4146848	-1	19960	17452	ENSLAFG00000006845	scaffold_71	4109517	4113440	1
Ferret_Mustela putorius furo	ENSMPUG00000019988	GL897050.1	801778	803202	-1	-3212	-18168	ENSMPUG00000011987	GL897050.1	804990	821370	-1	13939	9793	ENSMPUG00000011980	GL897050.1	787839	793409	1
Gelada_Theropithecus gelada	ENSTGEG00000009327	14	9254515	9255930	-1	-4237	-23708	ENSTGEG00000009427	14	9258752	9279638	-1	31407	24814	ENSTGEG00000009285	14	9223108	9231116	-1
Goat_Capra hircus	ENSCHIG00000006241	29	43974616	43976040	1	16937	6800	ENSCHIG00000011852	29	43957679	43969240	1	-13972	-17152	ENSCHIG00000015690	29	43988588	43993192	-1
Golden Hamster_Mesocricetus auratus	ENSMAUG00000016982	KB708127.1	51263036	51265931	1	19276	5647	ENSMAUG00000015922	KB708127.1	51243760	51260284	1	-7606	-11715	ENSMAUG00000014882	KB708127.1	51270642	51277646	-1
Golden snub-nosed monkey_Rhinopithecus roxellana	ENSRROG00000024539	KN295605.1	206779	208194	-1	-3732	-23585	ENSRROG00000036505	KN295605.1	210511	231779	-1	24744	18314	ENSRROG00000021175	KN295605.1	182035	189880	1
Gorilla_Gorilla gorilla gorilla	ENSGGOG00000014762	11	64551721	64553136	1	21431	4243	ENSGGOG00000026412	11	64530290	64548893	1	-24203	-30291	ENSGGOG00000014767	11	64575924	64583427	-1
Greater bamboo lemur_Prolemur simus	ENSPSMG00000023634	MPIZ01000746.1	569868	571283	-1	-6000	-17924	ENSPSMG00000000228	MPIZ01000746.1	575868	589207	-1	15598	12421	ENSPSMG00000023627	MPIZ01000746.1	554270	558862	1
Greater horseshoe bat_Rhinolophus ferrumequinum	ENSRFEG00010004495	11	6312581	6313996	-1	-4260	-18103	ENSRFEG00010004526	11	6316841	6332099	-1	14859	10229	ENSRFEG00010004466	11	6297722	6303767	1
Guinea Pig_Cavia porcellus	ENSCPOG00000009317	DS562897.1	6085152	6087974	1	16041	4084	ENSCPOG00000021560	DS562897.1	6069111	6083890	1	-11733	-19178	ENSCPOG00000010217	DS562897.1	6096885	6107152	-1
Horse_Equus caballus	ENSECAG00000006896	12	29112607	29114022	1	16904	4834	ENSECAG00000001650	12	29095703	29109188	1	-10098	-13912	ENSECAG00000006906	12	29122705	29127934	-1
Hybrid - Bos Indicus_Bos indicus x Bos taurus	ENSBIXG00005015667	29	7583423	7609726	-1	-29825	-16740	ENSBIXG00005015594	29	7613248	7626466	-1	-7619	12872	ENSBIXG00005015642	29	7591042	7596854	1
Hybrid - Bos Taurus_Bos indicus x Bos taurus	ENSBIXG00000009632	29	7589116	7590531	-1	-4937	-16740	ENSBIXG00000009622	29	7594053	7607271	-1	17263	12868	ENSBIXG00000009639	29	7571853	7577663	1
Kangaroo rat_Dipodomys ordii	ENSDORG00000029849	KN672400.1	4870130	4871545	-1	-5881	-24595	ENSDORG00000011600	KN672400.1	4876011	4896140	-1	9556	4785	ENSDORG00000023675	KN672400.1	4860574	4866760	1
Koala_Phascolarctos cinereus	ENSPCIG00000029216	MSTS01000180.1	3588138	3589553	1	16134	6147	ENSPCIG00000029217	MSTS01000180.1	3572004	3583406	1	-889	-15314	ENSPCIG00000029215	MSTS01000180.1	3589027	3604867	-1
Leopard_Panthera pardus	ENSPPRG00000022171	KV860336.1	7143757	7145172	1	17285	4563	ENSPPRG00000014643	KV860336.1	7126472	7140609	1	-13017	-17183	ENSPPRG00000013551	KV860336.1	7156774	7162355	-1
Lesser Egyptian jerboa_Jaculus jaculus	ENSJJAG00000011195	JH725456.1	3480233	3481648	1	31071	6476	ENSJJAG00000018564	JH725456.1	3449162	3475172	1	-17866	-23399	ENSJJAG00000017178	JH725456.1	3498099	3505047	-1
Long-tailed chinchilla_Chinchilla lanigera	ENSCLAG00000016665	JH721882.1	19880777	19883567	-1	-11412	-27422	ENSCLAG00000016884	JH721882.1	19892189	19910989	-1	26069	17029	ENSCLAG00000016505	JH721882.1	19854708	19866538	1
Meerkat_Suricata suricatta	ENSSSUG00005015248	11	6395464	6396882	-1	-4613	-17103	ENSSSUG00005015296	11	6400077	6413985	-1	15602	10985	ENSSSUG00005015093	11	6379862	6385897	1
Microbat_Myotis lucifugus	ENSMLUG00000003893	GL430329	479475	480917	1	12752	5992	ENSMLUG00000003890	GL430329	466723	474925	1	-10949	-14196	ENSMLUG00000003897	GL430329	490424	495113	-1
Mongolian gerbil_Meriones unguiculatus	ENSMUGG00000020610	NHTI01000219.1	634782	636194	1	19941	5500	ENSMUGG00000020560	NHTI01000219.1	614841	630694	1	-6810	-12841	ENSMUGG00000020613	NHTI01000219.1	641592	649035	-1
Mouse_Mus musculus	ENSMUSG00000044390	19	5891138	5894149	-1	-5378	-18861	ENSMUSG00000024826	19	5896516	5913010	-1	13330	8271	ENSMUSG00000024818	19	5877808	5885878	1
Naked mole-rat female_Heterocephalus glaber	ENSHGLG00000008128	JH602080.1	21026887	21029559	-1	-2628	-20336	ENSHGLG00000010867	JH602080.1	21029515	21049895	-1	18543	14827	ENSHGLG00000007151	JH602080.1	21008344	21014732	1
Olive baboon_Papio anubis	ENSPANG00000006957	14	8745257	8746672	-1	-2484	-22130	ENSPANG00000004688	14	8747741	8768802	-1	27180	22867	ENSPANG00000004415	14	8718077	8723805	1
Orangutan_Pongo abelii	ENSPPYG00000003068	11	10560857	10562272	-1	-3238	-23414	ENSPPYG00000003069	11	10564095	10585686	-1	26912	20585	ENSPPYG00000003067	11	10533945	10541687	1
Painted turtle_Chrysemys picta bellii	ENSCPBG00000023755	KK088199.1	7066593	7068035	1	1029361	870988	ENSCPBG00000023614	KK088199.1	6037232	6197047	-1	17089	8574	ENSCPBG00000023754	KK088199.1	7049504	7059461	1
Pig-tailed macaque_Macaca nemestrina	ENSMNEG00000028001	KQ007745.1	4030421	4031836	1	22725	2655	ENSMNEG00000032739	KQ007745.1	4007696	4029181	1	-16854	-25797	ENSMNEG00000042519	KQ007745.1	4047275	4057633	-1
Pig_Sus scrofa	ENSSSCG00000012994	2	6865374	6866789	-1	-4957	-19678	ENSSSCG00000012995	2	6870331	6886467	-1	13769	8909	ENSSSCG00000012993	2	6851605	6857880	1
Prairie vole_Microtus ochrogaster	ENSMOCG00000010179	8	40669695	40671107	-1	-5808	-18563	ENSMOCG00000021188	8	40675503	40689670	-1	14032	8340	ENSMOCG00000002439	8	40655663	40662767	1
Rat_Rattus norvegicus	ENSRNOG00000023318	1	221248910	221251329	-1	-4837	-17713	ENSRNOG00000020892	1	221253747	221269042	-1	12137	6766	ENSRNOG00000030616	1	221236773	221244563	1
Red fox_Vulpes vulpes	ENSVVUG00000027179	NBDQ01000050.1	10445636	10447054	-1	-5984	-17948	ENSVVUG00000027189	NBDQ01000050.1	10451620	10465002	-1	17690	13503	ENSVVUG00000027174	NBDQ01000050.1	10427946	10433551	1
Ryukyu mouse_Mus caroli	MGP_CAROLIEiJ_G0022574	19	2643843	2646814	-1	-5328	-17943	MGP_CAROLIEiJ_G0022575	19	2649171	2664757	-1	13123	8766	MGP_CAROLIEiJ_G0022573	19	2630720	2638048	1
Shrew mouse_Mus pahari	MGP_PahariEiJ_G0014071	1	117283304	117286101	-1	-5562	-18526	MGP_PahariEiJ_G0014072	1	117288866	117304627	-1	14906	9436	MGP_PahariEiJ_G0014070	1	117268398	117276665	1
Shrew_Sorex araneus	ENSSARG00000009391	GeneScaffold_5844	292	12353	1	-1588	556	ENSSARG00000005031	GeneScaffold_3041	1880	11797	1	-6894	4462	ENSSARG00000009400	GeneScaffold_5844	7186	7891	-1
Siberian musk deer_Moschus moschiferus	ENSMMSG00000019677	PVHU021072430.1	420231	421646	-1	-5551	-16933	ENSMMSG00000019700	PVHU021072430.1	425782	438579	-1	17262	14065	ENSMMSG00000019423	PVHU021072430.1	402969	407581	1
Sooty mangabey_Cercocebus atys	ENSCATG00000005263	KQ012441.1	9035141	9036556	-1	-4265	-22192	ENSCATG00000044255	KQ012441.1	9039406	9058748	-1	27644	20721	ENSCATG00000008120	KQ012441.1	9007497	9015835	1
Sperm whale_Physeter catodon	ENSPCTG00005017052	ML160719.1	77639	79357	1	17246	4868	ENSPCTG00005016883	ML160719.1	60393	74489	1	-13727	-17124	ENSPCTG00005017097	ML160719.1	91366	96481	-1
Squirrel_Ictidomys tridecemlineatus	ENSSTOG00000024637	JH393409.1	4058692	4060107	-1	-4072	-18121	ENSSTOG00000003746	JH393409.1	4062764	4078228	-1	20812	16841	ENSSTOG00000000609	JH393409.1	4037880	4043266	1
Steppe mouse_Mus spicilegus	ENSMSIG00000030313	QGOO01037275.1	982048	985094	1	22682	6101	ENSMSIG00000030307	QGOO01037275.1	959366	978993	1	-8200	-12336	ENSMSIG00000030315	QGOO01037275.1	990248	997430	-1
Tasmanian devil_Sarcophilus harrisii	ENSSHAG00000003566	GL864985.1	67297	68712	1	13192	7070	ENSSHAG00000003172	GL864985.1	54105	61642	1	-2300	-15589	ENSSHAG00000003643	GL864985.1	69597	84301	-1
Three-toed box turtle_Terrapene carolina triunguis	ENSTMTG00000003902	PRFB02000090.1	31083039	31084481	1	933520	894722	ENSTMTG00000002493	PRFB02000090.1	30149519	30189759	-1	14542	10713	ENSTMTG00000003882	PRFB02000090.1	31068497	31073768	1
Ugandan red Colobus_Piliocolobus tephrosceles	ENSPTEG00000024133	PDMG02000207.1	4197112	4198527	1	22967	4266	ENSPTEG00000024101	PDMG02000207.1	4174145	4194261	1	-18956	-24292	ENSPTEG00000024247	PDMG02000207.1	4216068	4222819	-1
Upper Galilee mountains blind mole rat_Nannospalax galili	ENSNGAG00000015391	KL205152.1	301543	304247	1	18553	6238	ENSNGAG00000014669	KL205152.1	282990	298009	1	-11122	-15323	ENSNGAG00000015505	KL205152.1	312665	319570	-1
Vervet-AGM_Chlorocebus sabaeus	ENSCSAG00000019171	1	8884276	8885691	-1	-2434	-22831	ENSCSAG00000007767	1	8886710	8908522	-1	27299	20333	ENSCSAG00000007775	1	8856977	8865358	1
Wild yak_Bos mutus	ENSBMUG00000002328	JH880973.1	782835	784250	-1	-5491	-12754	ENSBMUG00000002373	JH880973.1	788326	797004	-1	17288	14077	ENSBMUG00000002297	JH880973.1	765547	770173	1
