# Supplementary material for: Phylogenetic analysis of the Tc1/mariner superfamily reveals the unexplored diversity of pogo-like elements
Source: Mob DNA. 2020 Jun 29;11:21. doi: 10.1186/s13100-020-00212-0 (PMC7325037; doi:10.1186/s13100-020-00212-0)
Supplement: Supplementary file 6 — Additional files 6 to 12. Conserved locations of TIGD1 to TIGD7 in host vertebrate species and information about the upstream and downstream genes flanking them, retrieved from Ensembl [54]. Negative numbers indicate that the considered gene is upstream of the TIGD element. [file 13100_2020_212_MOESM6_ESM.zip › 13100_2020_212_MOESM7_ESM.pdf]

| Species | TIGD2 Ensembl name | Scaffold or chromosome | Position 1 (bp) | Position 2 (bp) | Sense | Dist. FAM13A 1 (bp) | Dist. FAM13A 2 (bp) | FAM13A Ensembl name | Scaffold or chromosome | Pos. FAM13A 1 (bp) | Pos. FAM13A 2 (bp) | Sense | Dist. GPRIN3 1 (bp) | Dist. GPRIN3 2 (bp) | GPRIN3 Ensembl name | Scaffold or chromosome | Position GPRIN3 1 (bp) | Position GPRin3 2 (bp) | Sense |
|---|---|---|---|---|---|---|---|---|---|---|---|---|---|---|---|---|---|---|---|
| Human  Homo sapien | ENSG00000180346 | 4 | 90033968 | 90036050 | 1 | 386862 | 3501 | ENSG00000138640 | 4 | 89647106 | 90032549 | -1 | -123569 | -193111 | ENSG00000185477 | 4 | 90157537 | 90229161 | -1 |
| Algerian mouse  Mus spretus | MGP SPRETEiJ G0029454 | 6 | 57217403 | 57220449 | 1 | 274293 | 185790 | MGP SPRETEiJ G0029453 | 6 | 56943110 | 57034659 | -1 | -148586 | -228560 | MGP SPRETEiJ G0029455 | 6 | 57365989 | 57449009 | -1 |
| American bison  Bison bison bison | ENSBBBG00000011122 | KN265160.1 | 5303164 | 5304741 | -1 | -29262 | -338291 | ENSBBBG00000011095 | KN265160.1 | 5332426 | 5643032 | 1 | 129554 | 128792 | ENSBBBG00000011131 | KN265160.1 | 5173610 | 5175949 | 1 |
| American mink  Neovison vison | ENSNVIG00000022434 | FNWR01000174.1 | 1474838 | 1476415 | 1 | 353874 | 29806 | ENSNVIG00000022199 | FNWR01000174.1 | 1120964 | 1446609 | -1 | -127132 | -127906 | ENSNVIG00000022443 | FNWR01000174.1 | 1601970 | 1604321 | -1 |
| Arabian camel  Camelus dromedarius | ENSCDRG00005001330 | 2 | 81719637 | 81721214 | 1 | 287694 | 22884 | ENSCDRG00005000724 | 2 | 81431943 | 81698330 | -1 | -110446 | -111217 | ENSCDRG00005001337 | 2 | 81830083 | 81832431 | -1 |
| Arctic ground squirrel  Urocitellus parryii | ENSUPAG00010010369 | QVIC01000314.1 | 773211 | 774791 | 1 | 332453 | 28428 | ENSUPAG00010010271 | QVIC01000314.1 | 440758 | 746363 | -1 | -157277 | -157994 | ENSUPAG00010010391 | QVIC01000314.1 | 930488 | 932785 | -1 |
| Bonobo  Pan paniscus | ENSPPAG00000037756 | 4 | 92122501 | 92127090 | 1 | 382548 | 59675 | ENSPPAG00000001057 | 4 | 91739953 | 92067415 | -1 | -134624 | -132365 | ENSPPAG00000020409 | 4 | 92257125 | 92259455 | -1 |
| Bushbaby  Otolemur garnettii | ENSOGAG00000032052 | GL873558.1 | 1660475 | 1662052 | -1 | -60121 | -300211 | ENSOGAG00000003314 | GL873558.1 | 1720596 | 1962263 | 1 | 144738 | 144015 | ENSOGAG00000003968 | GL873558.1 | 1515737 | 1518037 | 1 |
| Canada lynx  Lynx canadensis | ENSLCNG00005001329 | B1 | 132333971 | 132335548 | -1 | -3101 | -360993 | ENSLCNG00005001422 | B1 | 132337072 | 1,33E+08 | 1 | 145240 | 144493 | ENSLCNG00005001319 | B1 | 132188731 | 132191055 | 1 |
| Chacoan peccary  Catagonus wagneri | ENSCWAG00000016360 | PVHT020000886.1 | 5657361 | 5658938 | -1 | -26244 | -335021 | ENSCWAG00000016443 | PVHT020000886.1 | 5683605 | 5993959 | 1 | 121194 | 120420 | ENSCWAG00000016347 | PVHT020000886.1 | 5536167 | 5538518 | 1 |
| Chimpanzee  Pan troglodytes | ENSPTRG00000031142 | 4 | 91502750 | 91504327 | 1 | 380623 | 56799 | ENSPTRG00000016278 | 4 | 91122127 | 91447528 | -1 | -135627 | -136380 | ENSPTRG00000048747 | 4 | 91638377 | 91640707 | -1 |
| Chinese hamster CHOK1GS  Cricetulus griseus | ENSCGRG00001011090 | scaffold_35 | 17127110 | 17128687 | 1 | 285264 | 196844 | ENSCGRG00001018932 | scaffold 35 | 16841846 | 16931843 | -1 | 8117724 | 8117034 | ENSCGRG00001013859 | scaffold 44 | 9009386 | 9011653 | 1 |
| Chinese hamster PICR  Cricetulus griseus | ENSCGRG00015024758 | RAZU01000265.1 | 10097432 | 10099009 | 1 | 286765 | 195940 | ENSCGRG00015024687 | RAZU01000265.1 | 9810667 | 9903069 | -1 | 9950823 | 9950133 | ENSCGRG00015013031 | RAZU01000272.1 | 146609 | 148876 | -1 |
| Common wombat  Vombatus ursinus | ENSVURG00010021145 | UNPS02009975.1 | 3901185 | 3902777 | -1 | -62328 | -164796 | ENSVURG00010021156 | UNPS02009975.1 | 3963513 | 4067573 | 1 | 64577 | 64148 | ENSVURG00010021143 | UNPS02009975.1 | 3836608 | 3838629 | 1 |
| Cow  Bos taurus | ENSBTAG00000015265 | 6 | 35692061 | 35693638 | -1 | -29085 | -337402 | ENSBTAG00000011187 | 6 | 35721146 | 36031040 | 1 | 128408 | 127643 | ENSBTAG00000045966 | 6 | 35563653 | 35565995 | 1 |
| Degu  Octodon degus | ENSODEG00000018566 | JH651787.1 | 510847 | 512427 | -1 | -345291 | -407010 | ENSODEG00000000502 | JH651787.1 | 856138 | 919437 | 1 | 190953 | 190227 | ENSODEG00000003246 | JH651787.1 | 319894 | 322200 | 1 |
| Dog - Basenji  Canis lupus familiaris | ENSCAFG00030025102 | 32 | 27508391 | 27509968 | -1 | -29868 | -342627 | ENSCAFG00030025146 | 32 | 27538259 | 27852595 | 1 | 161297 | 160369 | ENSCAFG00030025071 | 32 | 27347094 | 27349599 | 1 |
| Dog - Great Dane  Canis lupus familiaris | ENSCAFG00040017705 | 32 | 12480244 | 12481821 | 1 | 341912 | 3512 | ENSCAFG00040016054 | 32 | 12138332 | 12478309 | -1 | -159783 | -160716 | ENSCAFG00040017757 | 32 | 12640027 | 12642537 | -1 |
| Dog  Canis lupus familiaris | ENSCAFG00000009844 | 32 | 12341225 | 12342802 | 1 | 343111 | 3999 | ENSCAFG00000009840 | 32 | 11998114 | 12338803 | -1 | -160352 | -161105 | ENSCAFG00000009859 | 32 | 12501577 | 12503907 | -1 |
| Donkey  Equus asinus asinus | ENSEASG00005012477 | PSZQ01005937.1 | 8519711 | 8521288 | -1 | -41041 | -309084 | ENSEASG00005012503 | PSZQ01005937.1 | 8560752 | 8830372 | 1 | 117741 | 116985 | ENSEASG00005012469 | PSZQ01005937.1 | 8401970 | 8404303 | 1 |
| Elephant  Loxodonta africana | ENSLAFG00000031840 | scaffold_30 | 6327181 | 6328485 | -1 | -352118 | -365741 | ENSLAFG00000006124 | scaffold 30 | 6679299 | 6694226 | 1 | 177164 | 176114 | ENSLAFG00000025610 | scaffold 30 | 6150017 | 6152371 | 1 |
| Ferret  Mustela putorius furo | ENSMPUG00000019104 | GL896950.1 | 9168335 | 9169912 | -1 | -29883 | -370860 | ENSMPUG00000002154 | GL896950.1 | 9198218 | 9540772 | 1 | 129096 | 128319 | ENSMPUG00000019096 | GL896950.1 | 9039239 | 9041593 | 1 |
| Gelada  Theropithecus gelada | ENSTGEG00000004877 | 5 | 52736580 | 52738157 | -1 | -57565 | -384966 | ENSTGEG00000005053 | 5 | 52794145 | 53123123 | 1 | 131203 | 130450 | ENSTGEG00000004860 | 5 | 52605377 | 52607707 | 1 |
| Gibbon  Nomascus leucogenys | ENSNLEG00000009788 | 9 | 74069174 | 74073641 | 1 | 394410 | 62796 | ENSNLEG00000009768 | 9 | 73674764 | 74010845 | -1 | -138579 | -136442 | ENSNLEG00000018567 | 9 | 74207753 | 74210083 | -1 |
| Goat  Capra hircus | ENSCHIG00000008747 | 6 | 36205904 | 36207478 | -1 | -27983 | -79705 | ENSCHIG00000024343 | 6 | 36233887 | 36287183 | 1 | 135536 | 134768 | ENSCHIG00000008090 | 6 | 36070368 | 36072710 | 1 |
| Golden Hamster  Mesocricetus auratus | ENSMAUG00000000071 | KB708185.1 | 1035028 | 1038108 | 1 | 291044 | 208076 | ENSMAUG00000019183 | KB708185.1 | 743984 | 830032 | -1 | -134400 | -200005 | ENSMAUG00000000174 | KB708185.1 | 1169428 | 1238113 | -1 |
| Gorilla  Gorilla gorilla gorilla | ENSGGOG00000026736 | 4 | 100301836 | 100303680 | 1 | 401590 | 60469 | ENSGGOG00000024484 | 4 | 99900246 | 1E+08 | -1 | -137707 | -138193 | ENSGGOG00000041041 | 4 | 100439543 | 100441873 | -1 |
| Greater bamboo lemur  Prolemur simus | ENSPSMG00000003741 | MPIZ01001751.1 | 475470 | 477047 | 1 | 225313 | 26044 | ENSPSMG00000002875 | MPIZ01001751.1 | 250157 | 451003 | -1 | -82571 | -83306 | ENSPSMG00000003754 | MPIZ01001751.1 | 558041 | 560353 | -1 |
| Greater horseshoe bat  Rhinolophus ferrumequinum | ENSRFEG00010004701 | 5 | 42910390 | 42911967 | 1 | 285827 | 3243 | ENSRFEG00010004628 | 5 | 42624563 | 42908724 | -1 | -86745 | -87516 | ENSRFEG00010004704 | 5 | 42997135 | 42999483 | -1 |
| Kangaroo rat  Dipodomys ordii | ENSDORG00000025654 | KN672468.1 | 3275018 | 3278207 | 1 | 197788 | 171099 | ENSDORG00000003574 | KN672468.1 | 3077230 | 3107108 | -1 | -57258 | -56429 | ENSDORG00000002588 | KN672468.1 | 3332276 | 3334636 | -1 |
| Lesser Egyptian jerboa  Jaculus jaculus | ENSJJAG00000002866 | JH725515.1 | 3660212 | 3661789 | -1 | -290710 | -368187 | ENSJJAG00000004621 | JH725515.1 | 3950922 | 4029976 | 1 | 215434 | 214786 | ENSJJAG00000010174 | JH725515.1 | 3444778 | 3447003 | 1 |
| Long-tailed chinchilla  Chinchilla lanigera | ENSCLAG00000005468 | JH721865.1 | 80872 | 84184 | 1 | -1,2E+07 | -1E+07 | ENSCLAG00000007217 | JH721934.1 | 11614830 | 11714369 | 1 | -173233 | -172230 | ENSCLAG00000017649 | JH721865.1 | 254105 | 256414 | -1 |
| Macaque  Macaca mulatta | ENSMMUG00000039305 | 5 | 87490364 | 87493706 | 1 | 377583 | 58041 | ENSMMUG00000010141 | 5 | 87112781 | 87435665 | -1 | -127883 | -189241 | ENSMMUG00000057606 | 5 | 87618247 | 87682947 | -1 |
| Marmoset  Callithrix jacchus | ENSCJAG00000031809 | NTIC01033278.1 | 78793815 | 78795392 | -1 | -59382 | -423185 | ENSCJAG00000006632 | NTIC01033278.1 | 78853197 | 79218577 | 1 | 144432 | 143679 | ENSCJAG00000022026 | NTIC01033278.1 | 78649383 | 78651713 | 1 |
| Meerkat  Suricata suricatta | ENSSSUG00005015730 | 1 | 124320858 | 124335084 | -1 | -14873 | -337273 | ENSSSUG00005015763 | 1 | 124335731 | 1,25E+08 | 1 | 119173 | 131066 | ENSSSUG00005015717 | 1 | 124201685 | 124204018 | 1 |
| Mongolian gerbil  Meriones unguiculatus | ENSMUGG00000003738 | NHTI01000800.1 | 452576 | 454159 | 1 | 286688 | 203508 | ENSMUGG00000003598 | NHTI01000800.1 | 165888 | 250651 | -1 | -73543 | -74239 | ENSMUGG00000003741 | NHTI01000800.1 | 526119 | 528398 | -1 |
| Mouse  Mus musculus | ENSMUSG00000049232 | 6 | 59208870 | 59212033 | 1 | 276780 | 187484 | ENSMUSG00000037709 | 6 | 58932090 | 59024549 | -1 | -138356 | -214261 | ENSMUSG00000045441 | 6 | 59347226 | 59426294 | -1 |
| Northern American deer mouse  Peromyscus maniculatus bairdii | ENSPEMG00000019700 | 3 | 77125040 | 77126617 | -1 | -209911 | -300269 | ENSPEMG00000010492 | 3 | 77334951 | 77426886 | 1 | 185281 | 184561 | ENSPEMG00000027579 | 3 | 76939759 | 76942056 | 1 |
| Orangutan  Pongo abelii | ENSPPYG00000014930 | 4 | 93038584 | 93040161 | 1 | 396375 | 64489 | ENSPPYG00000014928 | 4 | 92642209 | 92975672 | -1 | -139772 | -140543 | ENSPPYG00000014931 | 4 | 93178356 | 93180704 | -1 |
| Prairie vole  Microtus ochrogaster | ENSMOCG00000011023 | LG3 | 13032442 | 13035320 | -1 | -219608 | -303730 | ENSMOCG00000014628 | LG3 | 13252050 | 13339050 | 1 | 189189 | 128571 | ENSMOCG00000001332 | LG3 | 12843253 | 12906749 | 1 |
| Rat  Rattus norvegicus | ENSRNOG00000038449 | 4 | 89536647 | 89538227 | 1 | 352879 | 257005 | ENSRNOG00000007947 | 4 | 89183768 | 89281222 | -1 | -156633 | -157701 | ENSRNOG00000023657 | 4 | 89693280 | 89695928 | -1 |
| Red fox  Vulpes vulpes | ENSVVUG00000007747 | NBDQ01000028.1 | 2381125 | 2382702 | -1 | -29622 | -345967 | ENSVVUG00000007768 | NBDQ01000028.1 | 2410747 | 2728669 | 1 | 165066 | 164301 | ENSVVUG00000007735 | NBDQ01000028.1 | 2216059 | 2218401 | 1 |
| Ryukyu mouse  Mus caroli | MGP CAROLIEiJ G0028450 | 6 | 53303868 | 53307026 | 1 | 260971 | 174511 | MGP CAROLIEiJ G0028449 | 6 | 53042897 | 53132515 | -1 | -134107 | -212843 | MGP CAROLIEiJ G0028451 | 6 | 53437975 | 53519869 | -1 |
| Shrew mouse  Mus pahari | MGP PahariEiJ G0022191 | 2 | 77679483 | 77682678 | 1 | 270101 | 179997 | MGP PahariEiJ G0022190 | 2 | 77409382 | 77502681 | -1 | -128134 | -213369 | MGP PahariEiJ G0022192 | 2 | 77807617 | 77896047 | -1 |
| Siberian musk deer  Moschus moschiferus | ENSMMSG00000018674 | PVHU021072487.1 | 4572384 | 4573961 | 1 | 353637 | 26718 | ENSMMSG00000017996 | PVHU021072487.1 | 4218747 | 4547243 | -1 | -135338 | -136100 | ENSMMSG00000018693 | PVHU021072487.1 | 4707722 | 4710061 | -1 |
| Sperm whale  Physeter catodon | ENSPCTG00005002958 | 7 | 9909035 | 9910612 | 1 | 378683 | 24883 | ENSPCTG00005002764 | 7 | 9530352 | 9885729 | -1 | -140711 | -141488 | ENSPCTG00005002964 | 7 | 10049746 | 10052100 | -1 |
| Squirrel  Ictidomys tridecemlineatus | ENSSTOG00000028053 | JH393495.1 | 1501336 | 1505953 | 1 | 329752 | 4879 | ENSSTOG00000011695 | JH393495.1 | 1171584 | 1501074 | -1 | -154748 | -152431 | ENSSTOG00000002493 | JH393495.1 | 1656084 | 1658384 | -1 |
| Steppe mouse  Mus spicilegus | ENSMSIG00000010392 | QGOO01036810.1 | 580273 | 581850 | 1 | 273824 | 186373 | ENSMSIG00000010222 | QGOO01036810.1 | 306449 | 395477 | -1 | -132407 | -133121 | ENSMSIG00000010400 | QGOO01036810.1 | 71268 | 714971 | -1 |
| Ugandan red Colobus  Piliocolobus tephrosceles | ENSPTEG00000021961 | PDMG02000350.1 | 748151 | 749728 | 1 | 379480 | 58693 | ENSPTEG00000021912 | PDMG02000350.1 | 368671 | 691035 | -1 | -122736 | -123489 | ENSPTEG00000021995 | PDMG02000350.1 | 870887 | 873217 | -1 |
| Upper Galilee mountains blind mole rat  Nannospalax galili | ENSNGAG00000013979 | KL202846.1 | 8155426 | 8157000 | -1 | -229718 | -330865 | ENSNGAG00000016431 | KL202846.1 | 8385144 | 8487865 | 1 | 378409 | 377692 | ENSNGAG00000000029 | KL202846.1 | 7777017 | 7779308 | -1 |
| Vervet-AGM  Chlorocebus sabaeus | ENSCSAG00000000410 | 7 | 37460603 | 37462180 | 1 | 375011 | 55267 | ENSCSAG00000004234 | 7 | 37085592 | 37406913 | -1 | -130233 | -130986 | ENSCSAG00000000409 | 7 | 37590836 | 37593166 | -1 |
