# Supplementary material for: Phylogenetic analysis of the Tc1/mariner superfamily reveals the unexplored diversity of pogo-like elements
Source: Mob DNA. 2020 Jun 29;11:21. doi: 10.1186/s13100-020-00212-0 (PMC7325037; doi:10.1186/s13100-020-00212-0)
Supplement: Supplementary file 6 — Additional files 6 to 12. Conserved locations of TIGD1 to TIGD7 in host vertebrate species and information about the upstream and downstream genes flanking them, retrieved from Ensembl [54]. Negative numbers indicate that the considered gene is upstream of the TIGD element. [file 13100_2020_212_MOESM6_ESM.zip › 13100_2020_212_MOESM6_ESM.pdf]

Species	Location	Position 1 (bp)	Position 2 (bp)	Sense	Dist. CHRNG 1 (bp)	Dist. CHRNG 2 (bp)	Loc_CHRNG	Pos. CHRNG 1 (bp)	Pos. CHRNG 2 (bp)	Sense CHRNG	Dist. EIF4E2 1 (bp)	Dist. EIF4E2 2 (bp)	EIF4E2 name and location	Pos. EIF4E2 1 (bp)	Pos. EIF4E2 2 (bp)	Sense EIF4E2
Bolivian squirrel monkey_Saimiri boliviensis boliviensis	Nothing annotated_JH378191	606241	608013	-1	10876	3499	CHRNG _ENSSBOG00000031334_JH378191.1	595365	604514	1	-2461	-35154	EIF4E2 _ENSSBOG00000007474_JH378191.1	608702	643167	1
Bonobo_Pan paniscus	TIGD1 _ENSPPAG00000025445_2B	238698311	238700086	-1	8376	3560	CHRNG _ENSPPAG00000043480_2B	238689935	238696526	1	-2479	-33568	EIF4E2 _ENSPPAG00000034717_2B	238700790	238733654	1
Bushbaby_Otolemur garnettii	TIGD1 _ENSOGAG00000030017_GL873532.1	1143258	1145021	1	-3446	-7272	CHRNG _ENSOGAG00000032889_GL873532.1	1146704	1152293	-1	19884	10208	ENSOGAG00000009155_GL873532.1	1123374	1134813	-1
Capuchin_Cebus capucinus imitator	ENSCCAG00000036452_KV390018	798748	800519	-1	8343	2630	CHRNG _ENSCCAG00000000722_KV390018.1	790405	797889	1	-1467	-34750	EIF4E2 _ENSCCAG00000019970_KV390018.1	800215	835269	1
Chimpanzee_Pan troglodytes	TIGD1 _ENSPTRG00000038833_2B	123615010	123616785	-1	8830	3549	CHRNG _ENSPTRG00000022726_2B	123606180	123613236	1	-2479	-33618	EIF4E2 _ENSPTRG00000013043_2B	123617489	123650403	1
Drill_Mandrillus leucophaeus	TIGD1 _ENSMLEG00000027320_KN974207.1	996103	997878	-1	8613	3867	CHRNG _ENSMLEG00000030051_KN974207.1	987490	994011	1	-1941	-40454	EIF4E2 _ENSMLEG00000036508_KN974207.1	998044	1038332	1
Gelada_Theropithecus gelada	TIGD1 _ENSTGEG00000019620_12	109176663	109178438	-1	8593	4465	CHRNG _ENSTGEG00000019606_12	109168070	109173973	1	-2493	-31886	EIF4E2 _ENSTGEG00000019774_12	109179156	109210324	1
Gibbon_Nomascus leucogenys	TIGD1 _ENSNLEG00000018314_22a	123699059	123700834	-1	8333	3541	CHRNG _ENSNLEG00000014319_22a	123690726	123697293	1	-1945	-30855	EIF4E2 _ENSNLEG00000014333_22a	123701004	123731689	1
Golden snub-nosed monkey_Rhinopithecus roxellana	TIGD1 _ENSRROG00000021399_KN299575.1	468010	469785	1	-4219	-9003	CHRNG _ENSRROG00000034175_KN299575.1	472229	478788	-1	35355	2473	EIF4E2 _ENSRROG00000043570_KN299575.1	432655	467312	-1
Gorilla_Gorilla gorilla gorilla	TIGD1 _ENSGGOG00000016685_2B	125896124	125898350	-1	8352	4026	CHRNG _ENSGGOG00000002100_2B	125887772	125894324	1	-2459	-33688	ENSGGOG00000043810_2B	125898583	125932038	1
Macaque_Macaca mulatta	TIGD1 _ENSMMUG00000022150_12	119972823	119975302	-1	8577	5132	CHRNG _ENSMMUG00000022149_12	119964246	119970170	1	-2569	-79393	EIF4E2 _ENSMMUG00000022151_12	119975392	120054695	1
Marmoset_Callithrix jacchus	TIGD1 _ENSCJAG00000013785_NTIC01035500.1	146892205	146893980	-1	8277	4055	CHRNG _ENSCJAG00000013767_NTIC01035500.1	146883928	146889925	1	-2622	-29871	EIF4E2 _ENSCJAG00000013786_NTIC01035500.1	146894827	146923851	1
Olive baboon_Papio anubis	TIGD1 _ENSPANG00000034102_12	118490011	118491783	-1	8585	4455	CHRNG _ENSPANG00000019942_12	118481426	118487328	1	-2613	-30644	EIF4E2 _ENSPANG00000015728_12	118492624	118522427	1
Orangutan_Pongo abelii	TIGD1 _ENSPPYG00000013281_2b	124956145	124957923	-1	8829	4177	CHRNG _ENSPPYG00000013280_2b	124947316	124953746	1	-2620	-34753	EIF4E2 _ENSPPYG00000013282_2b	124958765	124992676	1
Pig-tailed macaque_Macaca nemestrina	TIGD1 _ENSMNEG00000015811_KQ008994.1	10242023	10243798	1	-4458	-8609	CHRNG _ENSMNEG00000026483_KQ008994.1	10246481	10252407	-1	36589	2537	EIF4E2 _ENSMNEG00000038383_KQ008994.1	10205434	10241261	-1
Sooty mangabey_Cercocebus atys	TIGD1 _ENSCATG00000021481_KQ010141.1	4544857	4546632	-1	9120	3808	CHRNG _ENSCATG00000040459_KQ010141.1	4535737	4542824	1	-2468	-33019	EIF4E2 _ENSCATG00000035393_KQ010141.1	4547325	4579651	1
Ugandan red Colobus_Piliocolobus tephrosceles	TIGD1 _ENSPTEG00000033300_PDMG02000275.1	1112259	1114034	1	-4447	-8866	CHRNG _ENSPTEG00000033387_PDMG02000275.1	1116706	1122900	-1	30708	2610	ENSPTEG00000033277_PDMG02000275.1	1081551	1111424	-1
Vervet-AGM_Chlorocebus sabaeus	TIGD1 _ENSCSAG00000006471_10	118568654	118570785	-1	9424	5175	CHRNG _ENSCSAG00000006488_10	118559230	118565610	1	-2879	-36551	EIF4E2 _ENSCSAG00000006468_10	118571533	118607336	1
