# Supplementary material for: Phylogenetic analysis of the Tc1/mariner superfamily reveals the unexplored diversity of pogo-like elements
Source: Mob DNA. 2020 Jun 29;11:21. doi: 10.1186/s13100-020-00212-0 (PMC7325037; doi:10.1186/s13100-020-00212-0)
Supplement: Supplementary file 6 — Additional files 6 to 12. Conserved locations of TIGD1 to TIGD7 in host vertebrate species and information about the upstream and downstream genes flanking them, retrieved from Ensembl [54]. Negative numbers indicate that the considered gene is upstream of the TIGD element. [file 13100_2020_212_MOESM6_ESM.zip › 13100_2020_212_MOESM12_ESM.pdf]

Species	TIGD7 Ensembl name	Scaffold or chromosome	Pos. TIGD7 1 (bp)	Pos. TIGD7 2 (bp)	Sense	Dist. ZNF263 1 (bp)	Dist. ZNF263 2 (bp)	ZNF263 Ensembl name	Scaffold or chromosome	Pos. ZNF263 1 (bp)	Pos. ZNF263 2 (bp)	Sense	Dist. ZNF75A 1 (bp)	Dist. ZNF75A 2 (bp)	ZNF75A Ensembl name	Scaffold or chromosome	Pos. ZNF75A 1 (bp)	Pos. ZNF75A 2 (bp)	Sense
Alpine marmot_Marmota marmota marmota	ENSMMMG00000007488	CZRN01000029.1	6215932	6217581	1	-9027	-13544	ENSMMMG00000007494	CZRN01000029.1	6224959	6231125	-1	12313	10078	ENSMMMG00000007478	CZRN01000029.1	6203619	6207503	-1
American beaver_Castor canadensis	ENSCCNG00000029804	MTKA01003566.1	61993	63645	-1	10988	7047	ENSCCNG00000029803	MTKA01003566.1	51005	56598	1	-13031	-14794	ENSCCNG00000029808	MTKA01003566.1	75024	78439	1
American mink_Neovison vison	ENSNVIG00000023360	FNWR01000112.1	2828757	2830403	-1	12912	9012	ENSNVIG00000023357	FNWR01000112.1	2815845	2821391	1	-7232	-13898	ENSNVIG00000023361	FNWR01000112.1	2835989	2844301	1
Angola colobus_Colobus angolensis palliatus	ENSCANG00000003663	KN981636.1	594396	596045	1	-9044	-16147	ENSCANG00000040010	KN981636.1	603440	612192	-1	17628	5143	ENSCANG00000015866	KN981636.1	576768	590902	-1
Arctic ground squirrel_Urocitellus parryii	ENSUPAG00010018886	QVIC01000038.1	4132073	4133722	-1	11543	7380	ENSUPAG00010018875	QVIC01000038.1	4120530	4126342	1	-10514	-14414	ENSUPAG00010018898	QVIC01000038.1	4142587	4148136	1
Black snub-nosed monkey_Rhinopithecus bieti	ENSRBIG00000038447	MCGX01005408.1	5644545	5646192	1	-9007	-16272	ENSRBIG00000028568	MCGX01005408.1	5653552	5662464	-1	17869	6085	ENSRBIG00000035942	MCGX01005408.1	5626676	5640107	-1
Bolivian squirrel monkey_Saimiri boliviensis boliviensis	ENSSBOG00000017900	JH378134.1	16709102	16710751	-1	20858	12843	ENSSBOG00000020677	JH378134.1	16688244	16697908	1	-6259	-22541	ENSSBOG00000024179	JH378134.1	16715361	16733292	1
Bonobo_Pan paniscus	ENSPPAG00000001013	16	3408055	3409704	-1	12513	5623	ENSPPAG00000032719	16	3395542	3404081	1	-6518	-18186	ENSPPAG00000029289	16	3414573	3427890	1
Bushbaby_Otolemur garnettii	ENSOGAG00000015891	GL873563.1	9737164	9738816	-1	18331	10951	ENSOGAG00000015888	GL873563.1	9718833	9727865	1	-10375	-14421	ENSOGAG00000025451	GL873563.1	9747539	9753237	1
Capuchin_Cebus capucinus imitator	ENSCCAG00000000574	KV389870.1	3352291	3353940	-1	19594	12703	ENSCCAG00000030140	KV389870.1	3332697	3341237	1	-6176	-18710	ENSCCAG00000014978	KV389870.1	3358467	3372650	1
Chacoan peccary_Catagonus wagneri	ENSCWAG00000005270	PVHT021183594.1	6658632	6660266	1	-8040	-12004	ENSCWAG00000005290	PVHT021183594.1	6666672	6672270	-1	12807	12525	ENSCWAG00000005204	PVHT021183594.1	6645825	6647741	-1
Chimpanzee_Pan troglodytes	ENSPTRG00000007693	16	3558843	3560492	-1	12033	5623	ENSPTRG00000007692	16	3546810	3554869	1	-6542	-17935	ENSPTRG00000007694	16	3565385	3578427	1
Crab-eating macaque_Macaca fascicularis	ENSMFAG00000013017	20	3567654	3569303	-1	16302	8761	ENSMFAG00000031121	20	3551352	3560542	1	-6756	-18226	ENSMFAG00000042242	20	3574410	3587529	1
Daurian ground squirrel_Spermophilus dauricus	ENSSDAG00000008140	KZ294073.1	2727704	2729353	1	-8739	-13053	ENSSDAG00000008143	KZ294073.1	2736443	2742406	-1	11234	10044	ENSSDAG00000008116	KZ294073.1	2716470	2719309	-1
Dingo_Canis lupus dingo	ENSCAFG00020025754	QKWQ01001090.1	24914657	24916303	1	-7892	-12069	ENSCAFG00020025756	QKWQ01001090.1	24922549	24928372	-1	110043	9660	ENSCAFG00020025748	QKWQ01001090.1	24804614	24906643	-1
Dog - Basenji_Canis lupus familiaris	ENSCAFG00030002840	6	39776594	39778240	-1	12067	7888	ENSCAFG00030002827	6	39764527	39770352	1	-6716	-13253	ENSCAFG00030002847	6	39783310	39791493	1
Dog - Great Dane_Canis lupus familiaris	ENSCAFG00040017404	6	37932869	37934515	1	-7891	-12067	ENSCAFG00040017409	6	37940760	37946582	-1	13190	6710	ENSCAFG00040017362	6	37919679	37927805	-1
Drill_Mandrillus leucophaeus	ENSMLEG00000012629	KN974540.1	1172537	1174186	1	-8766	-15979	ENSMLEG00000025027	KN974540.1	1181303	1190165	-1	18274	6782	ENSMLEG00000039690	KN974540.1	1154263	1167404	-1
Ferret_Mustela putorius furo	ENSMPUG00000020053	GL896925.1	13032447	13034093	1	12745	-12386	ENSMPUG00000015125	GL896925.1	13019702	13046479	-1	13032426	13032548	ENSMPUG00000010039	AEYP01113178.1	21	1545	1
Gelada_Theropithecus gelada	ENSTGEG00000025464	20	49744498	49746147	1	-9567	-16477	ENSTGEG00000025490	20	49754065	49762624	-1	19132	6987	ENSTGEG00000025451	20	49725366	49739160	-1
Gibbon_Nomascus leucogenys	ENSNLEG00000018448	18	101640701	101642350	1	-15192	-22407	ENSNLEG00000009603	18	101655893	101664757	-1	18097	6737	ENSNLEG00000009645	18	101622604	101635613	-1
Golden snub-nosed monkey_Rhinopithecus roxellana	ENSRROG00000009012	KN297538.1	901550	903994	1	-9530	-15572	ENSRROG00000040363	KN297538.1	911080	919566	-1	18370	6284	ENSRROG00000034222	KN297538.1	883180	897710	-1
Gorilla_Gorilla gorilla gorilla	ENSGGOG00000010475	16	3432191	3433840	-1	15638	9208	ENSGGOG00000010468	16	3416553	3424632	1	-6499	-22133	ENSGGOG00000023562	16	3438690	3455973	1
Greater bamboo lemur_Prolemur simus	ENSPSMG00000017338	MPIZ01000564.1	395632	397278	-1	15693	9004	ENSPSMG00000017333	MPIZ01000564.1	379939	388274	1	-10910	-12005	ENSPSMG00000017355	MPIZ01000564.1	406542	409283	1
Horse_Equus caballus	ENSECAG00000004461	13	40702987	40704636	1	-7697	-11756	ENSECAG00000024249	13	40710684	40716392	-1	82058	7164	ENSECAG00000023652	13	40620929	40697472	-1
Ma's night monkey_Aotus nancymaae	ENSANAG00000019936	KZ205780.1	1372041	1373690	1	-7518	-18211	ENSANAG00000020520	KZ205780.1	1379559	1391901	-1	17052	13739	ENSANAG00000013144	KZ205780.1	1354989	1359951	-1
Macaque_Macaca mulatta	ENSMMUG00000040952	20	3351908	3358786	-1	15632	-2990	ENSMMUG00000017973	20	3336276	3361776	1	-6983	-14835	ENSMMUG00000017975	20	3358891	3373621	1
Marmoset_Callithrix jacchus	ENSCJAG00000019621	NTIC01016668.1	1951823	1953472	-1	16313	9189	ENSCJAG00000019612	NTIC01016668.1	1935510	1944283	1	-14532	-18159	ENSCJAG00000036662	NTIC01016668.1	1966355	1971631	1
Meerkat_Suricata suricatta	ENSSSUG00005017085	8	2473291	2474985	-1	12312	7407	ENSSSUG00005017003	8	2460979	2467578	1	-4965	-11599	ENSSSUG00005017122	8	2478256	2486584	1
Megabat_Pteropus vampyrus	ENSPVAG00000001370	GeneScaffold_66	89048	90697	-1	11289	6645	ENSPVAG00000001368	GeneScaffold_66	77759	84052	1	-9472	-14875	ENSPVAG00000001371	GeneScaffold_66	98520	105572	1
Naked mole-rat male_Heterocephalus glaber	ENSHGLG00100003331	JH167942.1	702785	704584	1	-7269	-12889	ENSHGLG00100004171	JH167942.1	710054	717473	-1	19655	7273	ENSHGLG00100002887	JH167942.1	683130	697311	-1
Olive baboon_Papio anubis	ENSPANG00000008160	20	3103045	3104694	-1	16860	11242	ENSPANG00000017591	20	3086185	3093452	1	-14601	-17748	ENSPANG00000006903	20	3117646	3122442	1
Orangutan_Pongo abelii	ENSPPYG00000007039	16	3410093	3411742	-1	15297	7217	ENSPPYG00000007038	16	3394796	3404525	1	-19283	-20725	ENSPPYG00000007041	16	3429376	3432467	1
Pig_Sus scrofa	ENSSSCG00000037309	3	38893694	38900327	1	-12463	-13532	ENSSSCG00000007963	3	38906157	38913859	-1	67912	6646	ENSSSCG00000029285	3	38825782	38893681	-1
Polar bear_Ursus maritimus	ENSUMAG00000001013	KK498627.1	5206418	5208064	-1	11965	6701	ENSUMAG00000001008	KK498627.1	5194453	5201363	1	-6327	-47788	ENSUMAG00000001028	KK498627.1	5212745	5255852	1
Sheep_Ovis aries	ENSOARG00000001631	24	2742762	2744408	-1	11634	7753	ENSOARG00000001622	24	2731128	2736655	1	-7592	-33770	ENSOARG00000001671	24	2750354	2778178	1
Shrew_Sorex araneus	ENSSARG00000000233	GeneScaffold_5408	84361	85995	-1	14631	8168	ENSSARG00000000228	GeneScaffold_5408	69730	77827	1	-13163	-12630	ENSSARG00000000235	GeneScaffold_5408	97524	98625	1
Siberian musk deer_Moschus moschiferus	ENSMMSG00000008089	PVHU021072333.1	857074	858714	1	-6953	-11607	ENSMMSG00000008110	PVHU021072333.1	864027	870321	-1	37922	5369	ENSMMSG00000007963	PVHU021072333.1	819152	853345	-1
Tiger_Panthera tigris altaica	ENSPTIG00000008868	KE721566.1	157442	159088	-1	11691	7237	ENSPTIG00000014550	KE721566.1	145751	151851	1	-9927	-12637	ENSPTIG00000014575	KE721566.1	167369	171725	1
Ugandan red Colobus_Piliocolobus tephrosceles	ENSPTEG00000012006	PDMG02000287.1	1522143	1523792	1	-8862	-15045	ENSPTEG00000012052	PDMG02000287.1	1531005	1538837	-1	22983	12369	ENSPTEG00000011928	PDMG02000287.1	1499160	1511423	-1
Vervet-AGM_Chlorocebus sabaeus	ENSCSAG00000010056	5	3021104	3028888	-1	13799	14439	ENSCSAG00000010072	5	3007305	3014449	1	-8143	-11692	ENSCSAG00000010048	5	3029247	3040580	1
