# Supplementary material for: Phylogenetic analysis of the Tc1/mariner superfamily reveals the unexplored diversity of pogo-like elements
Source: Mob DNA. 2020 Jun 29;11:21. doi: 10.1186/s13100-020-00212-0 (PMC7325037; doi:10.1186/s13100-020-00212-0)
Supplement: Supplementary file 6 — Additional files 6 to 12. Conserved locations of TIGD1 to TIGD7 in host vertebrate species and information about the upstream and downstream genes flanking them, retrieved from Ensembl [54]. Negative numbers indicate that the considered gene is upstream of the TIGD element. [file 13100_2020_212_MOESM6_ESM.zip › 13100_2020_212_MOESM11_ESM.pdf]

Species	TIGD6 name and location	Position 1 (bp)	Position 2 (bp)	Sense	Dist. SLC26A2 1 (bp)	Dist. SLC26A2 2 (bp)	SLC26A2 name and location	Pos. SLC26A2 1 (bp)	Pos. SLC26A2 2 (bp)	Sense SLC26A2	Dist. HMGXB3 1 (bp)	Dist. HMGXB3 2 (bp)	HMGXB3 name and location	Position HMGXB3 1 (bp)	Position HMGXB3 2 (bp)	Sense HMGXB3
Alpine marmot_Marmota marmota marmota	TIGD6_ENSMMMG00000019320_CZRN01000001.1	80479925	80479925	-1	16769	12992	SLC26A2_ENSMMMG00000019313_CZRN01000001.1	80463156	80466933	1	-9596	-57986	HMGXB3_ENSMMMG00000019323_CZRN01000001.1	80489521	80537911	1
American black bear_Ursus americanus	TIGD6_ENSUAMG00000024652_LZNR01000711.1	108019	108019	-1	29045	11653	SLC26A2_ENSUAMG00000024651_LZNR01000711.1	78974	96366	1	-4512	-52296	HMGXB3_ENSUAMG00000024653_LZNR01000711.1	112531	160315	1
American mink_Neovison vison	TIGD6_ENSNVIG00000001345_FNWR01000019.1	4689451	4689451	1	-13417	-30210	SLC26A2_ENSNVIG00000001354_FNWR01000019.1	4702868	4719661	-1	48187	2928	HMGXB3_ENSNVIG00000001274_FNWR01000019.1	4641264	4686523	-1
Angola colobus_Colobus angolensis palliatus	TIGD6_ENSCANG00000015006_KN985879.1	4329168	4329168	-1	39922	17856	SLC26A2_ENSCANG00000042547_KN985879.1	4289246	4311312	1	-5082	-58837	HMGXB3_ENSCANG00000041395_KN985879.1	4334250	4388005	1
Arctic ground squirrel_Urocitellus parryii	TIGD6_ENSUPAG00010013551_QVIC01000030.1	9053715	9053715	-1	20479	13974	SLC26A2_ENSUPAG00010013538_QVIC01000030.1	9033236	9039741	1	-5614	-57376	HMGXB3_ENSUPAG00010013558_QVIC01000030.1	9059329	9111091	1
Armadillo_Dasypus novemcinctus	Nothing annotated_JH565448	368694	368694	-1	72467	12902	SLC26A2_ENSDNOG00000032277_JH565448.1	296227	355792	1	-7990	-75591	HMGXB3_ENSDNOG00000006128_JH565448.1	376684	444285	1
Black snub-nosed monkey_Rhinopithecus bieti	TIGD6_ENSRBIG00000023892_MCGX01003552.1	5925002	5925002	1	-16406	-52532	SLC26A2_ENSRBIG00000016061_MCGX01003552.1	5941408	5977534	-1	56634	3518	HMGXB3_ENSRBIG00000040563_MCGX01003552.1	5868368	5921484	-1
Bolivian squirrel monkey_Saimiri boliviensis boliviensis	TIGD6_ENSSBOG00000032889_JH378166.1	8856182	8856182	-1	40711	18118	SLC26A2_ENSSBOG00000031044_JH378166.1	8815471	8838064	1	-5510	-60741	HMGXB3_ENSSBOG00000020648_JH378166.1	8861692	8916923	1
Bonobo_Pan paniscus	TIGD6_ENSPPAG00000008619_5	151425306	151425306	-1	33464	12630	SLC26A2_ENSPPAG00000038310_5	151391842	151412676	1	-5138	-59719	HMGXB3_ENSPPAG00000008815_5	151430444	151485025	1
Bushbaby_Otolemur garnettii	Nothing annotated_GL873522	26079100	26079100	1	-18763	-23138	SLC26A2_ENSOGAG00000015997_GL873522.1	26097863	26102238	-1	76500	8624	HMGXB3_ENSOGAG00000004593_GL873522.1	26002600	26070476	-1
Capuchin_Cebus capucinus imitator	Nothing annotated_KV389497	5484415	5484415	1	-13000	-35200	SLC26A2_ENSCCAG00000020161_KV389497.1	5497415	5519615	-1	56644	7704	HMGXB3_ENSCCAG00000023989_KV389497.1	5427771	5476711	-1
Cat_Felis catus	TIGD6_ENSFCAG00000024582_A1	199177379	199177379	1	-13039	-18519	SLC26A2_ENSFCAG00000026045_A1	199190418	199195898	-1	50876	2790	HMGXB3_ENSFCAG00000003632_A1	199126503	199174589	-1
Chimpanzee_Pan troglodytes	TIGD6_ENSPTRG00000049458_5	150193333	150193333	-1	34755	12962	SLC26A2_ENSPTRG00000043485_5	150158578	150180371	1	-5257	-60330	HMGXB3_ENSPTRG00000017408_5	150198590	150253663	1
Common wombat_Vombatus ursinus	TIGD6_ENSVURG00010008246_UNPS02014863.1	32907342	32907342	1	-16400	-46835	SLC26A2_ENSVURG00010008248_UNPS02014863.1	32923742	32954177	-1	59043	2207	HMGXB3_ENSVURG00010008212_UNPS02014863.1	32848299	32905135	-1
Coquerel's sifaka_Propithecus coquereli	Nothing annotated_KQ025735	2957047	2957047	1	-12183	-32146	SLC26A2_ENSPCOG00000021285_KQ025735.1	2969230	2989193	-1	71932	3445	HMGXB3_ENSPCOG00000023148_KQ025735.1	2885115	2953602	-1
Crab-eating macaque_Macaca fascicularis	TIGD6_ENSMFAG00000028440_6	149341690	149341690	-1	52888	7386	SLC26A2_ENSMFAG00000042946_6	149288802	149334304	1	-3982	-59229	HMGXB3_ENSMFAG00000041979_6	149345672	149400919	1
Daurian ground squirrel_Spermophilus dauricus	TIGD6_ENSSDAG00000016436_KZ294318.1	176300	176300	1	-15275	-19056	SLC26A2_ENSSDAG00000016441_KZ294318.1	191575	195356	-1	62032	8077	HMGXB3_ENSSDAG00000016403_KZ294318.1	114268	168223	-1
Dingo_Canis lupus dingo	TIGD6_ENSCAFG00020023582_QKWQ01001938.1	7852739	7852739	-1	28070	11571	SLC26A2_ENSCAFG00020023581_QKWQ01001938.1	7824669	7841168	1	-4595	-50950	HMGXB3_ENSCAFG00020023583_QKWQ01001938.1	7857334	7903689	1
Dog_Canis lupus familiaris	TIGD6_ENSCAFG00000018238_4	59061372	59061372	1	-12636	-17520	SLC26A2_ENSCAFG00000018240_4	59074008	59078892	-1	49589	-17520	HMGXB3_ENSCAFG00000018231_4	59011783	59078892	-1
Donkey_Equus asinus asinus	TIGD6_ENSEASG00005002776_PSZQ01000517.1	14289935	14289935	1	-14478	-20562	SLC26A2_ENSEASG00005002777_PSZQ01000517.1	14304413	14310497	-1	46457	6052	HMGXB3_ENSEASG00005002772_PSZQ01000517.1	14243478	14283883	-1
Drill_Mandrillus leucophaeus	TIGD6_ENSMLEG00000000354_KN975005.1	2167265	2167265	1	-14533	-41407	SLC26A2_ENSMLEG00000026088_KN975005.1	2181798	2208672	-1	56725	3886	HMGXB3_ENSMLEG00000043365_KN975005.1	2110540	2163379	-1
Elephant_Loxodonta africana	Nothing annotated_scaffold_1	69488151	69488151	-1	20115	16030	SLC26A2_ENSLAFG00000029084_scaffold_1	69468036	69472121	1	-8677	-57935	HMGXB3_ENSLAFG00000016790_scaffold_1	69496828	69546086	1
Ferret_Mustela putorius furo	TIGD6_ENSMPUG00000019320_GL896898.1	28487450	28487450	1	-3355	-30023	SLC26A2_ENSMPUG00000014113_GL896898.1	28490805	28517473	-1	47248	2707	HMGXB3_ENSMPUG00000014009_GL896898.1	28440202	28484743	-1
Gelada_Theropithecus gelada	TIGD6_ENSTGEG00000002178_6	146459209	146459209	-1	39950	10276	SLC26A2_ENSTGEG00000002165_6	146419259	146448933	1	-5767	-58682	HMGXB3_ENSTGEG00000002263_6	146464976	146517891	1
Gibbon_Nomascus leucogenys	TIGD6_ENSNLEG00000010212_2	31047330	31047330	1	-14442	-36207	SLC26A2_ENSNLEG00000010209_2	31061772	31083537	-1	66367	4054	HMGXB3_ENSNLEG00000010213_2	30980963	31043276	-1
Goat_Capra hircus	TIGD6_ENSCHIG00000026767_7	49102493	49102493	1	-14457	-18141	SLC26A2_ENSCHIG00000012265_7	49116950	49120634	-1	70002	6501	HMGXB3_ENSCHIG00000011628_7	49032491	49095992	-1
Golden snub-nosed monkey_Rhinopithecus roxellana	TIGD6_ENSRROG00000019927_KN297153.1	24796	24796	-1	18211	14002	SLC26A2_ENSRROG00000012031_KN297153.1	6585	10794	1	-5087	-60400	HMGXB3_ENSRROG00000032246_KN297153.1	29883	85196	1
Gorilla_Gorilla gorilla gorilla	TIGD6_ENSGGOG00000008920_5	133689307	133689307	-1	35736	14815	SLC26A2_ENSGGOG00000000528_5	133653571	133674492	1	-5099	-58300	HMGXB3_ENSGGOG00000008921_5	133694406	133747607	1
Greater bamboo lemur_Prolemur simus	TIGD6_ENSPSMG00000024090_MPIZ01000052.1	1457727	1457727	1	-10058	-13843	SLC26A2_ENSPSMG00000024091_MPIZ01000052.1	1467785	1471570	-1	60212	3727	HMGXB3_ENSPSMG00000024071_MPIZ01000052.1	1397515	1454000	-1
Horse_Equus caballus	TIGD6_ENSECAG00000004664_14	27283864	27283864	1	-14484	-20562	SLC26A2_ENSECAG00000023363_14	27298348	27304426	-1	47388	6049	HMGXB3_ENSECAG00000015743_14	27236476	27277815	-1
Hyrax_Procavia capensis	TIGD6_ENSPCAG00000016618_GeneScaffold_4690	25519	25519	-1	16057	12333	SLC26A2_ENSPCAG00000016579_GeneScaffold_4690	9462	13186	1	-5422	-68334	HMGXB3_ENSPCAG00000016669_GeneScaffold_4690	30941	93853	1
Koala_Phascolarctos cinereus	TIGD6_ENSPCIG00000016474_MSTS01000152.1	4474811	4474811	-1	44666	14641	SLC26A2_ENSPCIG00000016473_MSTS01000152.1	4430145	4460170	1	-10091	-63140	HMGXB3_ENSPCIG00000013139_MSTS01000152.1	4484902	4537951	1
Leopard_Panthera pardus	TIGD6_ENSPPRG00000015925_KV860316.1	13365128	13365128	-1	26617	9829	SLC26A2_ENSPPRG00000022664_KV860316.1	13338511	13355299	1	-4807	-54083	HMGXB3_ENSPPRG00000023078_KV860316.1	13369935	13419211	1
Ma's night monkey_Aotus nancymaae	TIGD6_ENSANAG00000023656_KZ203792.1	5115332	5115332	1	-14356	-18810	SLC26A2_ENSANAG00000024045_KZ203792.1	5129688	5134142	-1	56823	3383	HMGXB3_ENSANAG00000021091_KZ203792.1	5058509	5111949	-1
Macaque_Macaca mulatta	TIGD6_ENSMMUG00000011553_6	147506633	147506633	-1	40481	11301	SLC26A2_ENSMMUG00000029776_6	147466152	147495332	1	-6439	-59472	HMGXB3_ENSMMUG00000011554_6	147513072	147566105	1
Megabat_Pteropus vampyrus	TIGD6_ENSPVAG00000001311_GeneScaffold_2377	192503	192503	-1	12482	8475	SLC26A2_ENSPVAG00000001310_GeneScaffold_2377	180021	184028	1	-4520	-46605	HMGXB3_ENSPVAG00000001312_GeneScaffold_2377	197023	239108	1
Naked mole-rat female_Heterocephalus glaber	Nothing annotated_JH602087	9282504	9282504	-1	24822	12751	SLC26A2_ENSHGLG00000008518_JH602087.1	9257682	9269753	1	-12108	-79597	HMGXB3_ENSHGLG00000009767_JH602087.1	9294612	9362101	1
Naked mole-rat male_Heterocephalus glaber	Nothing annotated_JH173150	446435	446435	1	-12891	-28565	SLC26A2_ENSHGLG00100009316_JH173150.1	459326	475000	-1	77987	10269	HMGXB3_ENSHGLG00100009399_JH173150.1	368448	436166	-1
Olive baboon_Papio anubis	TIGD6_ENSPANG00000023729_6	144389460	144389460	-1	39919	12993	SLC26A2_ENSPANG00000008875_6	144349541	144376467	1	-3969	-56977	HMGXB3_ENSPANG00000023318_6	144393429	144446437	1
Opossum_Monodelphis domestica	Nothing annotated_1	342814280	342814280	-1	50212	23286	SLC26A2_ENSMODG00000045345_1	342764068	342790994	1	-11682	-63336	HMGXB3_ENSMODG00000009798_1	342825962	342877616	1
Orangutan_Pongo abelii	Nothing annotated_5	151940429	151940429	-1	35332	8651	SLC26A2_ENSPPYG00000015940_5	151905097	151931778	1	-5875	-57969	HMGXB3_ENSPPYG00000015941_5	151946304	151998398	1
Panda_Ailuropoda melanoleuca	TIGD6_ENSAMEG00000020121_GL192580.1	767447	767447	1	-11112	-16926	SLC26A2_ENSAMEG00000005206_GL192580.1	778559	784373	-1	47599	7451	HMGXB3_ENSAMEG00000005161_GL192580.1	719848	759996	-1
Pig-tailed macaque_Macaca nemestrina	TIGD6_ENSMNEG00000028353_KQ007139.1	3394948	3394948	1	-13551	-45354	SLC26A2_ENSMNEG00000028522_KQ007139.1	3408499	3440302	-1	56499	4099	HMGXB3_ENSMNEG00000035203_KQ007139.1	3338449	3390849	-1
Polar bear_Ursus maritimus	TIGD6_ENSUMAG00000004793_KK498603.1	22734995	22734995	1	-12832	-29729	SLC26A2_ENSUMAG00000004796_KK498603.1	22747827	22764724	-1	46372	5694	HMGXB3_ENSUMAG00000004789_KK498603.1	22688623	22729301	-1
Rabbit_Oryctolagus cuniculus	Nothing annotated_3	31773727	31773727	-1	31531	10739	SLC26A2_ENSOCUG00000013871_3	31742196	31762988	1	-5874	-60659	HMGXB3_ENSOCUG00000007500_3	31779601	31834386	1
Red fox_Vulpes vulpes	TIGD6_ENSVVUG00000007540_NBDQ01000003.1	40313981	40313981	1	-12720	-29237	SLC26A2_ENSVVUG00000007543_NBDQ01000003.1	40326701	40343218	-1	50102	2873	HMGXB3_ENSVVUG00000007485_NBDQ01000003.1	40263879	40311108	-1
Sooty mangabey_Cercocebus atys	TIGD6_ENSCATG00000017990_KQ009929.1	2999855	2999855	-1	40207	12973	SLC26A2_ENSCATG00000016803_KQ009929.1	2959648	2986882	1	-5445	-57754	HMGXB3_ENSCATG00000038002_KQ009929.1	3005300	3057609	1
Squirrel_Ictidomys tridecemlineatus	TIGD6_ENSSTOG00000008758_JH393314.1	4981278	4981278	1	-9802	-24599	SLC26A2_ENSSTOG00000006553_JH393314.1	4991080	5005877	-1	55285	7970	HMGXB3_ENSSTOG00000008763_JH393314.1	4925993	4973308	-1
Tasmanian devil_Sarcophilus harrisii	TIGD6_ENSSHAG00000016809_GL834603.1	2356979	2356979	-1	23673	19523	SLC26A2_ENSSHAG00000016766_GL834603.1	2333306	2337456	1	-3850	-57019	HMGXB3_ENSSHAG00000016817_GL834603.1	2360829	2413998	1
Tiger_Panthera tigris altaica	TIGD6_ENSPTIG00000006085_KE722044.1	508193	508193	-1	27391	9860	SLC26A2_ENSPTIG00000016543_KE722044.1	480802	498333	1	-5049	-52320	HMGXB3_ENSPTIG00000017751_KE722044.1	513242	560513	1
Ugandan red Colobus_Piliocolobus tephrosceles	TIGD6_ENSPTEG00000019809_PDMG02000084.1	14146907	14146907	1	-14847	-41478	SLC26A2_ENSPTEG00000019822_PDMG02000084.1	14161754	14188385	-1	56320	3315	HMGXB3_ENSPTEG00000019789_PDMG02000084.1	14090587	14143592	-1
Vervet-AGM_Chlorocebus sabaeus	TIGD6_ENSCSAG00000019718_23	52605619	52605619	-1	40210	12962	SLC26A2_ENSCSAG00000011710_23	52565409	52592657	1	-5751	-56574	HMGXB3_ENSCSAG00000011700_23	52611370	52662193	1
