# Supplementary material for: Phylogenetic analysis of the Tc1/mariner superfamily reveals the unexplored diversity of pogo-like elements
Source: Mob DNA. 2020 Jun 29;11:21. doi: 10.1186/s13100-020-00212-0 (PMC7325037; doi:10.1186/s13100-020-00212-0)
Supplement: Supplementary file 6 — Additional files 6 to 12. Conserved locations of TIGD1 to TIGD7 in host vertebrate species and information about the upstream and downstream genes flanking them, retrieved from Ensembl [54]. Negative numbers indicate that the considered gene is upstream of the TIGD element. [file 13100_2020_212_MOESM6_ESM.zip › 13100_2020_212_MOESM10_ESM.pdf]

Species	TIGD5 Ensembl name	Scaffold or chromosome	Pos. TIGD5 1 (bp)	Pos. TIGD5 2 (bp)	Sense	Dist. EEF1D 1 (bp)	Dist. EEF1D 2 (bp)	EEF1D Ensembl name	Scaffold or chromosome	Pos. EEF1D 1 (bp)	Pos. EEF1D 2 (bp)	Sense	Dist. PYCR3 1 (bp)	Dist. PYCR3 2 (bp)	PYCR3 Ensembl name	Scaffold or chromosome	Pos. PYCR3 1 (bp)	Pos. PYCR3 2 (bp)	Sense
Algerian mouse Mus spretus	MGP SPRETEiJ G0020802	15	75144806	75149845	1	14746	5218	MGP SPRETEiJ G0020801	15	75130060	75144627	-1	-6969	-6957	MGP SPRETEiJ G0020803	15	75151775	75156802	-1
American beaver Castor canadensis	ENSCCNG00000028237	MTKA01001733.1	488426	490384	-1	-2117	-15555	ENSCCNG00000028239	MTKA01001733.1	490543	505939	1	8682	5705	ENSCCNG00000028233	MTKA01001733.1	479744	484679	1
American black bear Ursus americanus	ENSUAMG00000012895	LZNR01001047.1	115831	120079	-1	-11981	-15651	ENSUAMG00000012897	LZNR01001047.1	127812	135730	1	6020	4993	ENSUAMG00000012891	LZNR01001047.1	109811	115086	1
Arabian camel Camelus dromedarius	ENSCDRG00005015329	25	39676154	39678855	1	14317	2183	ENSCDRG00005015097	25	39661837	39676672	-1	-6494	-8199	ENSCDRG00005015342	25	39682648	39687054	-1
Arctic ground squirrel Urocitellus parryii	ENSUPAG00010023459	QVIC01000438.1	398558	400750	1	15475	7632	ENSUPAG00010023458	QVIC01000438.1	383083	393118	-1	-5941	-9478	ENSUPAG00010023460	QVIC01000438.1	404499	410228	-1
Black snub-nosed monkey Rhinopithecus bieti	ENSRBIG00000039272	MCGX01004891.1	764528	766500	1	18590	2182	ENSRBIG00000044433	MCGX01004891.1	745938	764318	-1	-6197	-10191	ENSRBIG00000042874	MCGX01004891.1	770725	776691	-1
Bonobo Pan paniscus	ENSPPAG00000028001	8	143366390	143368240	1	25462	5269	ENSPPAG00000019348	8	143340928	143362971	-1	-5358	-9792	ENSPPAG00000028398	8	143371748	143378032	-1
Bushbaby Otolemur garnettii	ENSOGAG00000024882	GL873764.1	1297371	1299317	-1	-8524	-17664	ENSOGAG00000014938	GL873764.1	1305895	1316981	1	8474	6964	ENSOGAG00000026290	GL873764.1	1288897	1292353	1
Capuchin Cebus capucinus imitator	ENSCCAG00000019541	KV389875.1	2314048	2315970	1	18216	2160	ENSCCAG00000027651	KV389875.1	2295832	2313810	-1	-6167	-13708	ENSCCAG00000024608	KV389875.1	2320215	2329678	-1
Cat Felis catus	ENSFCAG00000001039	F2	84683017	84685434	1	13976	8796	ENSFCAG00000027237	F2	84669041	84676638	-1	-6637	-8127	ENSFCAG00000001040	F2	84689654	84693561	-1
Chacoan peccary Catagonus wagneri	ENSCWAG00000008332	PVHT021183677.1	21594	23507	1	12672	2329	ENSCWAG00000008255	PVHT021183677.1	8922	21178	-1	-4635	-4624	ENSCWAG00000008347	PVHT021183677.1	26229	28131	-1
Chilean tinamou Nothoprocta perdicaria	ENSNPEG00000015813	PTEW01000049.1	4465889	4468224	1	18540	10639	ENSNPEG00000015812	PTEW01000049.1	4447349	4457585	-1	-4633	-5610	ENSNPEG00000015814	PTEW01000049.1	4470522	4473834	-1
Chimpanzee Pan troglodytes	ENSPTRG00000030911	8	146362900	146364831	1	18160	4883	ENSPTRG00000020660	8	146344740	146359948	-1	-5446	-9791	ENSPTRG00000020661	8	146368346	146374622	-1
Chinese hamster CHOK1GS Cricetulus griseus	ENSCGRG00001010766	scaffold 0	136468696	136470621	1	14099	7414	ENSCGRG00001014276	scaffold 0	136454597	136463207	-1	-7611	-9678	ENSCGRG00001022822	scaffold 0	136476307	136480299	-1
Chinese hamster CriGri Cricetulus griseus	ENSCGRG00000019303	JH001301.1	11352	13106	-1	-2207	-15415	ENSCGRG00000007914	JH001301.1	13559	28521	1	9684	6578	ENSCGRG00000007454	JH001301.1	1668	6528	1
Chinese hamster PICR Cricetulus griseus	ENSCGRG00015017958	RAZU01000104.1	3326002	3327927	1	15090	2064	ENSCGRG00015017729	RAZU01000104.1	3310912	3325863	-1	-6771	-10001	ENSCGRG00015017966	RAZU01000104.1	3332773	3337928	-1
Cow Bos taurus	ENSBTAG00000048160	14	1111425	1113356	-1	-2126	-13000	ENSBTAG00000014643	14	1113551	1126356	1	9823	4622	ENSBTAG00000016810	14	1101602	1108734	1
Damara mole rat Fukomys damarensis	ENSFDAG00000011204	KN122397.1	6761669	6763357	-1	-2615	-16681	ENSFDAG00000010866	KN122397.1	6764284	6780038	1	9477	6191	ENSFDAG00000018800	KN122397.1	6752192	6757166	1
Dog - Basenji Canis lupus familiaris	ENSCAFG00030024193	13	36982512	36984464	1	13827	1951	ENSCAFG00030024114	13	36968685	36982513	-1	-5931	-8039	ENSCAFG00030024203	13	36988443	36992503	-1
Dog - Great Dane Canis lupus familiaris	ENSCAFG00040012141	13	37400416	37402368	1	13763	1902	ENSCAFG00040011929	13	37386653	37400466	-1	-5933	-8030	ENSCAFG00040012168	13	37406349	37410398	-1
Dolphin Tursiops truncatus	ENSTTRG00000015171	GeneScaffold 63	110630	112869	1	15814	9058	ENSTTRG00000015166	GeneScaffold 63	94816	103811	-1	-6993	-8344	ENSTTRG00000015176	GeneScaffold 63	117623	121213	-1
Domestic yak Bos grunniens	ENSBGRG00000005583	18	20717994	20719925	1	13810	10589	ENSBGRG00000005433	18	20704184	20709336	-1	-5705	-5289	ENSBGRG00000005589	18	20723699	20725214	-1
Donkey Equus asinus asinus	ENSEASG00005009657	PSZQ01006646.1	549296	551236	1	16883	2164	ENSEASG00005009652	PSZQ01006646.1	532413	549072	-1	-7267	-9282	ENSEASG00005009658	PSZQ01006646.1	556563	560518	-1
Elephant Loxodonta africana	ENSLAFG00000010678	scaffold 146	686754	688229	-1	-8801	-16838	ENSLAFG00000029200	scaffold 146	695555	705067	1	6821	5453	ENSLAFG00000010682	scaffold 146	679933	682776	1
Goat Capra hircus	ENSCHIG00000012655	14	80933518	80935440	1	12895	5607	ENSCHIG00000011578	14	80920623	80929833	-1	-5077	-9964	ENSCHIG00000016380	14	80938595	80945404	-1
Golden Hamster Mesocricetus auratus	ENSMAUG00000005967	KB708338.1	562619	564523	1	14591	8319	ENSMAUG00000000730	KB708338.1	548028	556204	-1	-6861	-9610	ENSMAUG00000018085	KB708338.1	569480	574133	-1
Gorilla Gorilla gorilla gorilla	ENSGGOG00000002392	8	145224384	145226315	1	23296	2292	ENSGGOG00000002389	8	145201088	145224023	-1	-6322	-9711	ENSGGOG00000002393	8	145230706	145236026	-1
Greater bamboo lemur Prolemur simus	ENSPSMG00000023915	MPIZ01001150.1	87157	89115	-1	-8771	-19855	ENSPSMG00000023968	MPIZ01001150.1	95928	108970	1	15178	7470	ENSPSMG00000023910	MPIZ01001150.1	71979	81645	1
Greater horseshoe bat Rhinolophus ferrumequinum	ENSRFEG00010017037	14	72630323	72632218	1	14821	8307	ENSRFEG00010016844	14	72615502	72623911	-1	-6468	-8285	ENSRFEG00010017055	14	72636791	72640503	-1
Guinea Pig Cavia porcellus	ENSCPOG00000039267	DS562950.1	2724901	2726838	1	13954	8123	ENSCPOG00000000048	DS562950.1	2710947	2718715	-1	-5874	-10548	ENSCPOG00000039640	DS562950.1	2730775	2737386	-1
Horse Equus caballus	ENSECAG00000030758	9	84419608	84421548	1	15898	1961	ENSECAG00000007976	9	84403710	84419587	-1	-7271	-9326	ENSECAG00000019384	9	84426879	84430874	-1
Hybrid - Bos Indicus Bos indicus x Bos taurus	ENSBIXG00005030024	14	1059110	1061041	-1	-624	-12999	ENSBIXG00005030062	14	1059734	1074040	1	9863	4622	ENSBIXG00005030011	14	1049247	1056419	1
Hybrid - Bos Taurus Bos indicus x Bos taurus	ENSBIXG00000012913	14	81170502	81172433	1	13000	624	ENSBIXG00000012942	14	81157502	81171809	-1	-4622	-9834	ENSBIXG00000012909	14	81175124	81182267	-1
Hyrax Procavia capensis	ENSPCAG00000005650	scaffold 12078	25802	27517	1	13910	6882	ENSPCAG00000005528	scaffold 12078	11892	20635	-1	-5357	-6047	ENSPCAG00000005745	scaffold 12078	31159	33564	-1
Kangaroo rat Dipodomys ordii	ENSDORG00000025658	KN672525.1	225410	282427	1	-41038	7328	ENSDORG00000000593	KN672525.1	266448	275099	-1	-60110	-7246	ENSDORG00000000595	KN672525.1	285520	289673	-1
Leopard Panthera pardus	ENSPPRG00000018004	KV860444.1	554978	556927	1	14571	2402	ENSPPRG00000007970	KV860444.1	540407	554525	-1	-6657	-8791	ENSPPRG00000007124	KV860444.1	561635	565718	-1
Long-tailed chinchilla Chinchilla lanigera	ENSCLAG00000018021	JH721914.1	2299640	2301574	1	14748	8673	ENSCLAG00000008571	JH721914.1	2284892	2292901	-1	-5845	-9706	ENSCLAG00000010276	JH721914.1	2305485	2311280	-1
Marmoset Callithrix jacchus	ENSCJAG00000021417	NTIC01007779.1	45032821	45034752	1	19341	1795	ENSCJAG00000021419	NTIC01007779.1	45013480	45032957	-1	-7422	-8999	ENSCJAG00000021413	NTIC01007779.1	45040243	45043751	-1
Megabat Pteropus vampyrus	ENSPVAG00000005993	GeneScaffold 80	102084	103624	1	15063	8459	ENSPVAG00000005990	GeneScaffold 80	87021	95165	-1	-5711	-8460	ENSPVAG00000005997	GeneScaffold 80	107795	112084	-1
Mongolian gerbil Meriones unguiculatus	ENSMUGG00000009915	NHTI01000038.1	1249339	1251273	1	14978	2156	ENSMUGG00000009815	NHTI01000038.1	1234361	1249117	-1	-7355	-10473	ENSMUGG00000009919	NHTI01000038.1	1256694	1261746	-1
Mouse Mus musculus	ENSMUSG00000103906	15	75909735	75914535	1	15530	4979	ENSMUSG00000055762	15	75894205	75909556	-1	-6742	-7025	ENSMUSG00000022571	15	75916477	75921560	-1
Northern American deer mouse Peromyscus maniculatus bairdii	ENSPEMG00000006475	20	41631180	41633105	1	14807	8052	ENSPEMG00000012989	20	41616373	41625053	-1	-6476	-8857	ENSPEMG00000008129	20	41637656	41641962	-1
Panda Ailuropoda melanoleuca	ENSAMEG00000006595	GL192891.1	1079846	1084203	-1	-8765	-12036	ENSAMEG00000006602	GL192891.1	1088611	1096239	1	8418	8795	ENSAMEG00000006577	GL192891.1	1071428	1075408	1
Pig-tailed macaque Macaca nemestrina	ENSMNEG00000016713	KQ005094.1	4264939	4266867	1	18725	2125	ENSMNEG00000042392	KQ005094.1	4246214	4264742	-1	-6338	-9654	ENSMNEG00000003395	KQ005094.1	4271277	4276521	-1
Pig Sus scrofa	ENSSSCG00000040119	4	966684	969283	-1	-2840	-17467	ENSSSCG00000006954	4	969524	986750	1	6976	5088	ENSSSCG00000032763	4	959708	964195	1
Ryukyu mouse Mus caroli	MGP CAROLIEiJ G0019902	15	69864583	69869388	1	15248	4984	MGP CAROLIEiJ G0019901	15	69849335	69864404	-1	-6787	-7076	MGP CAROLIEiJ G0019903	15	69871370	69876464	-1
Sheep Ovis aries	ENSOARG00000000069	9	14057009	14060879	-1	-9871	-11067	ENSOARG00000000129	9	14066880	14071946	1	10024	7101	ENSOARG00000016696	9	14046985	14053778	1
Shrew mouse Mus pahari	MGP PahariEiJ G0019910	17	40941929	40946820	1	15352	5068	MGP PahariEiJ G0019909	17	40926577	40941752	-1	-6840	-7401	MGP PahariEiJ G0019911	17	40948769	40954221	-1
Siberian musk deer Moschus moschiferus	ENSMMSG00000018919	PVHU021072404.1	978301	980229	1	13088	2096	ENSMMSG00000018709	PVHU021072404.1	965213	978133	-1	-4638	-8344	ENSMMSG00000018935	PVHU021072404.1	982939	988573	-1
Sooty mangabey Cercocebus atys	ENSCATG00000021269	KQ012608.1	4182036	4183967	1	18224	4734	ENSCATG00000028384	KQ012608.1	4163812	4179233	-1	-6807	-10119	ENSCATG00000031176	KQ012608.1	4188843	4194086	-1
Sperm whale Physeter catodon	ENSPCTG00005013135	15	869837	871765	-1	-1E+08	-1E+08	ENSPCTG00005013032	14	98646768	98647592	1	5980	5741	ENSPCTG00005013092	15	863857	866024	1
Steppe mouse Mus spicilegus	ENSMSIG00000021495	QGOO01037087.1	556492	558420	1	14726	2287	ENSMSIG00000021333	QGOO01037087.1	541766	556133	-1	-6965	-10110	ENSMSIG00000021499	QGOO01037087.1	563457	568530	-1
Ugandan red Colobus Piliocolobus tephrosceles	ENSPTEG00000025896	PDMG02001896.1	1483390	1485174	-1	-6795	-24212	ENSPTEG00000026054	PDMG02001896.1	1490185	1509386	1	9576	7627	ENSPTEG00000025869	PDMG02001896.1	1473814	1477547	1
Upper Galilee mountains blind mole rat Nannospalax galili	ENSNGAG00000016409	KL201069.1	1292806	1294768	-1	-2407	-14759	ENSNGAG00000020992	KL201069.1	1295213	1309527	1	9468	7203	ENSNGAG00000020709	KL201069.1	1283338	1287565	1
Vervet-AGM Chlorocebus sabaeus	ENSCSAG00000018635	8	137818489	137820420	1	19061	7300	ENSCSAG00000008996	8	137799428	137813120	-1	-7804	-10126	ENSCSAG00000008980	8	137826293	137830546	-1
