# Supplementary material for: Phylogenetic analysis of the Tc1/mariner superfamily reveals the unexplored diversity of pogo-like elements
Source: Mob DNA. 2020 Jun 29;11:21. doi: 10.1186/s13100-020-00212-0 (PMC7325037; doi:10.1186/s13100-020-00212-0)
Supplement: Supplementary file 9 — Additional File 15. Table showing the results of assessing the copy number of full-length mariner and pogo elements in the Hydra vulgaris genome. [file 13100_2020_212_MOESM15_ESM.pdf]

Species	Family	Number in tree	Element used	BLASTn hits (full-length)
Hydra vulgaris	*mariner*	14	Mariner-16_HM	169
Bactrocera tryoni	*mariner*	7	*Batmar3*	87
Hydra vulgaris	*pogo*	93	Mariner-10 and 18_HM	34
Aspergillus nidulans	*pogo*	15	Mariner-3_AN	10
