# Supplementary material for: Phylogenetic analysis of the Tc1/mariner superfamily reveals the unexplored diversity of pogo-like elements
Source: Mob DNA. 2020 Jun 29;11:21. doi: 10.1186/s13100-020-00212-0 (PMC7325037; doi:10.1186/s13100-020-00212-0)
Supplement: Supplementary file 2 — Additional file 2. Phylogenetic tree of the amino-acid DDD/E transposase domain of 5158 Tc1/mariner elements. The tree results from a phylogenetic analysis using maximum likelihood inference, with 1000 bootstrap repetitions. Clade support values above 70% are indicated adjacent to each clade. Clades are divided into groups, with a corresponding clade name and number to the right. Elements are named according to their Repbase or Genbank ID, or according to the name provided in the article describing them. The host genome for each element is indicated to the right hand side of its ID, and labels are coloured broadly according to the taxonomic kingdom and class that the host species belongs to: shades of blue for metazoans, purple for excavates (Parabasalids), dark red for oomycetes (Stramenopiles), yellow for amoebozoans, shades of orange for fungi (pink for Basidiomycota), and shades of green for plants. [file 13100_2020_212_MOESM2_ESM.pdf]

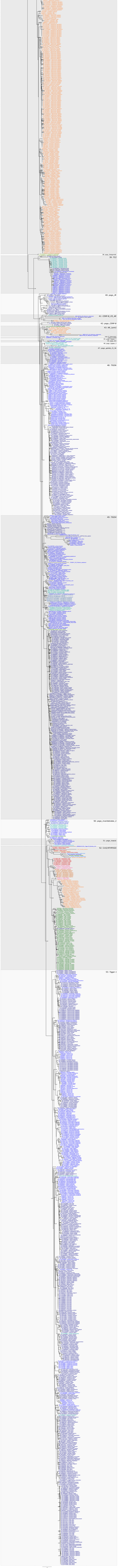

38: pogo_Selaginella
39: TC2
40: pogo_JR
41: CENP-B_CR_AR
42: pogo_CENP-B
43: AR_Lemi1
pogo_M. destructans
pogo_O. cincta
44: pogo_Cgigas_1
45: pogo_Dm
46: pogo_Cgigas_2
47: pogo_aphids_Ct_Sk
48: TIGD6
49: TIGD4
50: pogo_invertebrates_2
51: pogo_insects
52: Cirt2/ATHPOGO
53: Tigger 1
