# Supplementary material for: Phylogenetic analysis of the Tc1/mariner superfamily reveals the unexplored diversity of pogo-like elements
Source: Mob DNA. 2020 Jun 29;11:21. doi: 10.1186/s13100-020-00212-0 (PMC7325037; doi:10.1186/s13100-020-00212-0)
Supplement: Supplementary file 8 — Additional File 14. Phylogenetic tree of the amino-acid DDD/E transposase domain of TIGD-like elements. The tree results from a phylogenetic analysis using maximum likelihood inference, with 1000 bootstrap repetitions. Clades are divided into groups, with a corresponding clade name and number to the right. The host genome for each element is indicated to the right hand side of its ID, and labels are coloured broadly according to the taxonomic kingdom and class that the host species belongs to: shades of blue for metazoans, purple for excavates (Parabasalids), dark red for oomycetes (Stramenopiles), yellow for amoebozoans, shades of orange for fungi (pink for Basidiomycota), and shades of green for plants. [file 13100_2020_212_MOESM14_ESM.pdf]

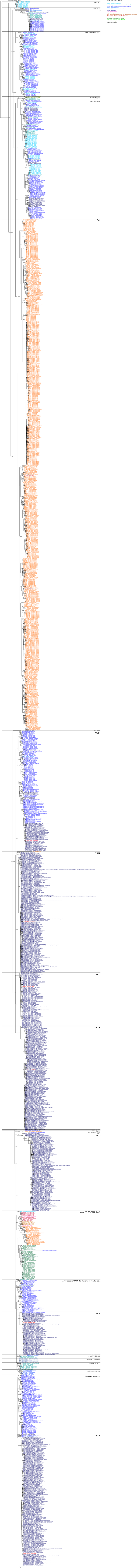

Key to host associations:
Animals - Cnidaria and Porifera
Animals - Lophotrochozoa (Mollusca, Annelida, Rotifera)
Animals - Ecdysozoa (Nematoda and Panarthropoda)
Animals - Platyhelminthes
Animals - Echinodermata and Hemichordata
Animals - Chordata
Fungi - Basidiomycota
Fungi - Ascomycota
Fungi - unranked (Mucoromycota, Microsporidia, Glomeromycota)
Stramenopiles - Oomycota and Bacillariophyta
Amoebozoa
Viridiplantae - Magnoliophyta: Dicots
Viridiplantae - Magnoliophyta: Monocots
Viridiplantae - Lycopodiopsida
Prokaryotes - Bacteria
pogo_Hv
pogo_JR_CR
TC2
invertebrates_1
GEMINI_E. invadens
pogo_Selaginella_Sm
pogo_Cgigas
pogo_Metazoa
